# Physiological, Ultrastructural and Proteomic Responses in the Leaf of Maize Seedlings to Polyethylene Glycol-Stimulated Severe Water Deficiency

**DOI:** 10.3390/ijms160921606

**Published:** 2015-09-08

**Authors:** Ruixin Shao, Longfei Xin, Jun Mao, Leilei Li, Guozhang Kang, Qinghua Yang

**Affiliations:** 1 The Collaborative Innovation Center of Henan Grain Crops, Henan Agricultural University, Zhengzhou 450002, China; E-Mail: shao_rui_xin@126.com; 2 The National Key Laboratory of Wheat and Maize Crop Science, Henan Agricultural University, Zhengzhou 450002, China; E-Mails: cnxinlongfei@126.com (L.X.); mlsrmj3@163.com (J.M.); shaorx2012@163.com (L.L.)

**Keywords:** differentially expressed proteins, proteome, ultrastructure, water deficiency, *Zea mays* L.

## Abstract

After maize seedlings grown in full-strength Hoagland solution for 20 days were exposed to 20% polyethylene glycol (PEG)-stimulated water deficiency for two days, plant height, shoot fresh and dry weights, and pigment contents significantly decreased, whereas malondialdehyde (MDA) content greatly increased. Using transmission electron microscopy, we observed that chloroplasts of mesophyll cells in PEG-treated maize seedlings were swollen, with a disintegrating envelope and disrupted grana thylakoid lamellae. Using two-dimensional gel electrophoresis (2-DE) method, we were able to identify 22 protein spots with significantly altered abundance in the leaves of treated seedlings in response to water deficiency, 16 of which were successfully identified. These protein species were functionally classified into signal transduction, stress defense, carbohydrate metabolism, protein metabolism, and unknown categories. The change in the abundance of the identified protein species may be closely related to the phenotypic and physiological changes due to PEG-stimulated water deficiency. Most of the identified protein species were putatively located in chloroplasts, indicating that chloroplasts may be prone to damage by PEG stimulated-water deficiency in maize seedlings. Our results help clarify the molecular mechanisms of the responses of higher plants to severe water deficiency.

## 1. Introduction

Water deficiency is one of the most important environmental stresses limiting plant growth and crop yield [1]. In higher plants, the general effects of water deficiency on growth are fairly well known at the physiological level. For example, water deficiency progressively decreases CO_2_ assimilation, reduces pigment content and ribulose-1,5-bisphosphate carboxylase/oxygenase activity, increases the content of some osmolytes (e.g., proline, glycine betaine), and induces the generation of reactive oxygen species (ROS). Increased activity of antioxidative enzymes, such as superoxide dismutase (SOD), catalase (CAT), peroxidase (POD), and non-enzymatic compounds, such as ascorbate (ASA) and glutathione (GSH), help plants avoid and/or tolerate stress [2]. Although physiological responses to water deficiency have been intensively studied, a thorough molecular understanding of the responses in plants could help explore patterns of biological response and identify genes or proteins involved in tolerance to water deficiency [3].

The genetic bases of water deficiency response mechanisms have been studied using microarray transcript profiling, serial analyses of gene expression, and the identification of microRNAs; many candidate genes implicated in water deficiency and tolerance have been identified and reviewed [4]. Although analysis of gene expression at the mRNA level has enhanced our understanding of the responses of plants to water deficiency, the mRNA level is not always correlated well with the level of the corresponding protein, mainly due to the regulation of gene expression at the transcriptional, post-transcriptional, translational, and post-translational levels [5]. Because proteins are directly linked to cellular functions, proteomic analysis coupled with bioinformatics techniques can reveal complex biological functions involving large numbers of proteins (and networks thereof) and the metabolic pathways involved in cellular detoxification and abiotic tolerance mechanisms [6]. A few proteomic studies on water deficiency have already been reported in some higher plants, including sunflower [1], wheat [7], *Eucalyptus globulus* [8], and barley [9]. Almost all plant species show a degree of tolerance to water deficiency; however, its extent varies among different species and even among different tissues in a plant [2]. Therefore, proteomic studies of a variety of major crops are necessary to determine the molecular mechanism underlying plant resistance to water deficiency; such work will facilitate the development of crop cultivars that are better able to resist water deficiency [6].

Maize (*Zea mays* L.) is a globally important staple crop for food, livestock feed, and biofuels, whereas it is very sensitive to abiotic stresses, including water deficiency, which leads to an annual yield loss of 24 million tons [10]. In maize plants, water deficiency occurs most frequently at the seedling stage [10] and the few proteomic studies have been mainly performed at temporary water stress in this species [11,12]. To the best of our knowledge, there is no information on the leaves of maize seedlings under severe water deficiency. PEG have been used extensively for stable and controlled water deficiency in plants because it has a very low chronic toxicity, molecules with mol wt greater than 3000 are apparently not absorbed at all, and plant water relations can be similar whether the plants are growing in soil or in a PEG solution having an equal water potential [13]. In the present study, a comparative proteomic approach was conducted to identify physiological and proteomic changes in the leaf of maize seedlings suffering PEG-stimulated water deficiency. The possible implications of the identified protein species in response to water deficiency were further discussed. Results from this study could be helpful for further understanding the molecular mechanism of severe water deficiency in maize. Emphasis was placed on target proteins that may play crucial roles in protecting cellular metabolism from water deficiency.

## 2. Results and Discussion

### 2.1. Morphological, Physiological, and Ultrastructural Changes of Maize Seedlings under PEG-Stimulated Water Deficiency

**Figure 1:**
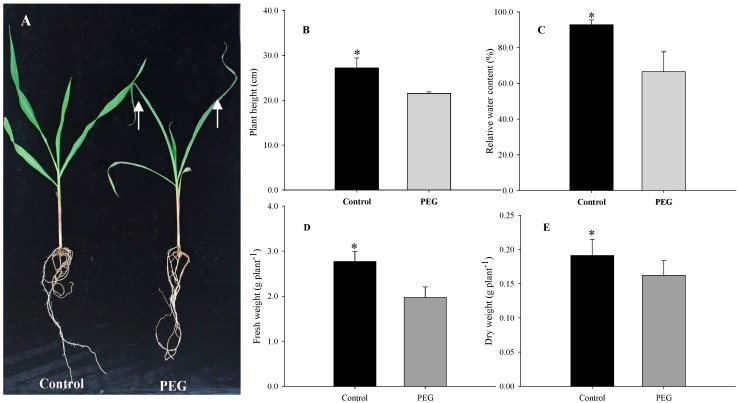
Phenotypes (**A**), plant height (**B**), relative water content of leaf (**C**), shoot fresh weights (**D**) and dry weights (**E**) of maize seedlings exposed to 20% PEG-stimulated water deficiency for two days. Arrowheads indicate the wilted leaves. Asterisks indicate significant differences between control and PEG-stressed maize seedlings at *p* < 0.05. Significant differences are determined by performing a one-way analysis of variance (ANOVA). Bars represent standard errors of triplicate experiments.

**Figure 2:**
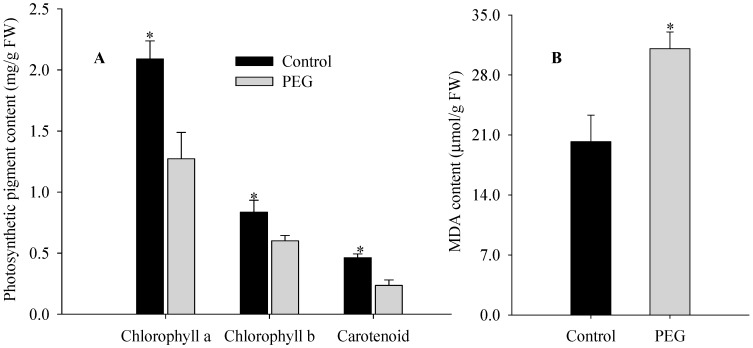
Contents of pigments (**A**) and MDA (**B**) in leaves of maize seedlings exposed to 20% PEG-stimulated water deficiency for two days. Asterisks indicate significant differences between control and PEG-stressed maize seedlings at *p* < 0.05. Significant differences are determined by performing a one-way analysis of variance (ANOVA). Bars represent standard errors of triplicate experiments.

Many plant species respond to water deficiency with changes in developmental, morphological, physiological, and biochemical processes, which may adversely affect growth, productivity, quality, and survival [2]. In the present study, maize seedlings exposed to 20% PEG-6000 for two days exhibited visible damage: tips of leaves became wilted and overall plant growth was greatly inhibited compared to control plants (Figure 1A). These qualitative phenotypic effects were confirmed by quantitative analysis. After two days of PEG-stimulated water deficiency, the seedlings showed significant decreases in plant height, relative water content (RWC) of leaf, fresh weight (FW) and dry weight (DW) of shoot compared to controls by 20.8%, 28.4%, 28.9%, and 15.1%, respectively (Figure 1B–E). In addition, the chlorophyll a, chlorophyll b, and carotenoid contents of leaves significantly decreased by 39.1%, 28.0% and 49.1%, respectively (Figure 2A), whereas MDA content increased significantly by 53.8% in treated plants (Figure 2B). These results suggest that 20% PEG-stimulated water deficiency is a severe abiotic stressor, greatly inhibiting the growth of maize seedlings. Our results are similar to the effects of water deficiency reported in a previous report on sunflower [1].

**Figure 3:**
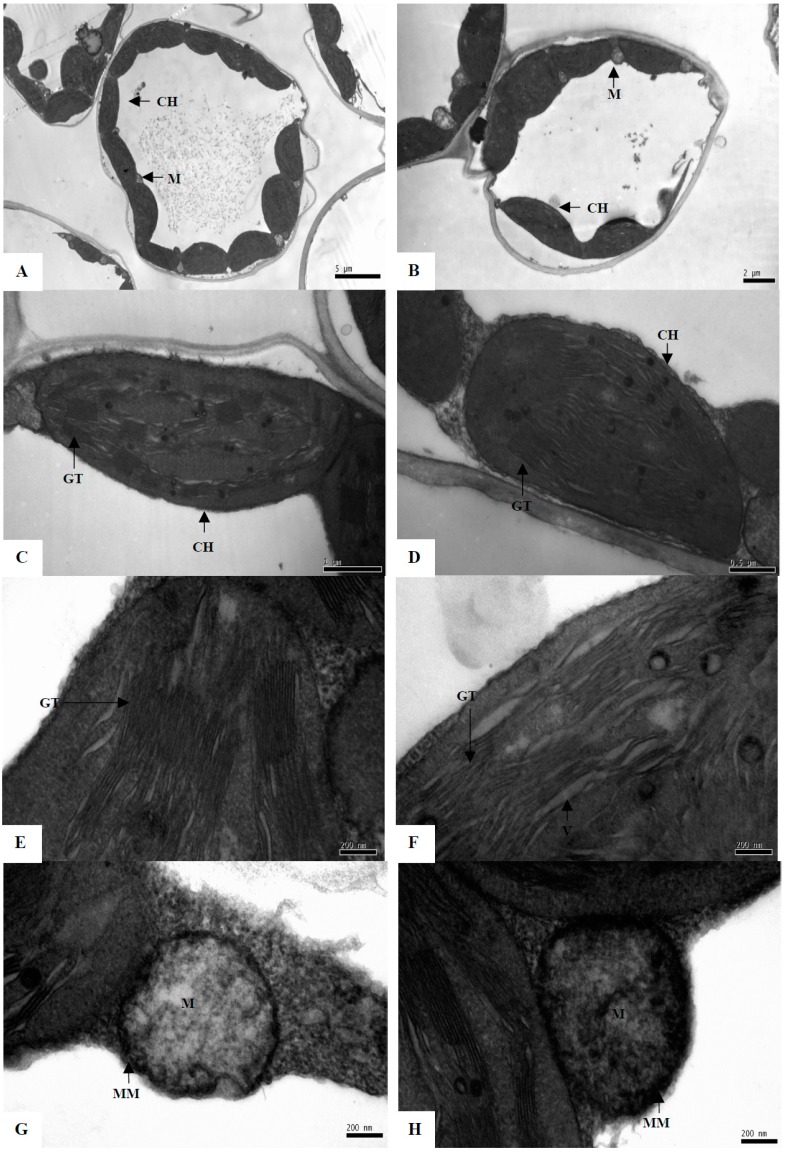
Transmission electron micrographs of the leaf sections of maize seedlings between control (control) (**A**,**C**,**E** and **G**) and 20% PEG-stimulated water deficiency treatment (**B**,**D**,**F** and **H**) for two days.

Chloroplasts are commonly the earliest visible site of ultrastructural changes due to abiotic stresses in plant cells [14]. We observed that, under normal growth conditions, chloroplasts of control plants were characterized by fully developed grana with numerous layers and well-developed stroma and thylakoid lamellae, and mitochondrial envelopes were also intact (Figure 3A,C,E,G). After water deficiency for two days, chloroplasts in the PEG treatment became swollen, with a disintegrating envelope and disrupted and irregularly shaped grana thylakoid lamellae (Figure 3B,D,F). Many large vesicles were present among the lamellae (Figure 3F). This indicates serious damage to chloroplast structures, consistent with a previous report on water deficiency-stressed tomato and sorghum plants [15]. However, no pronounced change was found in mitochondria, cell wall, cell membrane, *etc.*, in ultrastructures of maize seedlings under PEG-stimulated water deficiency (Figure 3H).

### 2.2. Leaf Proteome Analysis of Maize Seedlings Exposed to PEG-stimulated Water Deficiency

**Figure 4:**
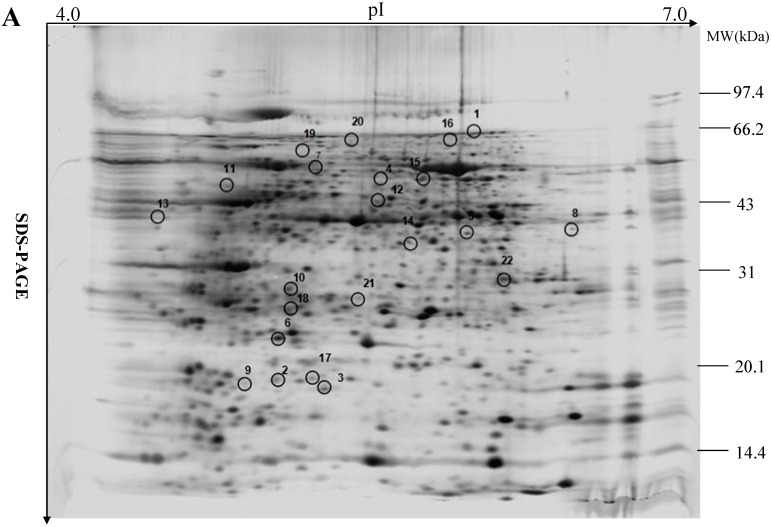

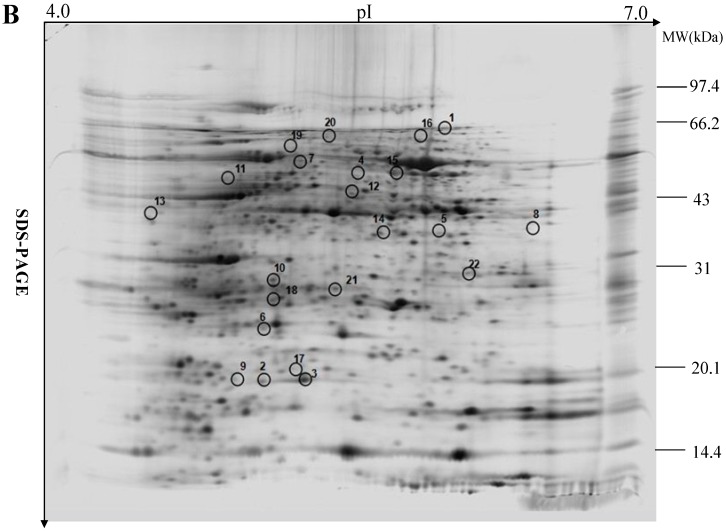
2-DE analysis of proteins extracted from control leaf sample (**A**) and PEG-stressed leaf sample (**B**) of maize seedlings.

To further elucidate the molecular mechanism of the response to water deficiency, the leaf proteomes of controls and PEG-treated plants were obtained using two-dimensional gel electrophoresis (2-DE). Representative 2-DE gels are shown in Figure 4 and all gels of the triplicate analysis are provided in Supplementary Figure S1. Peptide mass fingerprints (PMF) and MS/MS spectrum, the identified fragments per protein species, the sequence, score and other parameters of the identified protein species are indicated in Supplementary Files S1 and S2, and Supplementary Table S1, respectively. More than 800 spots from three sets of gels were quantified and 22 had significant changes in intensity (*p* < 0.05) of greater than 2.0-fold between PEG-treated and control seedlings (Figure 4). These 22 spots were analyzed by mass spectrometry and 16 spots were identified and analyzed by MS/MS using MALDI-TOF-TOF. The ratio is intensity of identified protein species in treated *vs.* control plants and is used to assess the changes in the abundance of the identified protein species between the groups. The functions of the 16 differentially expressed proteins were annotated based on BLASTp analysis in the NCBI database and the results are summarized in Table 1. They included β-d-glucan exohydrolase (spot 1), 17.4 kDa class I heat shock protein (spot 2), 16.9 kDa class I heat shock protein 1 (spot 3), WD40-like β propeller repeat family protein (spot 4), ribulose-1,5-bisphosphate carboxylase/oxygenase (spots 5, 6, and 16), natterin-4 (spot 7), electron carrier/electron transporter iron ion binding protein (spot 9), fumarylacetoacetate hydrolase domain-containing protein (spot 10), ribulose bisphosphate carboxylase/oxygenase activase (spot 11), translation elongation/initiation factor family protein (spot 12), fructose-bisphosphate aldolase (spot 13), RNA-binding protein FUS-like isoform X2 (spot 14), pore-forming toxin-like protein Hfr-2 (spot 15), and eukaryotic translation initiation factor 5A (spot 17).

Panels A and B, C and D, E and F, and G and H of the figure show single leaf cell, complete chloroplast, chloroplast internal structure and mitochondria of control and PEG treatment plants, respectively. Notes: CH, chloroplast; GT, grana thylakoid; M, mitochondria; MM, mitochondrion membrane; V, vesicle. Black lines express the bars of magnification.

Representative 2-DE gels of 800 μg total proteins from maize leaves were separated on 11 cm, pH 4–7 linear IPG strips followed by SDS-PAGE (12%). Positions and sizes of SDS-PAGE molecular weight markers (*M*_W_) are shown in kD.

**Table 1:** Differentially expressed protein species in leaves of maize seedlings exposed to 20% PEG-stimulated water deficiency for two days.

Spot No. ^a^	Protein Names (Species)	Accession No. ^b^	Protein PI/*M*w	Coverage (%)	Prot. Score /Ion Score ^c^	Pep. Count	*p*-Value	Ratio ^d^	Function ^e^	Subcellular Location ^f^
1	β-d-glucan exohydrolase (*Triticum aestivum*)	gi12227502	6.38/64.12	33	336/285	10	0.0062	−6.06	Carbohydrate metabolism	Other
2	17.4 kDa class I heat shock (*Zea mays*)	gi296512087	5.8/17.93	24	289/221	8	0.0021	+5.46	Stress defense	C
3	16.9 kDa class I heat shock protein 1 (*Zea mays*)	gi296512071	5.54/17.15	27	477/419	7	0.0017	+5.16	Stress defense	C
4	WD40-like β propeller repeat family protein (*Zea mays*)	gi413932479	5.76/74.24	24	454/367	15	0.0017	−4.98	Signal transduction	SP
5	Ribulose-1,5-bisphosphate carboxylase/oxygenase L-subunit (*Zea mays*)	gi11467200	6.33/53.29	26	489/405	14	0.0082	−4.90	Photosynthesis	C
6	Ribulose-1,5-bisphosphate carboxylase/oxygenase L-subunit (*Zea mays*)	gi11467200	9.77/21.62	35	365/259	6	0.0059	−3.96	Photosynthesis	C
7	Natterin-4 (*Triticum urartu*)	gi414587984	5.72/41.35	47	246/210	7	0.0042	+3.82	Unknown function	C
9	Electron carrier/electron transporter iron ion binding protein (*Zea mays*)	gi414886715	7.57/21.20	16	74/62	2	0.0014	+3.44	Signal transduction	C
10	Fumarylacetoacetate hydrolase domain-containing protein 1 (*Zea mays*)	gi226495395	5.75/24.74	11	308/275	5	0.0037	+3.32	Protein metabolism	SP
11	Ribulose bisphosphate carboxylase/oxygenase activase (*Zea mays*)	gi413920135	5.89/51.23	42	196/149	11	0.0023	−3.48	Photosynthesis	C
12	Translation elongation/initiation factor family protein (*Zea mays*)	gi414873962	6.11/48.68	45	498/383	15	0.0066	+3.28	Protein metabolism	M
13	Fructose-bisphosphate aldolase (*Zea mays*)	gi223975775	6.37/38.41	16	76/44	6	0.0087	+3.28	Carbohydrate metabolism	M
14	RNA-binding protein FUS-like isoform X2 (*Setaria italica*)	gi414876731	5.96/33.59	32	752/663	11	0.0017	+3.16	Signal transduction	Other
15	Pore-forming toxin-like protein Hfr-2 (*Triticum aestivum*)	gi414587991	6.53/42.29	49	959/789	19	0.0029	+3.08	Unknown function	C
16	Ribulose-1,5-bisphosphate carboxylase/oxygenase L-subunit (*Zea mays*)	gi11467200	6.33/53.29	31	100/259	16	0.0032	+3.04	Photosynthesis	C
17	Eukaryotic translation initiation factor 5A (*Zea mays*)	gi162464130	5.61/17.71	29	77/40	5	0.0024	−3.02	Protein metabolism	Other

^a^ Spot numbers correspond with 2-D gel as shown in Figure 4; ^b^ Accession number in NCBI database; ^c^ Protein score was based on combined MS and MS/MS spectra. The 16 proteins that had a statistically significant (*p* < 0.05) protein score were considered successfully identified; ^d^ Ratio: +, the increased abundance of identified protein species in PEG-stressed maize seedlings; −, the decreased abundance of identified protein species in PEG-stressed maize seedlings; ^e^ Function of proteins determined; ^f^ Putative cellular locations of the identified protein species. C, chloroplast; M, mitochondrion; Other, subcellular organs except for chloroplast, mitochondrion, and secretory pathway. The subcellular location is explained in web site: http://www.cbs.dtu.dk/services/TargetP-1.1/output.php.

The terminology of the identified protein species in this study was based on Schlüter and his colleagues [16]. Some spots were excised from the same gel and had the same names but different pI or *M*w. These spots should be divided into different protein species, because they could be different products of one gene or protein and may have different cellular tasks, due to the presence of nucleotide polymorphisms, alternative splicing, proteolytic cleavage, and post-translational modifications [17]. In this study, therefore, spots 5, 6, and 16 (ribulose-1,5-bisphosphate carboxylase/oxygenase l-subunit) should belong to different protein species.

The identified protein species functioned in signal transduction (spots 4, 9, and 14), stress defense (spots 2 and 3), photosynthesis (spots 5, 6, 11, and 16), protein metabolism (spots 10, 12, and 17), and carbohydrate metabolism (spots 1 and 13); spots 7 and 15 had unknown function (Table 1). The stress-responsive protein species involved in major biological processes are further discussed below.

Signal transduction: Under stress conditions, signal molecules play a crucial role in abiotic tolerance, because they can perceive stress signals and send them to the signal transduction machinery to regulate gene expression. Electron carrier/electron transporter iron ion binding protein is involved in biotic stress in higher plants [18], although its precise cellular function remains largely unknown. In our study, this protein species (spot 9) was markedly upregulated after water deficiency, indicating that it may also be a member of the signal transduction pathway in response to water deficiency in maize. RNA-binding proteins (RBPs) are recognized as key regulatory factors in the post-transcriptional regulation of gene expression in eukaryotes. Some RBPs act as RNA chaperones and are implicated to play roles in plant responses to diverse environmental stresses [19]. In the present study, the abundance of the RBP FUS-like isoform X2 (spot 14) was highly upregulated, indicating that it may be an RNA chaperone in the signal pathway of the water deficiency. WD40-like β propeller repeat family proteins are short, ~40 amino acid motifs, often terminating in a Trp-Asp (W-D) dipeptide, and are implicated in a variety of functions ranging from signal transduction and transcription regulation to development and stress [20]. Our proteomic data showed that the expression level of a WD40-like β propeller repeat family protein (spot 4) was dramatically decreased in leaves of treated seedlings, similar to a previous study on water deficiency-stressed rice plants [21]. This implies that this protein may act as a negative signal molecule in the water deficiency transduction pathway.

Stress defense: Heat shock proteins (HSPs) are a group of proteins whose expression is induced when cells are exposed to elevated temperatures and function as molecular chaperones that can stabilize chromatin structure, proteins, and membranes, and promote repair mechanisms through the refolding of proteins during and after exposure to stress. High levels of HSPs can also be triggered by exposure to different environmental stresses such as cold temperatures and salt [22]. The transcription levels of many HSP genes are significantly enhanced after exposure to water deficiency, and overexpression of HSPs enhances tolerance to various abiotic stresses [23]. In the present study, we observed the increased accumulation of two HSPs (spots 2 and 3) in the leaves of treated maize, indicating that they may protect maize seedlings from the damaging effects of water deficiency.

Photosynthesis: Downregulation of the photosynthetic capacity is a known response to abiotic stress. The effects of water deficiency on photosynthesis are either direct (as diffusion limitations through the stomata and mesophyll and changes in photosynthetic metabolism) or secondary, such as oxidative stress arising from the superimposition of multiple stresses [24]. The role of rubisco-1,5-bisphosphate carboxylase activase is to remove (via ATP hydrolysis under light conditions) inhibitory sugar phosphates, such as ribulose-1,5-biphosphate, from the active sites of Rubisco, so that CO_2_ can activate the enzyme by carbamylation [25]. Previous studies have focused on changes in Rubisco content, activity, and mRNA level under water deficiency conditions [26]. Our data indicate that PEG-stimulated water deficiency greatly inhibited the abundance of many Rubisco protein species (spots 5, 6, and 11), implying that photosynthesis may be inhibited in water deficiency-stressed maize seedlings. This may account for the decreased content of photosynthetic pigments (Figure 2A) and the damaged chloroplast ultrastructures that we observed (Figure 3).

Protein metabolism: Under water deficiency and other environmental stresses, protein content obviously decreases, possibly because elevated oxidative damage causes protein degradation [27]. In the present study, the expression levels of fumarylacetoacetate hydrolase domain-containing protein 1 (spot 10) and a translation elongation/initiation factor family protein (spot 12) were greatly upregulated in leaves of treated seedlings, whereas the abundance of translation initiation factor 5A (spot 17) was dramatically downregulated (Table 1). Fumarylacetoacetate hydrolase domain-containing protein hydrolyzes fumarylacetoacetate into fumarate and acetoacetate, the final step in the tyrosine (Tyr) degradation pathway, which is essential in animals. A deficiency of fumarylacetoacetate hydrolase in animals results in a lethal congenital disorder. However, its role in the Tyr degradation pathway in plants remains to be elucidated. A recent study reported that fumarylacetoacetate hydrolase domain-containing protein is involved in cell death in *Arabidopsis* [28]. Translation initiation factors are normally associated with ribosomes and play important roles in protein biosynthesis [29]. In the present study, these protein species were diversely regulated in the leaves of water deficiency-stressed seedlings, suggesting that protein metabolism may be greatly modified, as indicated by the changed proteome profiles between the control and water deficiency-stressed seedlings (Figure 4).

Carbohydrate metabolism: Carbohydrate metabolism regulates sugar synthesis and transformation, as well as carbon partitioning, and water deficiency disrupts carbohydrate metabolism in plants [30]. β-d-glucan exohydrolase hydrolyzes (1,3)-β-glucan (*i.e*., laminarin) but also hydrolyzes (1,3 or 1,4)-β-glucan and 4-nitrophenyl β-d-glucoside, which are used in the generation of sugars from the lignocellulosic biomass [31]. The greatly inhibited abundance of this protein species (spot 1) may account for the stunted growth of maize seedlings exposed to water deficiency in this study (Figure 1). Fructose-bisphosphate aldolase is a key enzyme of the glycolytic pathway and is responsible for the reversible cleavage of fructose 1,6-bisphosphate to glyceraldehyde 3-phosphate (G3P) and dihydroxyacetone phosphate. This enzyme also catalyzes the segmentation of structurally related sugar phosphates, including fructose 1-phosphate, an intermediate of fructose metabolism [32]. The abundance of a fructose-bisphosphate aldolase (spot 13) was significantly enhanced in leaves of stressed seedlings, implying that more fructose may be produced.

Unknown function: Pore-forming toxins are the most common bacterial cytotoxic proteins and are required for virulence in a large number of important pathogens. Natterin-like protein is a pore-forming toxin with an unusually high toxicity and may be involved in the defense mechanism against specific predators in animals [33]. The functions of these two types of proteins have remained unclear in higher plants, but their transcription levels were greatly increased under biotic stress [34]. Our proteomic results showed that the abundances of natterin-4 (spot 7) and pore-forming toxin-like protein Hfr-2 (spot 15) were obviously enhanced in leaves of stressed plants, indicating that these two protein species may function in the response to both biotic and water deficiency, possibly through shared signaling pathways.

### 2.3. Subcellular Location of the Identified Protein Species

The subcellular localizations of the identified protein species were analyzed using the TargetP program (www.cbs.dtu.dk/services/TargetP) [35]. Most (9/16) of the identified protein species related to the stress response were localized to the chloroplasts (Table 1), similar to previous studies on ultrastructural changes in plants in response to many abiotic stresses including cold and salt [15]. This suggests that chloroplasts may be prone to damage by water deficiency in maize seedlings (Figure 3).

### 2.4. Comparison between mRNA and Protein Levels of PEG Stress-Responsive Protein Species

**Figure 5:**
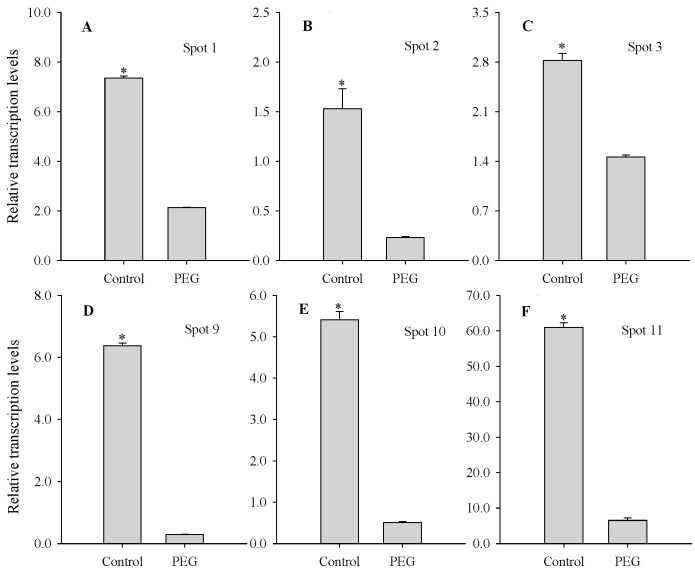
Transcription levels of the genes encoding six water deficiency-responsive protein species in leaves of maize seedlings exposed to PEG-stimulated water deficiency for two days. Asterisks indicate significant differences between control and PEG-stressed maize seedlings at *p* < 0.05. Bars represented standard errors of triplicate experiments. Fold change in mRNA level was determined using the 2^−ΔΔ*C*t^ method. Spot 1 (**A**); β-d-glucan exohydrolase; Spot 2 (**B**); 17.4 kDa class I heat shock; Spot 3 (**C**); 16.9 kDa class I heat shock protein 1; Spot 9 (**D**); electron carrier/electron transporter iron ion binding protein; Spot 10 (**E**); fumarylacetoacetate hydrolase domain-containing protein 1; Spot 11 (**F**), ribulose bisphosphate carboxylase/oxygenase activase.

Four upregulated protein species (spots 2, 3, 9 and 10) and two of the stress-responsive downregulated protein species (spots 1 and 11) were chosen for further analysis as examples of the observed variation in the patterns of spot intensity. To investigate the changes in gene expression at the mRNA level, quantitative real-time PCR (qPCR) was performed for the genes encoding these protein species. The transcription levels of the genes encoding spots 1 and 11 approximately coincided with their expression levels (Figure 5A,F). However, the transcription levels of the genes encoding the other four protein species (spots 2, 3, 9 and 10) differed from their protein levels (Figure 5B–E). These suggested that the mRNA level did not always correlate well with the protein level, possibly due to post-transcriptional regulatory mechanisms, such as nuclear export, mRNA localization, transcript stability, translational regulation, and protein degradation [17].

### 2.5. Comparisons of Proteome Expression Patterns between this Study and Others

Two previous studies reported the 2-DE proteome profiles of the root and leaf tissues of maize plants in response to temporary moderate water deficiency, respectively [11,12]. Here, we compare our results to these two studies. We have found that there are no common identified protein species between them. It has been speculated that this can mainly be due to distinct mechanisms between moderate water deficiency and severe water deficiency [36]. This study could put forward an innovative molecular mechanism of severe water deficiency in maize.

### 2.6. Putative Mechanism of Water Deficiency in Higher Plants

**Figure 6:**
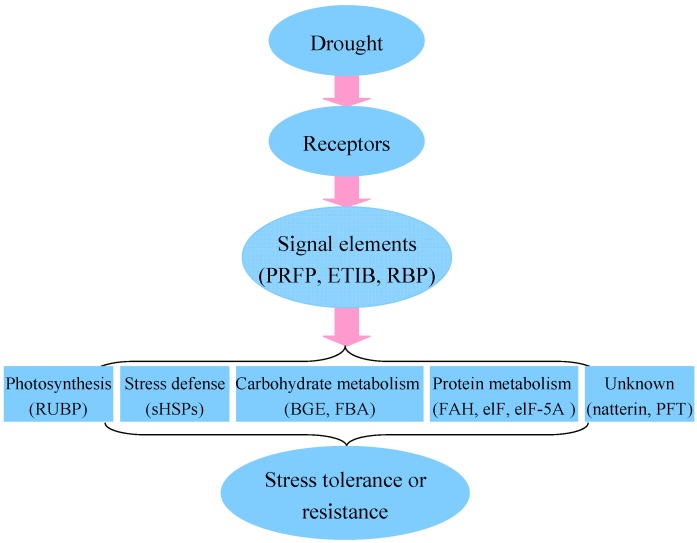
Schematic model of the action of water deficiency stress modified from Huang and colleagues [27]. The figure between brackets indicated the regulated protein species identified in the present study. BGE, β-d-glucan exohydrolase; eIF, translation elongation/initiation factor family protein; eIF-5A, eukaryotic translation initiation factor 5A; ETIB, electron carrier/electron transporter iron ion binding protein; FAH, fumarylacetoacetate hydrolase domain-containing protein 1; FBA, fructose-bisphosphate aldolase; sHSPs, small heat shock proteins include 16.9 and 17.4 kDa; PFT, pore-forming toxin-like protein Hfr-2; PRFP, WD40-like beta propeller repeat family protein; RBP, RNA-binding protein FUS-like isoform X2; RUBP, ribulose-1,5-bisphosphate carboxylase/oxygenase or ribulose bisphosphate carboxylase/oxygenase activase.

Huang and his colleagues proposed one potential pathway for the water deficiency response of higher plants [27]. In this pathway, it has been found that water deficiency signal transduction pathway starts with signal perception, is followed by the generation of second messengers including calcium, and then the second messengers further interact with their respective interacting partners initiating a phosphorylation cascade and target the major stress responsive genes or the transcription. In the present study, the abundance of many protein species involved in this proposed pathway was altered under water deficiency (Figure 6), suggesting that these proteins function in response to water deficiency.

## 3. Experimental Section

### 3.1. Plant Materials

Seeds of the maize (*Zea mays* L.) inbred line Wi6-2 were soaked in water containing 0.2% (*w*/*v*) tiuram and benomyl for 24 h to prevent fungal infection, then placed on moist gauze spread on disks and finally kept in darkness to germinate at 28 °C for 4 days. Uniformly germinated seeds were grown in pots and cultured with full-strength Hoagland’s nutrient solution [37] in a growth chamber (LT/ACR-2002, artificial climate chamber control system, Yishengtaihe Company, Beijing, China) under a 13-h photoperiod, 28/22 °C day/night temperatures, light intensity of 600 μmol·m^−2^·s^−1^, and relative humidity of 60/75% (day/night).

### 3.2. PEG-Stimulated Water Deficiency

After the seedlings were grown for 20 days, 20% PEG (−0.8 MPa osmotic potential, PEG-6000) (Sigma Chemical Co., St. Louis, MO, USA) was added to full-strength Hoagland solution to induce water deficiency for 2 days [38]. PEG solution was replaced daily during the stress period. A parallel set of maize seedlings was maintained in full-strength Hoagland’s solution without PEG for 2 days as a control. After 2 days of PEG stress, the uppermost fully developed leaves of the PEG-treated and untreated (control) plants were collected, immediately frozen in liquid nitrogen and stored at −80 °C until further analysis.

### 3.3. Growth Parameters

Growth parameters, such as the height and fresh weight (FW) of plants, were determined immediately following 2 days of water deficiency. Dry weight (DW) of plants were determined after the plant samples were placed in an oven at 105 °C for 15 min and then dried to a constant weight at 75 °C. Relative water content (RWC) of leaves was recorded according to the method of Barrs and Weatherley [39]. Three independent biological replicates with nine plants each replication were performed.

### 3.4. Transmission Electron Microscopy

After exposure to PEG stress for 2 days, leaves were randomly selected for cell ultrastructural observation. Sample sections from the middle portion of the uppermost fully developed leaf (1 mm^2^, middle section of the fully expanded leaf) were excised and fixed in cold 4% (*v*/*v*) glutaraldehyde in 0.1 M potassium phosphate buffer (PBS, pH 7.2), vacuum-infiltrated until the material sank, and left overnight at 4 °C. Then the samples were washed five times in 0.1 M PBS buffer (20 min each) and post-fixed in 1% osmium tetroxide (OsO_4_, osmium [VIII] oxide) for 16 h at 4 °C in 0.1 M PBS buffer. The materials were washed five times in 0.1 M PBS buffer, dehydrated in a graded ethanol series (10%, 20%, 30%, 40%, 50%, 60%, 70%, 80%, 90%, 95% and 100%) at 15–20 min intervals, followed by acetone (100%) for 20 min, and then infiltrated and embedded in Spurr’s resin overnight. Ultra-thin sections ~80–90 nm were obtained with a diamond knife on an EM US6 ultramicrotome (Leica, Wetzlar, Germany) and then stained with 2% uranyl acetate for 90 min and 6% lead citrate for 10 min. Sample semimicrosections were made with a LKB-V ultramicrotome (LKB, Bromma, Sweden) at 90 kV. At least five sections from each treatment were examined.

### 3.5. Measurement of Photosynthetic Pigments and MDA Content

Photosynthetic pigments were extracted in 95% ethanol in darkness for 72 h and calculated according to the method of Lichtenthaler [40]. The supernatants in 80% (*v*/*v*) acetone were collected and read at 665, 649, and 470 nm to quantify chlorophyll a, chlorophyll b, and carotenoids, respectively. MDA content was determined and calculated from UV absorbance at 600, 532, and 450 nm as described previously [41]. The absorbance of the supernatants was measured with a UNICO (UV-2600, UNICO Instruments Co., Ltd., Shanghai, China) spectrophotometer.

### 3.6. Protein Extraction

Proteins from leaf samples were extracted using the trichloroacetic acid (TCA)/acetone method, and three independent biological replicates were performed [42]. Leaf samples (1.5 g) were ground to powder in liquid nitrogen with a mortar and pestle. Next, 10 volumes of ice-cold 10% (*w*/*v*) TCA in acetone with 0.07% 2-mercaptoethanol were added, followed by incubation at −20 °C for 2 h. After centrifugation at 14,000× *g* for 30 min at 4 °C, the supernatants were discarded and the pellets were washed three times with ice-cold acetone containing 0.07% 2-mercaptoethanol. The final pellet was vacuum-dried and resuspended in lysis buffer consisting of 8 M urea, 2 M thiourea, 4% (*w*/*v*) 3-[(3-cholamidopropyl) dimethylammonio]-1-propanesulfonate (CHAPS), 0.5% (*v*/*v*) immobilized pH gradient (IPG) buffer (pH 4–7), and 1 mM fresh dithiothreitol (DTT) and incubated at 25 °C for 1–2 h. The suspension was centrifuged at 14,000× *g* for 30 min at 25 °C to remove the insoluble materials. Supernatants were collected and used as protein extracts. Protein concentrations were determined using the Bradford protein assay (Bio-Rad, Hercules, CA, US) with a UV-2600 spectrophotometer (Shimadzu Co., Japan) at an absorbance of 595 nm with a bovine serum albumin (BSA) standard.

### 3.7. 2-DE Separation and Image Analysis

Protein samples (800 μg) were loaded onto an Immobiline DryStrip (GE Healthcare, 11 cm, pH 4–7) and rehydrated passively for 10–12 h at 20 °C and 12 h at 30 V using an Ettan™ IPGphor II™ (GE Healthcare, Munich, Germany). Isoelectric focusing was conducted for a total of approximately 60 kVh with the following steps: 500 V for 1 h, 1000 V for 1 h, 8000 V for 8 h, and 500 V for 4 h. After isoelectric focusing, disulfide bonds were reduced by placing the IPG strips in equilibration buffer I for 15 min (6 M urea, 2% SDS, 50 mM Tris-HCl, pH 8.8, 30% glycerol, 0.002% Bromophenol blue, 10 mg/mL DTT). Then the strips were incubated for 15 min in equilibration buffer II (6 M urea, 2% SDS, 50 mM Tris-HCl, pH 8.8, 30% glycerol, 0.002% Bromophenol blue, 400 mg iodoacetamide). For the second dimension, the IPG strips were placed on 12.5% polyacrylamide gels in an Ettan Dalt 6 (GE Healthcare) electrophoresis system at 20 °C. Electrophoresis parameters were 15 mA/gel for 30 min and then 30 mA/gel. The reaction was stopped after approximately 6 h. A running buffer of 25 mM Tris, pH 8.3, 192 mM glycine, and 0.1% SDS was used.

Protein spot images were visualized using Coomassie Brilliant Blue (CBB) and scanned at 300 dpi with a UMAX Power Look 2, 100 XL scanner (Maximum Tech Inc., Taiwan). Image analysis was performed with PDQuest software version 8.0 (Bio-Rad, Hercules, CA, USA). The normalized percentage volumes of protein spots from triplicate biological samples were subjected to statistical analysis by SPSS (version 17.0, IBM, Chicago, IL, USA). Quantitative image analysis was conducted to reveal protein spots with reproducible and significant differences in abundance (>2.0-fold and *p*-value < 0.05).

### 3.8. In-Gel Digestion and MALDI-TOF-TOF MS/MS Analysis

Selected protein spots were manually excised from gels and digested with sequencing grade trypsin. Gel slices were destained with 100 mM NH_4_HCO_3_ in 30% acetonitrile (ACN) and lyophilized for 20 min prior to digestion at 37 °C overnight (20 h) with 20 μL 50 mM NH_4_HCO_3_ containing 0.01 mg/mL sequencing grade modified trypsin (Promega, Madison, WI, USA). After brief centrifugation, peptides were collected from the supernatant and the remaining gel pieces were further sonicated for 15 min in 100 μL 60% CAN/0.1% TFA to collect the remaining peptides. The peptides from one protein spot were combined.

Samples were analyzed using MALDI-TOF/TOF with a proteomics analyzer (4800 P; Applied Biosystems, Foster City, CA, USA). Mono-isotopic peak masses were acquired in a mass range of 800 to 4000 Da and noise ratio (S/N) of 50, and up to eight of the most intense ions were selected as precursors for MS/MS acquisition. In MS/MS-positive ion mode, spectra were averaged and collision energy was set to 2 kV. The peptide mass fingerprint combined MS/MS data were submitted to Mascot version 2.1 (Matrix Science, London, UK) for identification according to the NCBI database (900091 release). The search variables were set as follows: Viridiplantae, trypsin cleavage (one missed cleavage allowed), carbamidomethylation as fixed modification, methionine oxidation as the variable modification, peptide mass tolerance set at 100 ppm and fragment mass tolerance set between 0.1–0.2 Da. Proteins with a protein score confidence interval percentage (CI%) and a total ion score CI% above 95% were considered credible results for MS/MS.

### 3.9. qPCR

Total RNA was extracted from leaves using TRIzol^®^ RNA Isolation Reagent (Invitrogen, Carlsbad, CA, USA) and treated with RNase-free DNase I (Takara Biotechnology (Dalian) Co., Ltd., Dalian, China) to remove contaminating genomic DNA. The integrity of the RNA samples was confirmed by gel electrophoresis. cDNA was prepared from 2 µg total RNA with ThermoScript Reverse Transcriptase (Invitrogen) and an oligo-dT_18_ primer at a temperature of 42 °C according to the manufacturer’s instructions. qPCR was used to measure the transcription levels of the target genes. qPCR was performed using an SYBR Premix Ex Taq (Perfect Real Time) Kit (Takara Biotechnology (Dalian) Co., Ltd.) in a LightCycler^®^ 480 Real-Time PCR System (Roche Diagnostics Ltd., West Sussex, UK) according to the manufacturer’s instructions. Each reaction (20 µL) consisted of 10 µL SYBR Green Supermix (2×), 1 µL diluted cDNA, and 0.5 µL forward and reverse primers. The relative transcript levels were calculated using the 2^−ΔΔ*C*t^ method with the maize β-actin gene (GenBank Accession No. J01238) as an internal control. The primers used for qPCR are shown in Table 2 and were designed based on maize cDNA sequences encoding candidate proteins available in the NCBI BLAST database (www.ncbi.nlm.nih.gov/BLAST/).

**Table 2:** Primer sequences used in quantitative real-time polymerase chain reaction.

Spot No.	Accession No. of Protein Species	Accession No. of the Related Genes	Forward Primer	Reverse Primer	Product Lengths (bp)
1	gi12227501	AX053136	GTGGAACGCATAACGGAATC	GTGGAGACACCTCGGATGAT	109
2	gi296512087	CBM39185	GGAGGAGAAGAGGGACACCT	ATGGACGCACTGATCTGCT	108
3	gi296512071	CBM39177	ATCCCTTCGACACCATGTTC	CCTTGACCTCCTCCTTCTTG	159
9	gi414886715	NM_001147896	GAAGGCAAGGAAATGCTGTC	TATCTTCCACTCCGGCAACT	153
10	gi226495395	NP_001168488	TCGTCACCAGCATCATCATC	TGCTCCGTCTCCTTGTTCAC	109
11	gi413920135	NC_001666	CTACGCGGTGGACTTGATTT	CAGTTTCGGCTTGTGCTTTA	121
β-*actin*	gi168403	J01238	GTTTCCTGGGATTGCCGAT	CTGCTGAAAAGTGCTGAG	130

The numbers of protein spots are shown in Figure 4. The related names of protein species are shown in Table 2, and the accession numbers of the related genes are identified in NCBI database according to their protein accession numbers.

### 3.10. Statistical Analysis

All experiments were repeated independently three times. Spot intensities of differentially expressed proteins in a 2D gel were calculated from three spots in three replicate gels. Growth and physiological parameters, as well as spot intensities, were statistically analyzed using one-way analysis of variance (ANOVA) and Duncan’s multiple range test to determine significant differences among group means. Significant differences from control values were determined at the *p* < 0.05 level.

## 4. Conclusions

A severe water deficiency was induced by 20% PEG-stimulated stress, which greatly inhibited the growth of maize seedlings. Using 2-DE and MALDI-TOF/TOF MS, 16 differentially expressed protein species were identified in the leaves of stressed plants, and they are involved in signal transduction, stress defense, protein metabolism, photosynthesis, carbohydrate metabolism, and unknown function. The changes in the abundances of the identified protein species might be functionally relevant to many biological processes in maize seedlings suffering from severe water deficiency. The identified protein species may be closely related to the phenotypic and physiological changes of PEG-stressed maize seedlings. This study provides new, comprehensive insight into the molecular mechanisms of the severe water deficiency responses in higher plants.

## Supplementary Materials

Supplementary materials can be found at http://www.mdpi.com/1422-0067/16/09/21606/s1.
