# Supplementary material for: Physiological, Ultrastructural and Proteomic Responses in the Leaf of Maize Seedlings to Polyethylene Glycol-Stimulated Severe Water Deficiency
Source: Int J Mol Sci. 2015 Sep 8;16(9):21606–25. doi: 10.3390/ijms160921606 (PMC4613270; doi:10.3390/ijms160921606)
Supplement: Supplementary file 1 [file ijms-16-21606-s001.zip › ijms-96220-Supplementary Information/Supplementary Figure.pdf]

Supplementary Information

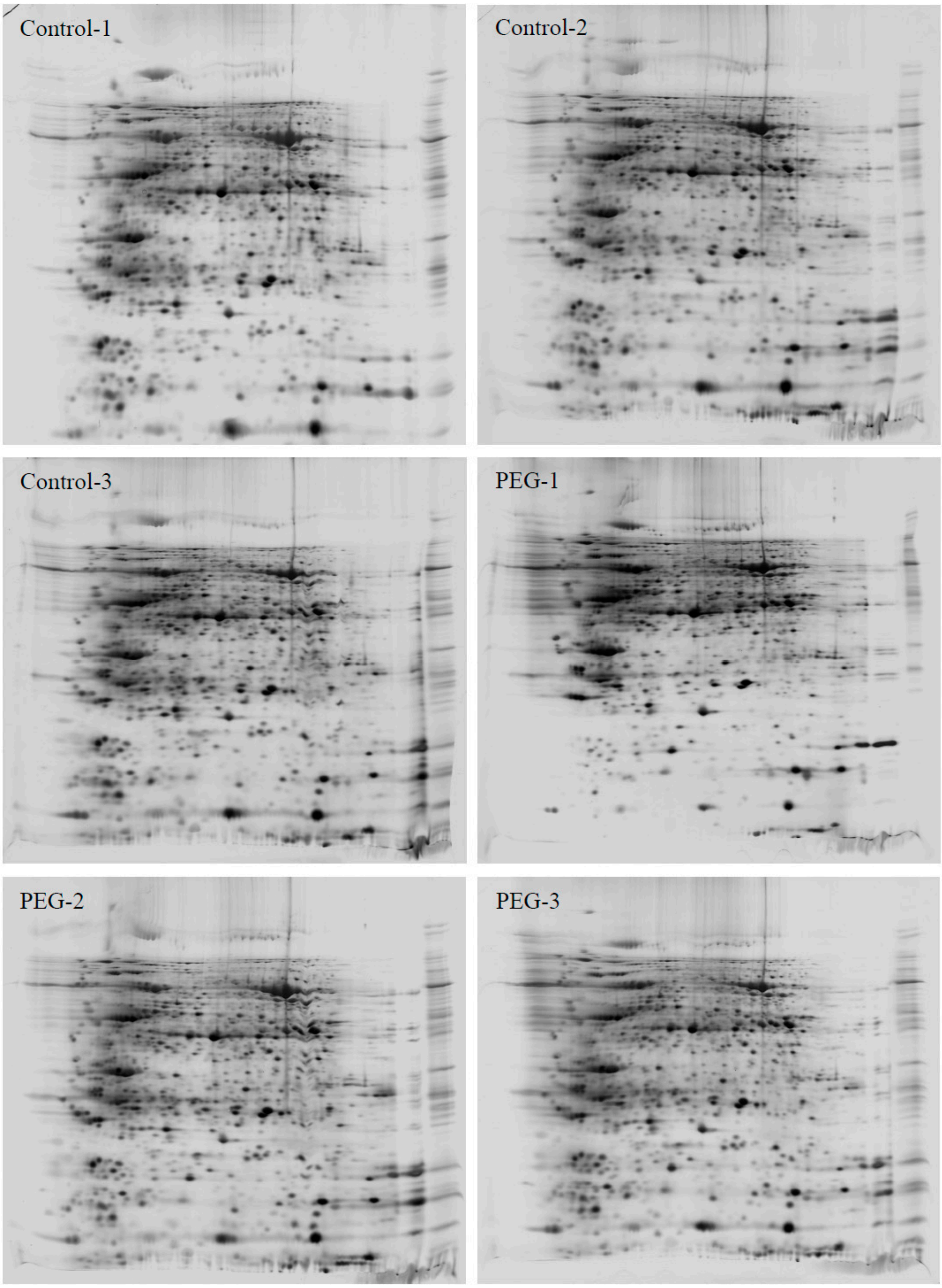

Figure S1. Triplicate biological 2-DE gels on the maize leaf proteomes of control and PEG-stimulated water deficiency treatments.
