# Supplementary material for: Physiological, Ultrastructural and Proteomic Responses in the Leaf of Maize Seedlings to Polyethylene Glycol-Stimulated Severe Water Deficiency
Source: Int J Mol Sci. 2015 Sep 8;16(9):21606–25. doi: 10.3390/ijms160921606 (PMC4613270; doi:10.3390/ijms160921606)
Supplement: Supplementary file 1 [file ijms-16-21606-s001.zip › ijms-96220-Supplementary Information/Supplementary File S1/MS-PDF/spot 11-C5.pdf]

Applied Biosystems 4700 Proteomics Analyzer 7044

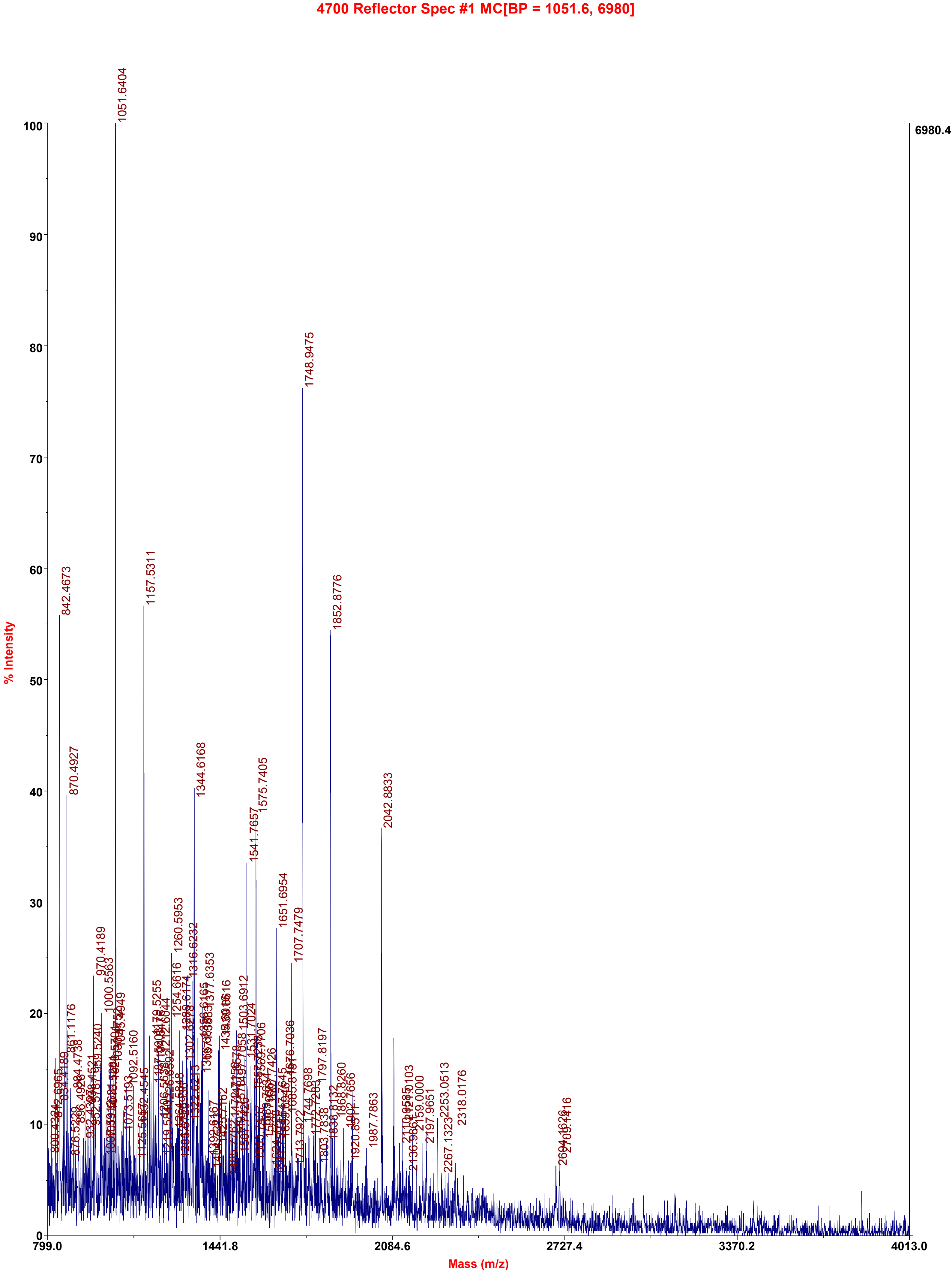

Printed: 13:00, March 31, 2014

E:\试验数据\2013.5\2D-蛋白\正式实验\P13118项目补充的内容(1)\P13118 spectrumraw\P13118 spectrumraw\MS\Sample test\Sample Project 20130720\C5_MS_1.t2d
