# Supplementary material for: Physiological, Ultrastructural and Proteomic Responses in the Leaf of Maize Seedlings to Polyethylene Glycol-Stimulated Severe Water Deficiency
Source: Int J Mol Sci. 2015 Sep 8;16(9):21606–25. doi: 10.3390/ijms160921606 (PMC4613270; doi:10.3390/ijms160921606)
Supplement: Supplementary file 1 [file ijms-16-21606-s001.zip › ijms-96220-Supplementary Information/Supplementary File S2/MSMS-PDF/spot 1-C2.pdf]

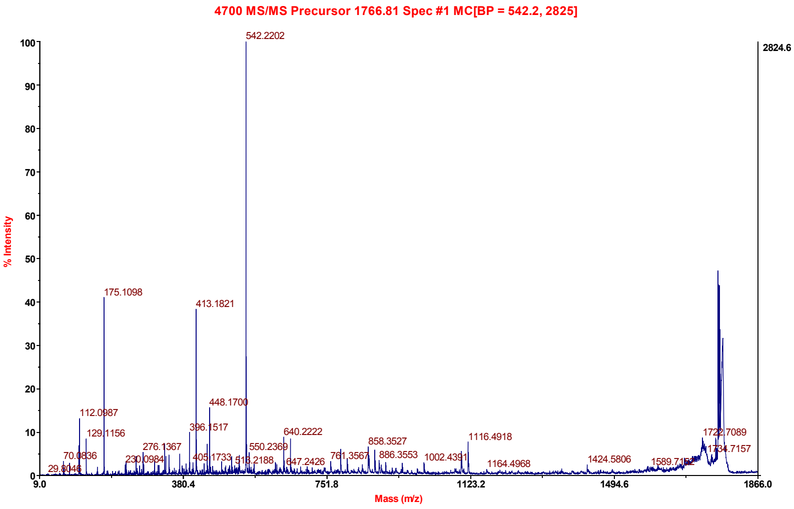

E:\...\C2_MSMS_1766.8110_3.t2d
Acquired:

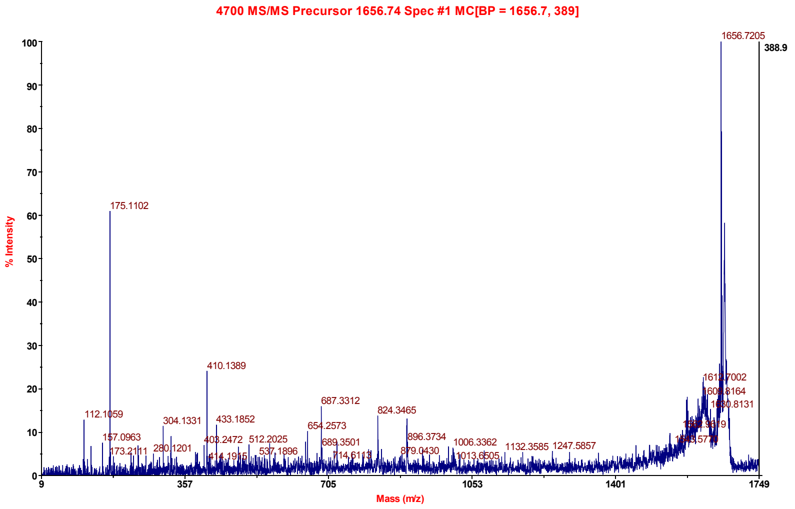

E:\...\C2_MSMS_1656.7396_10.t2d
Acquired:

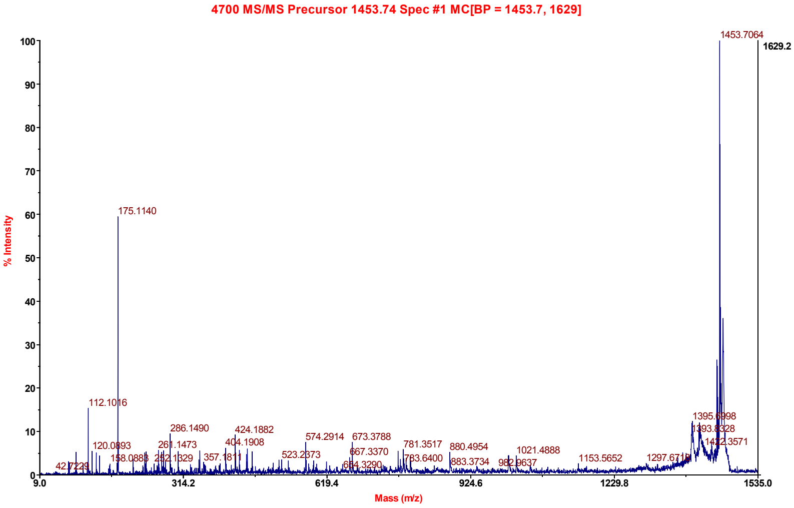

E:\...\C2_MSMS_1453.7362_4.t2d
Acquired:

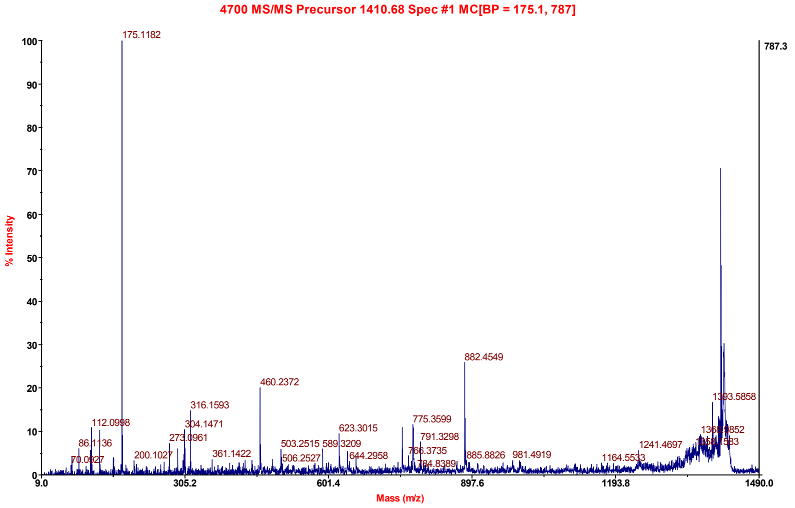

E:\...\C2_MSMS_1410.6760_11.t2d
Acquired:

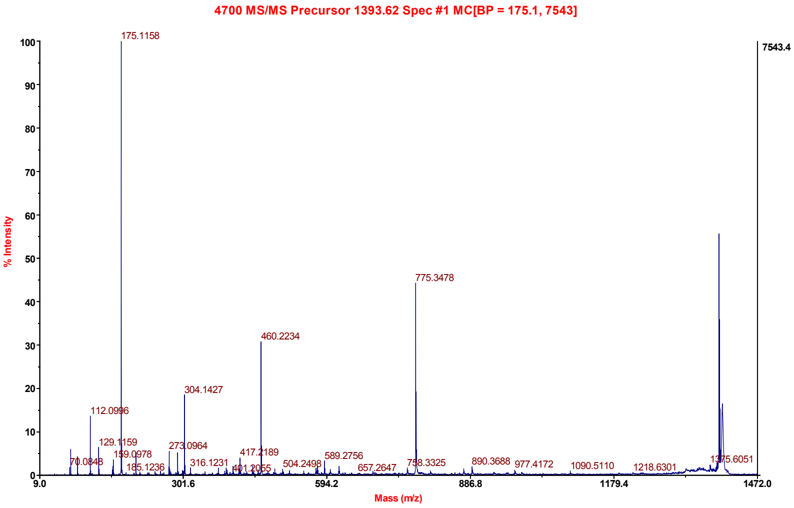

E:\...\C2_MSMS_1393.6205_2.t2d
Acquired:

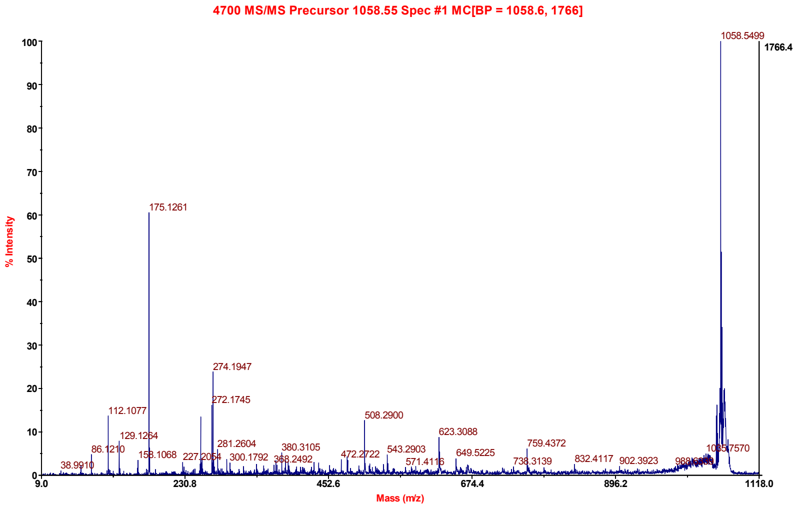

E:\...\C2_MSMS_1058.5481_7.t2d
Acquired:

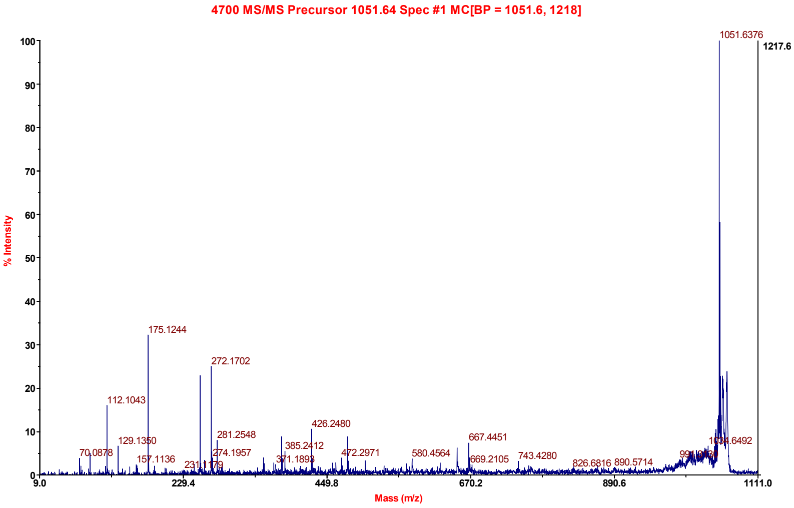

E:\...\C2_MSMS_1051.6426_9.t2d
Acquired:

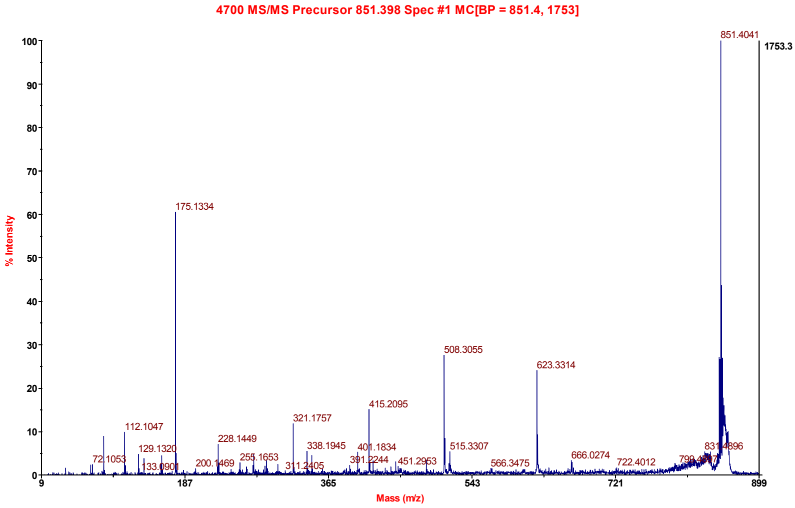

E:\...\C2_MSMS_851.3978_5.t2d
Acquired:
