# Supplementary material for: Physiological, Ultrastructural and Proteomic Responses in the Leaf of Maize Seedlings to Polyethylene Glycol-Stimulated Severe Water Deficiency
Source: Int J Mol Sci. 2015 Sep 8;16(9):21606–25. doi: 10.3390/ijms160921606 (PMC4613270; doi:10.3390/ijms160921606)
Supplement: Supplementary file 1 [file ijms-16-21606-s001.zip › ijms-96220-Supplementary Information/Supplementary File S2/MSMS-PDF/spot 10-C12.pdf]

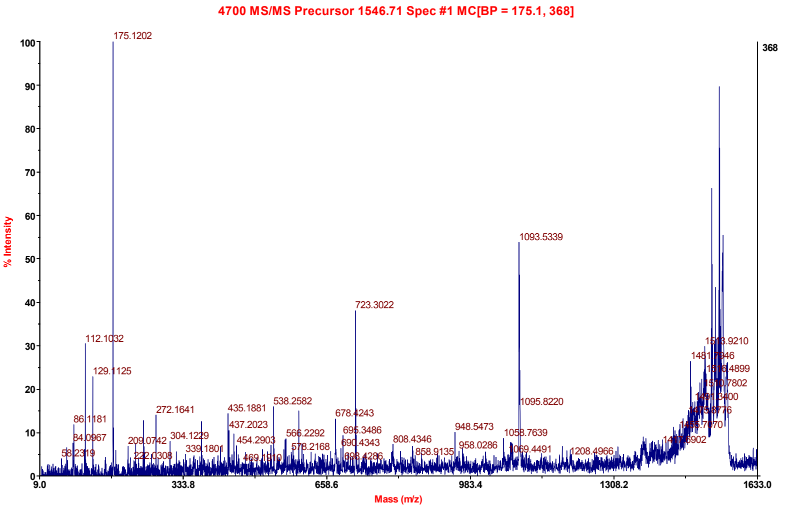

E:\...\C12_MSMS_1546.7107_10.t2d
Acquired:

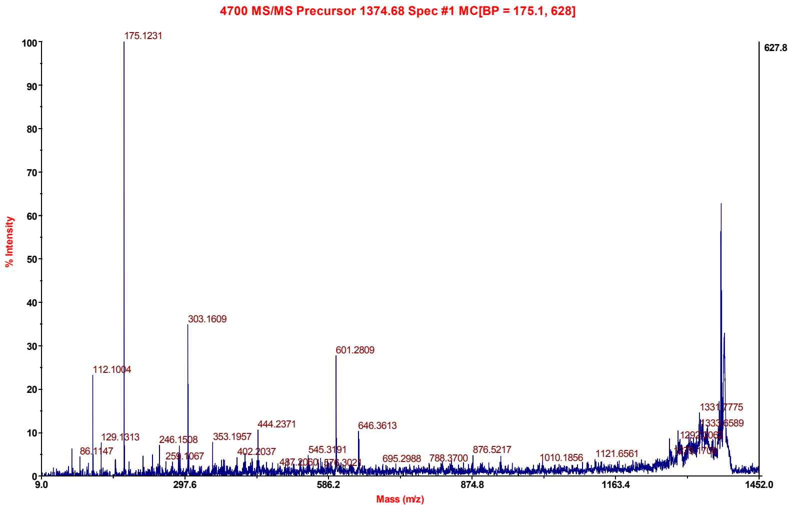

E:\...\C12_MSMS_1374.6779_11.t2d
Acquired:

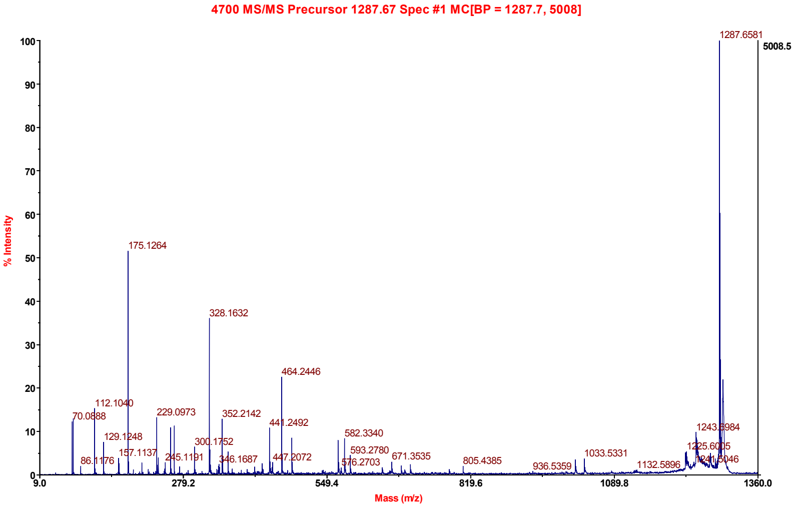

E:\...\C12_MSMS_1287.6691_2.t2d
Acquired:

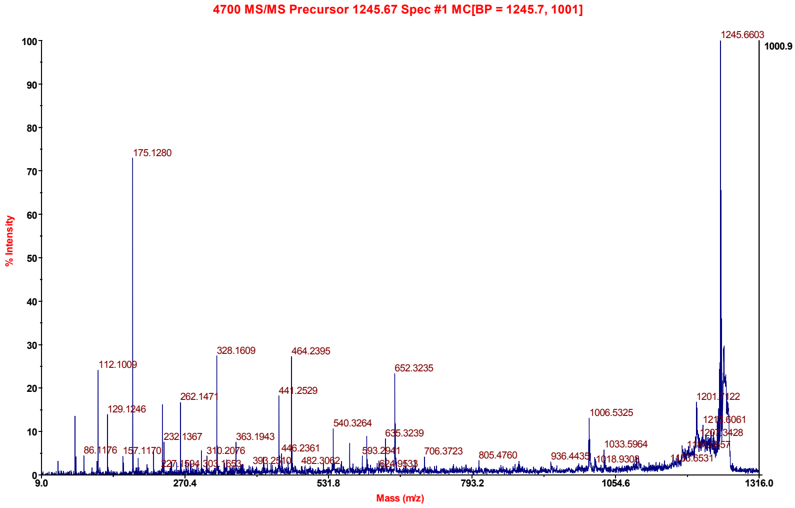

E:\...\C12_MSMS_1245.6653_7.t2d
Acquired:

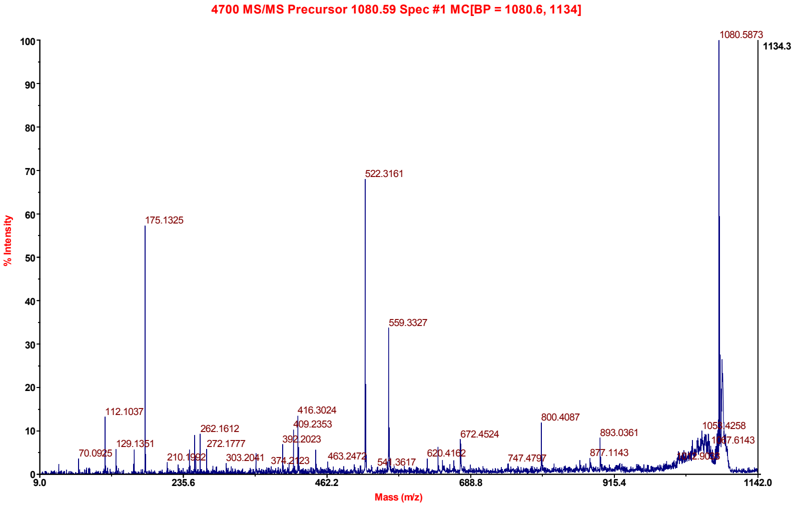

E:\...\C12_MSMS_1080.5919_9.t2d
Acquired:

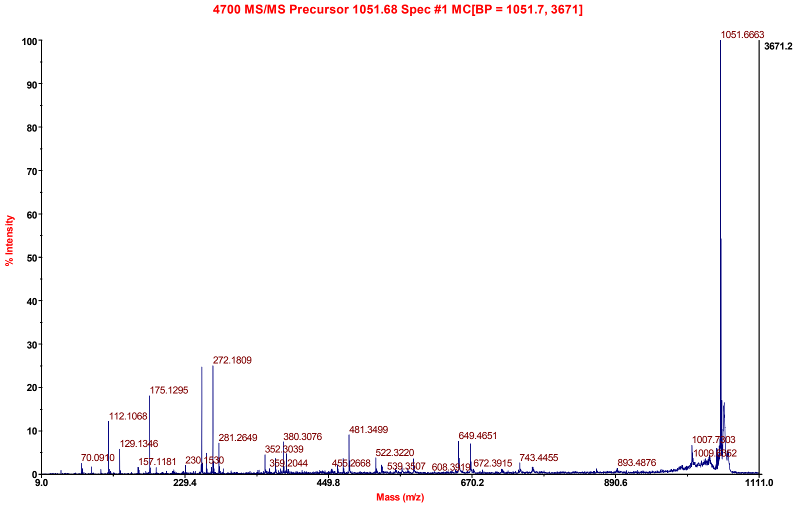

E:\...\C12_MSMS_1051.6774_4.t2d
Acquired:

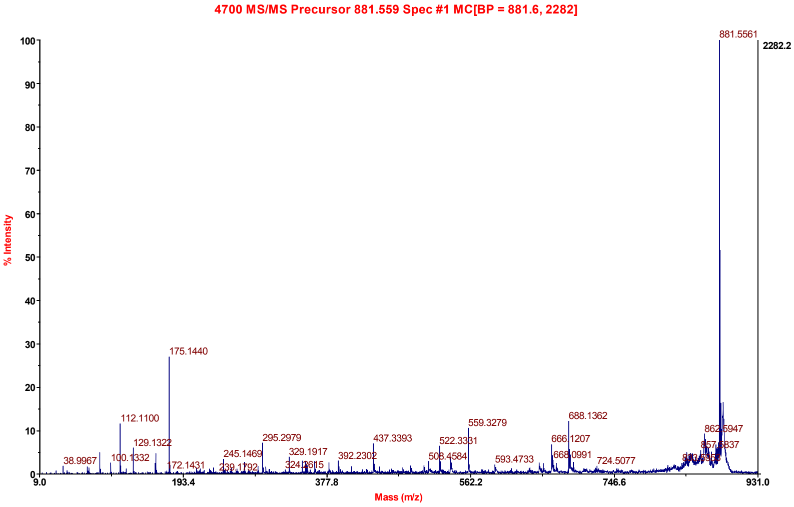

E:\...\C12_MSMS_881.5588_8.t2d
Acquired:

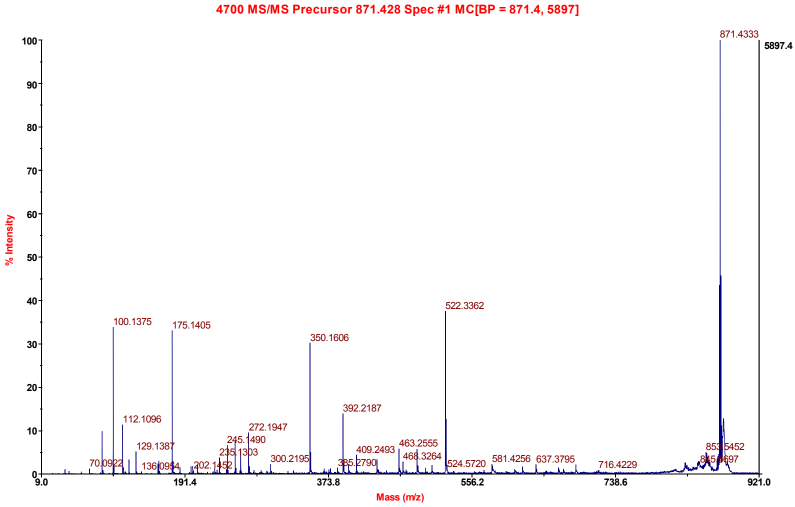

E:\...\C12_MSMS_871.4284_6.t2d
Acquired:
