# Supplementary material for: Physiological, Ultrastructural and Proteomic Responses in the Leaf of Maize Seedlings to Polyethylene Glycol-Stimulated Severe Water Deficiency
Source: Int J Mol Sci. 2015 Sep 8;16(9):21606–25. doi: 10.3390/ijms160921606 (PMC4613270; doi:10.3390/ijms160921606)
Supplement: Supplementary file 1 [file ijms-16-21606-s001.zip › ijms-96220-Supplementary Information/Supplementary File S2/MSMS-PDF/spot 11-C5.pdf]

4700 MS/MS Precursor 1747.95 Spec #1 MC[BP = 175.1, 436]

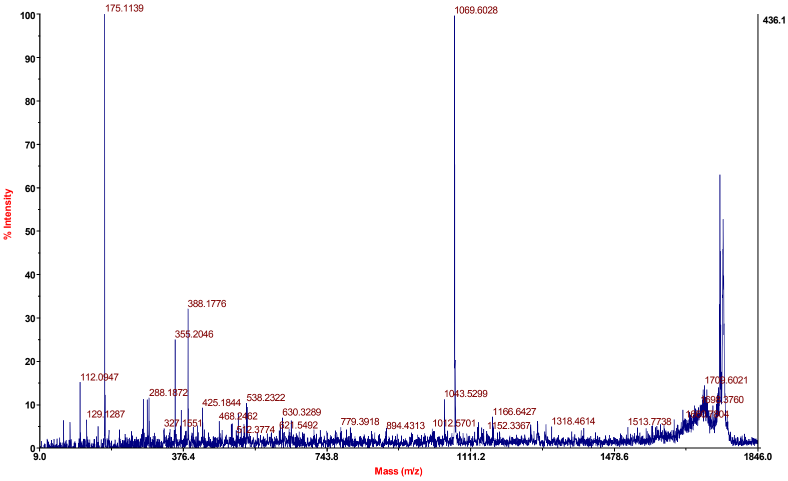

E:\...\C5_MSMS_1747.9469_4.t2d
Acquired:

4700 MS/MS Precursor 1651.69 Spec #1 MC[BP = 431.2, 231]

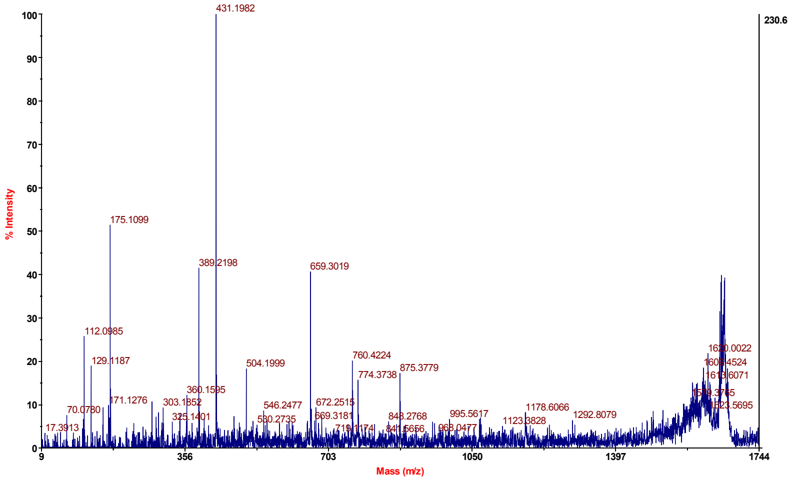

E:\...\C5_MSMS_1651.6931_8.t2d
Acquired:

4700 MS/MS Precursor 1575.74 Spec #1 MC[BP = 175.1, 218]

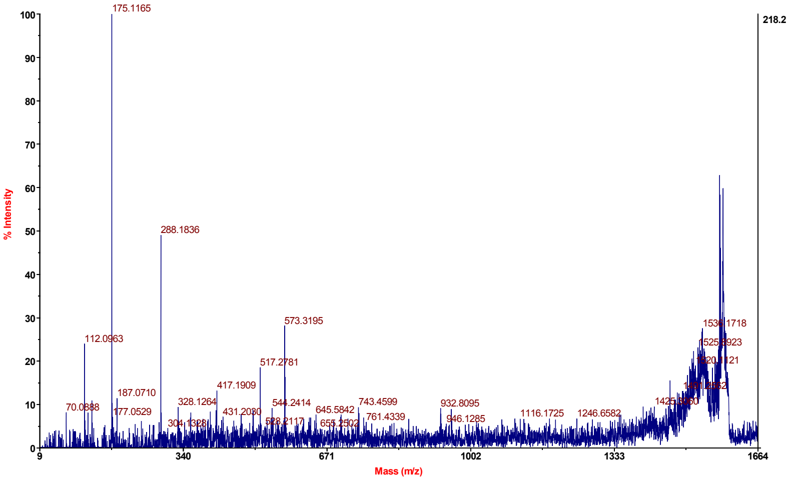

E:\...\C5_MSMS_1575.7383_7.t2d
Acquired:

4700 MS/MS Precursor 1541.76 Spec #1 MC[BP = 175.1, 171]

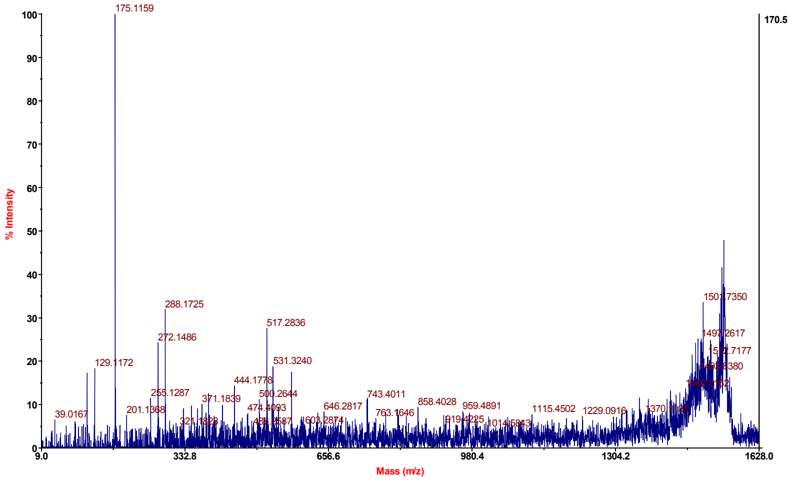

E:\...\C5_MSMS_1541.7626_11.t2d
Acquired:

4700 MS/MS Precursor 1344.62 Spec #1 MC[BP = 1344.7, 496]

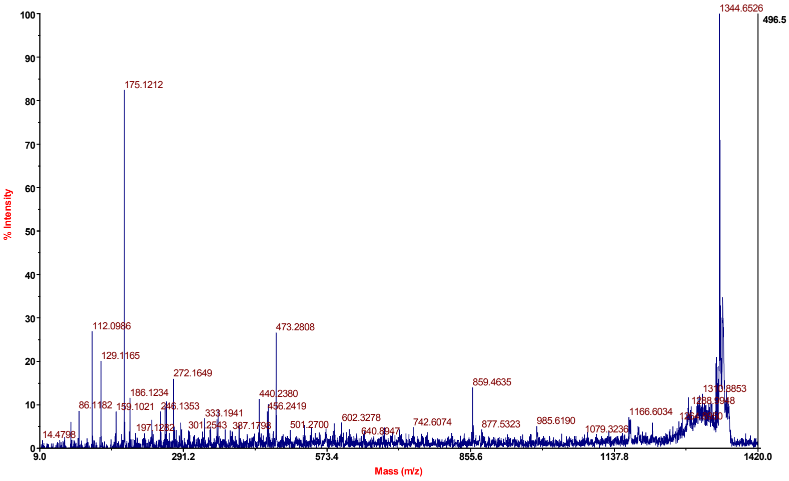

E:\...\C5_MSMS_1344.6162_6.t2d
Acquired:

4700 MS/MS Precursor 1337.64 Spec #1 MC[BP = 175.1, 250]

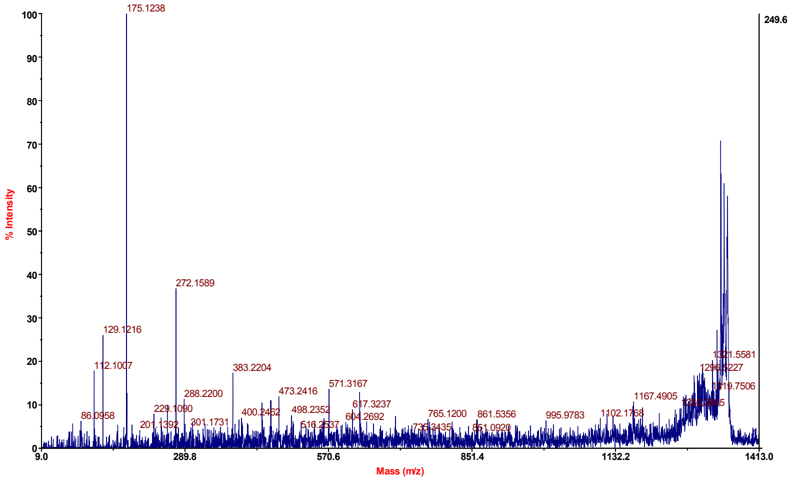

E:\...\C5_MSMS_1337.6367_9.t2d
Acquired:

4700 MS/MS Precursor 1051.64 Spec #1 MC[BP = 1051.6, 1400]

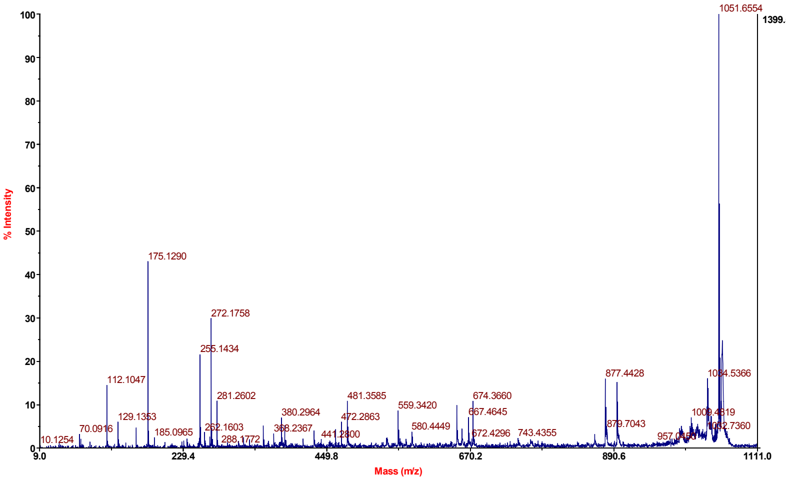

E:\...\C5_MSMS_1051.6407_2.t2d
Acquired:

4700 MS/MS Precursor 970.42 Spec #1 MC[BP = 175.1, 116]

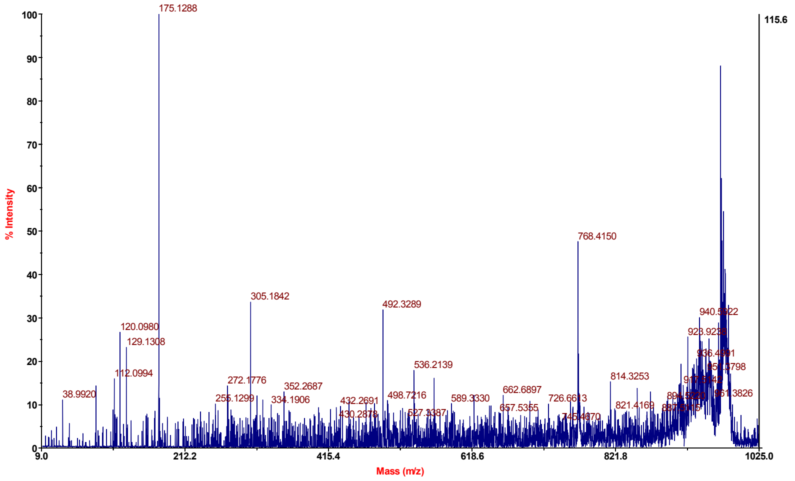

E:\...\C5_MSMS_970.4200_10.t2d
Acquired:
