# Supplementary material for: Physiological, Ultrastructural and Proteomic Responses in the Leaf of Maize Seedlings to Polyethylene Glycol-Stimulated Severe Water Deficiency
Source: Int J Mol Sci. 2015 Sep 8;16(9):21606–25. doi: 10.3390/ijms160921606 (PMC4613270; doi:10.3390/ijms160921606)
Supplement: Supplementary file 1 [file ijms-16-21606-s001.zip › ijms-96220-Supplementary Information/Supplementary File S2/MSMS-PDF/spot 13-C11.pdf]

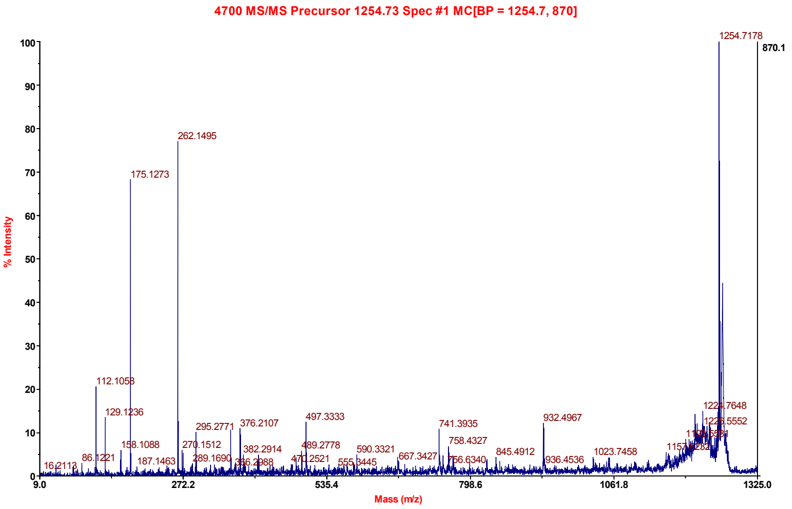

E:\...\C11_MSMS_1254.7328_5.t2d
Acquired:

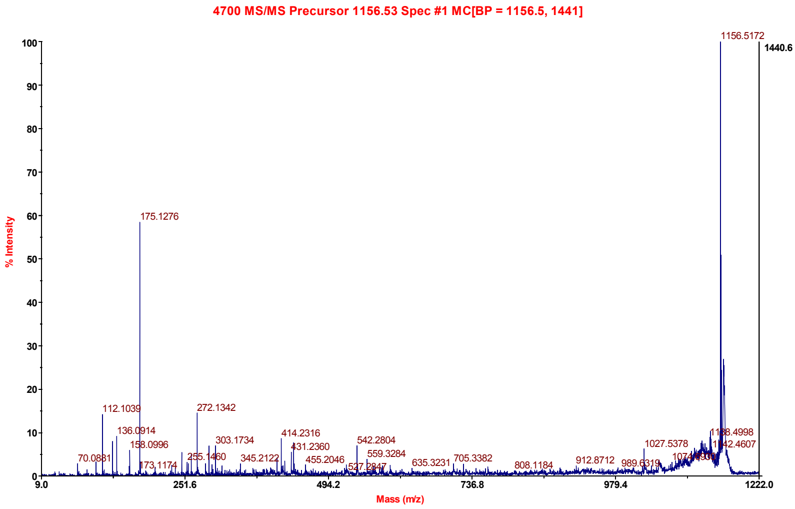

E:\...\C11_MSMS_1156.5275_4.t2d
Acquired:

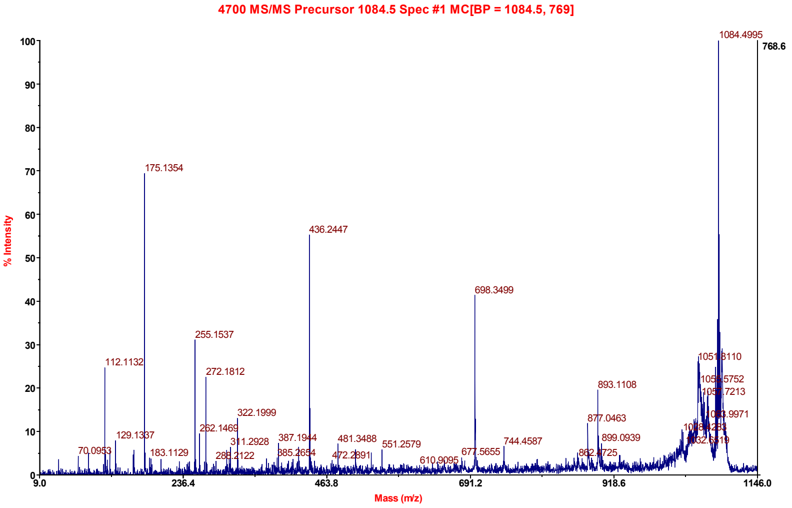

E:\...\C11_MSMS_1084.5043_6.t2d
Acquired:

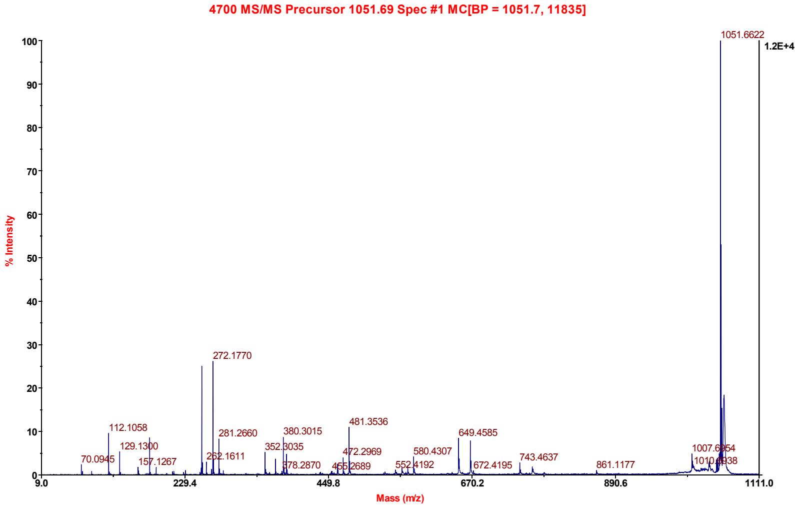

E:\...\C11_MSMS_1051.6863_2.t2d
Acquired:

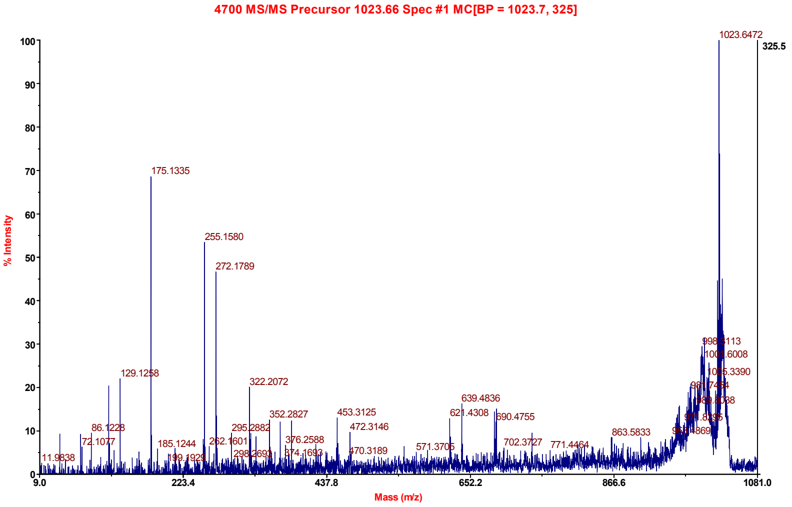

E:\...\C11_MSMS_1023.6606_10.t2d
Acquired:

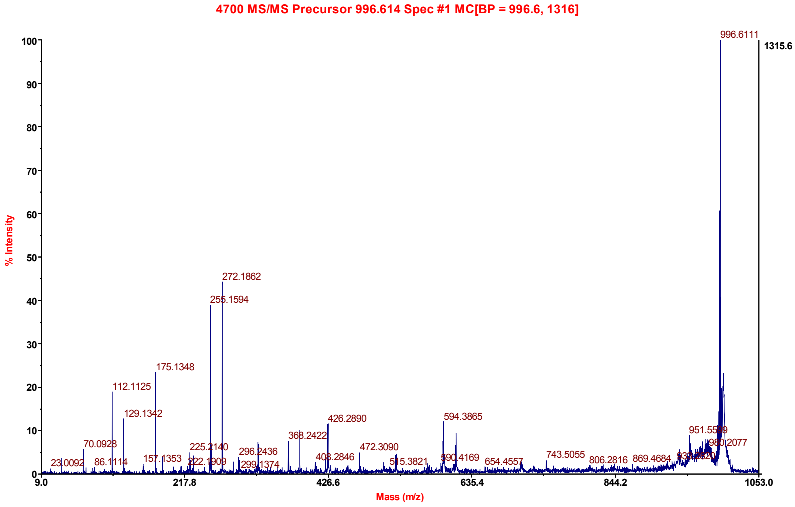

E:\...\C11_MSMS_996.6144_7.t2d
Acquired:

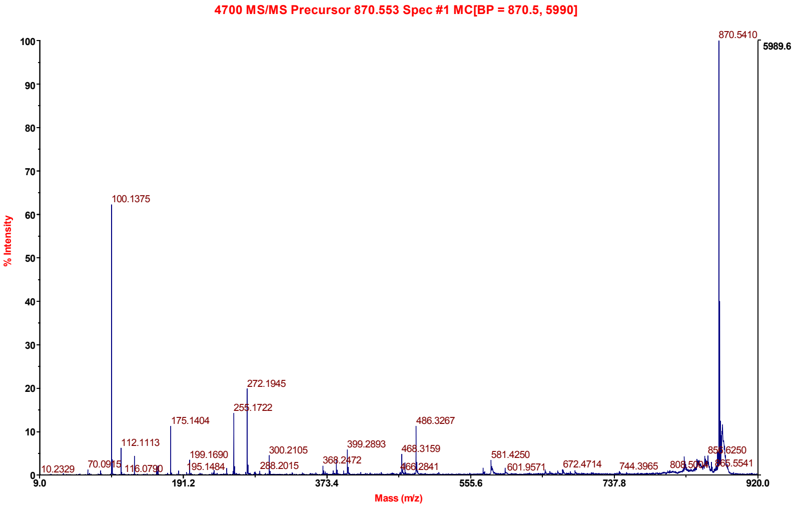

E:\...\C11_MSMS_870.5528_3.t2d
Acquired:

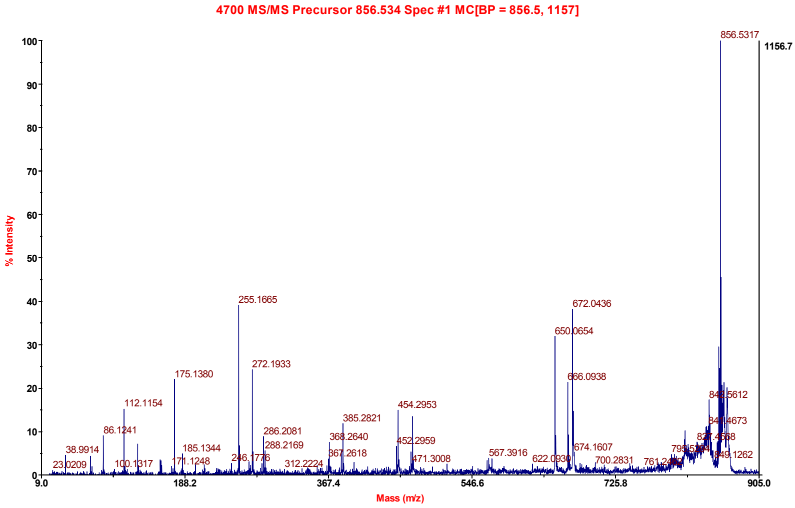

E:\...\C11_MSMS_856.5344_8.t2d
Acquired:
