# Supplementary material for: Physiological, Ultrastructural and Proteomic Responses in the Leaf of Maize Seedlings to Polyethylene Glycol-Stimulated Severe Water Deficiency
Source: Int J Mol Sci. 2015 Sep 8;16(9):21606–25. doi: 10.3390/ijms160921606 (PMC4613270; doi:10.3390/ijms160921606)
Supplement: Supplementary file 1 [file ijms-16-21606-s001.zip › ijms-96220-Supplementary Information/Supplementary File S2/MSMS-PDF/spot 14-C9.pdf]

4700 MS/MS Precursor 2080.84 Spec #1 MC[BP = 417.2, 1043]

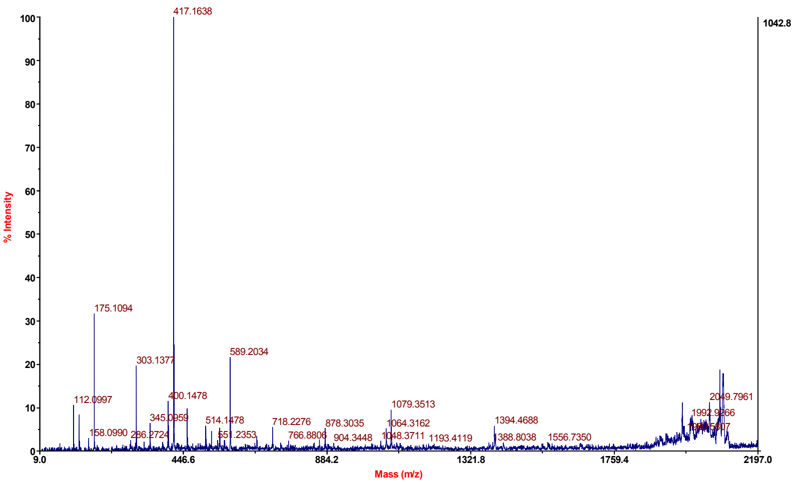

E:\...\C9_MSMS_2080.8398_9.t2d
Acquired:

4700 MS/MS Precursor 2048.86 Spec #1 MC[BP = 417.2, 4573]

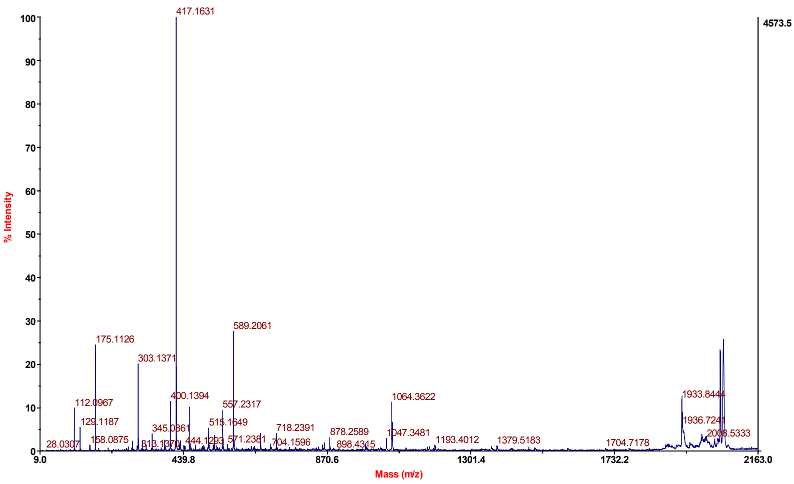

E:\...\C9_MSMS_2048.8564_2.t2d
Acquired:

4700 MS/MS Precursor 1896.83 Spec #1 MC[BP = 1106.5, 1336]

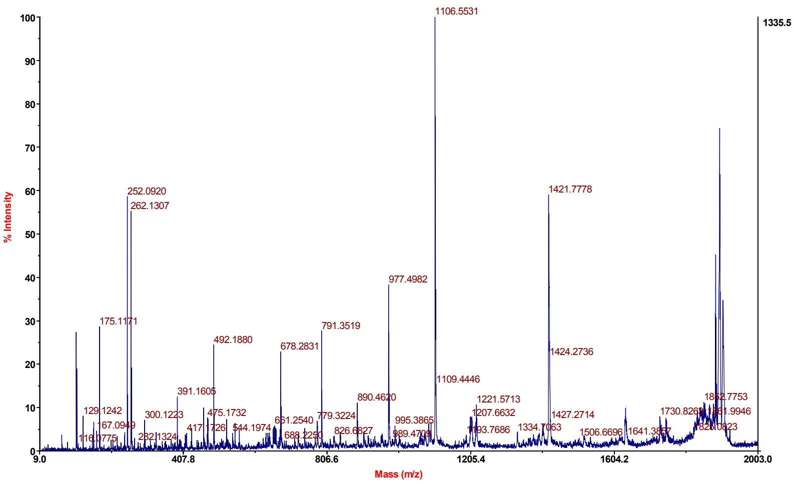

E:\...\C9_MSMS_1896.8290_6.t2d
Acquired:

4700 MS/MS Precursor 1884.82 Spec #1 MC[BP = 1884.8, 1549]

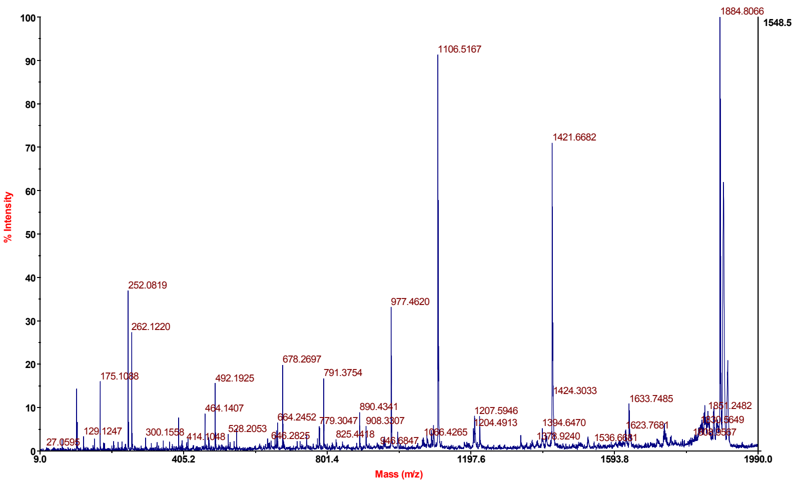

E:\...\C9_MSMS_1884.8170_3.t2d
Acquired:

4700 MS/MS Precursor 1673.83 Spec #1 MC[BP = 853.5, 784]

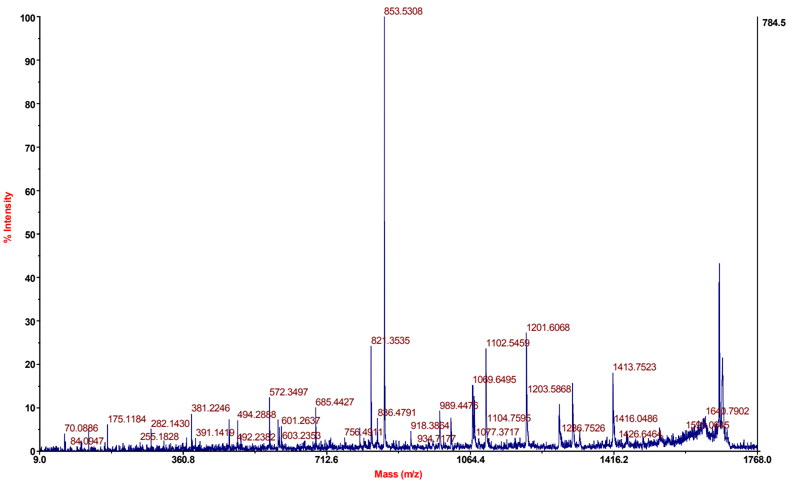

E:\...\C9_MSMS_1673.8259_8.t2d
Acquired:

4700 MS/MS Precursor 1499.63 Spec #1 MC[BP = 1499.7, 1221]

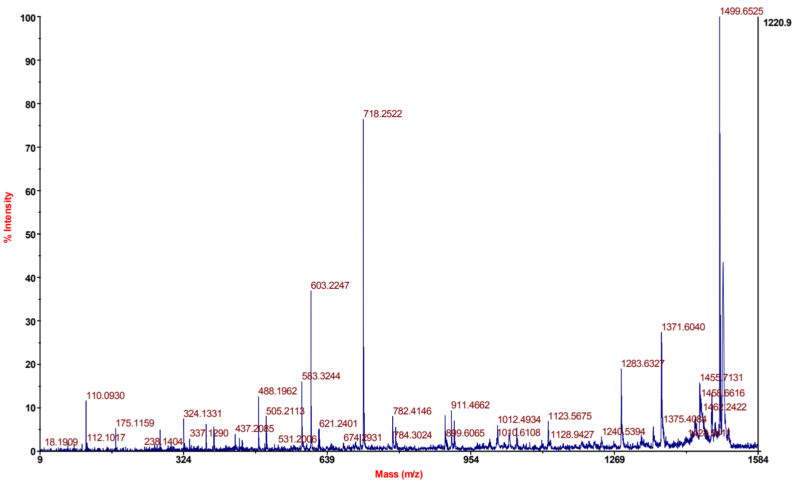

E:\...\C9_MSMS_1499.6293_5.t2d
Acquired:

4700 MS/MS Precursor 1444.73 Spec #1 MC[BP = 1444.8, 836]

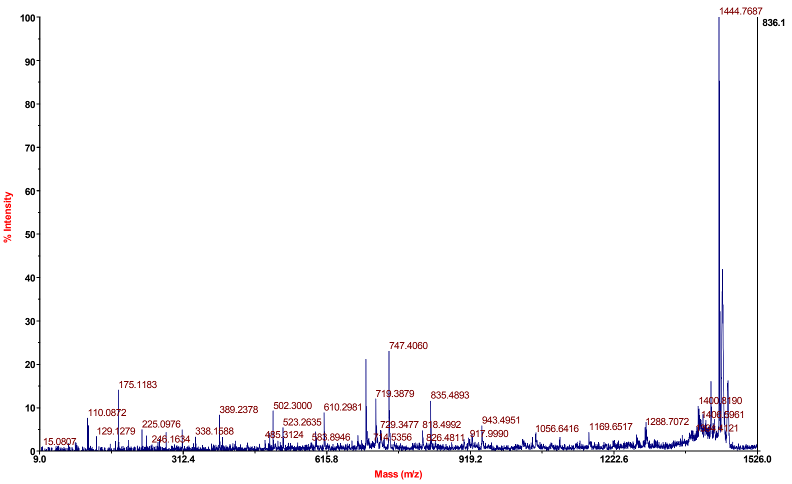

E:\...\C9_MSMS_1444.7329_7.t2d
Acquired:

4700 MS/MS Precursor 1196.55 Spec #1 MC[BP = 1196.5, 808]

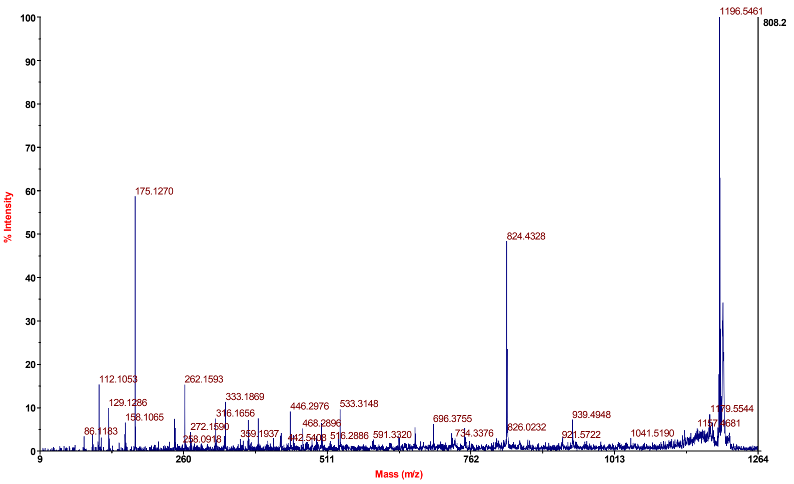

E:\...\C9_MSMS_1196.5525_11.t2d
Acquired:
