# Supplementary material for: Physiological, Ultrastructural and Proteomic Responses in the Leaf of Maize Seedlings to Polyethylene Glycol-Stimulated Severe Water Deficiency
Source: Int J Mol Sci. 2015 Sep 8;16(9):21606–25. doi: 10.3390/ijms160921606 (PMC4613270; doi:10.3390/ijms160921606)
Supplement: Supplementary file 1 [file ijms-16-21606-s001.zip › ijms-96220-Supplementary Information/Supplementary File S2/MSMS-PDF/spot 15-C7.pdf]

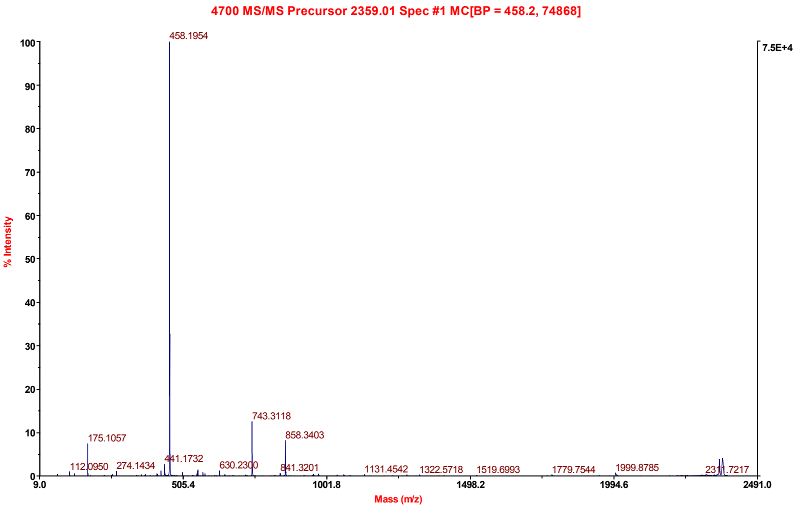

E:\...\C7_MSMS_2359.0127_2.t2d
Acquired:

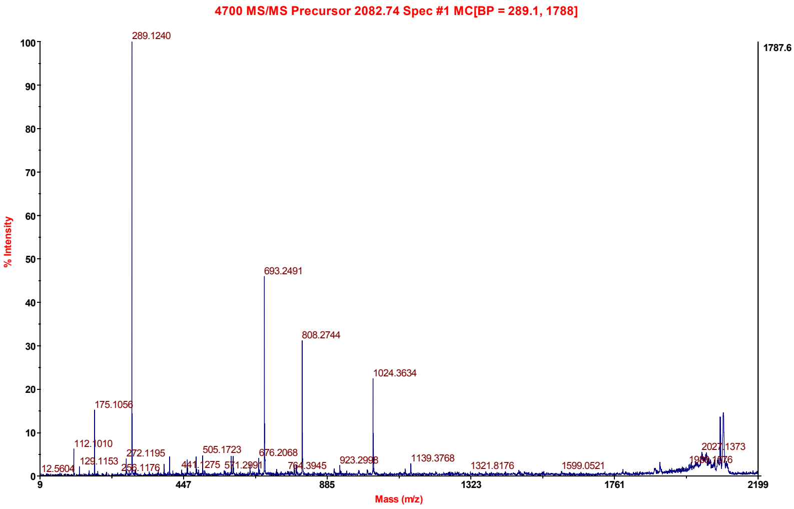

E:\...\C7_MSMS_2082.7446_11.t2d
Acquired:

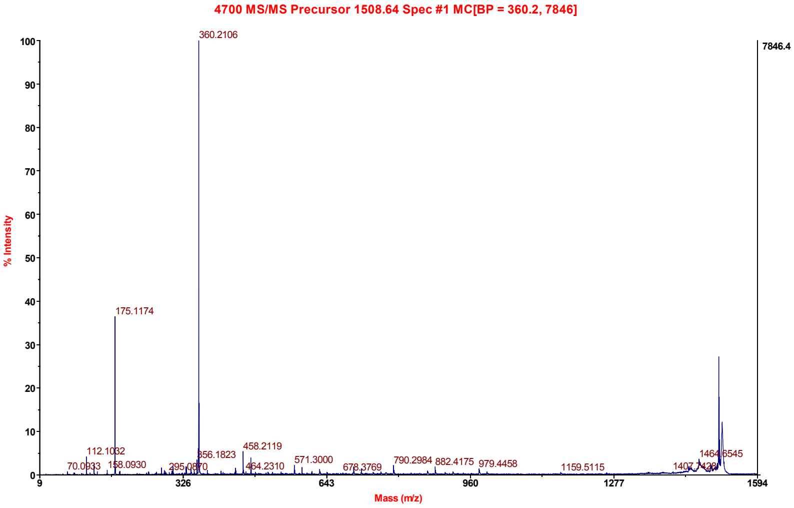

E:\...\C7_MSMS_1508.6381_5.t2d
Acquired:

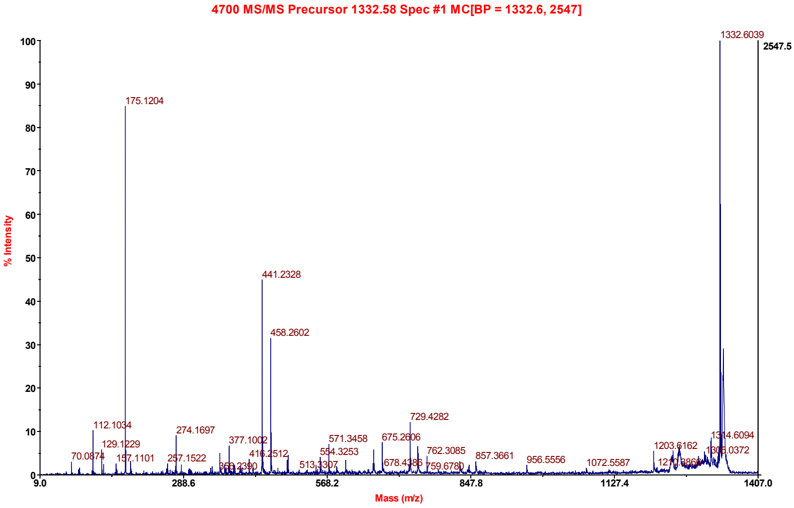

E:\...\C7_MSMS_1332.5847_6.t2d
Acquired:

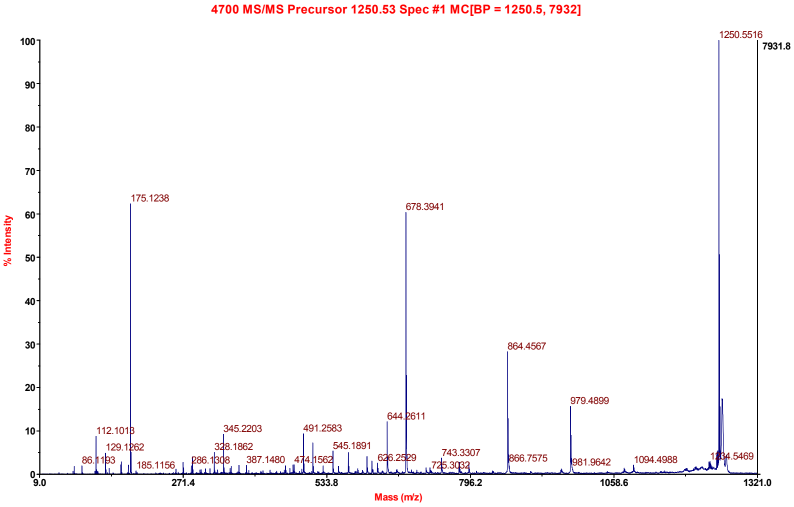

E:\...\C7_MSMS_1250.5287_3.t2d
Acquired:

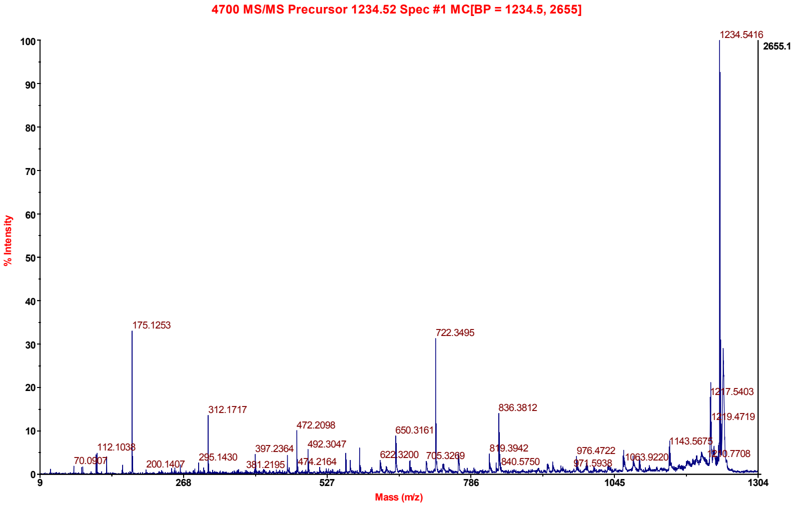

E:\...\C7_MSMS_1234.5220_7.t2d
Acquired:

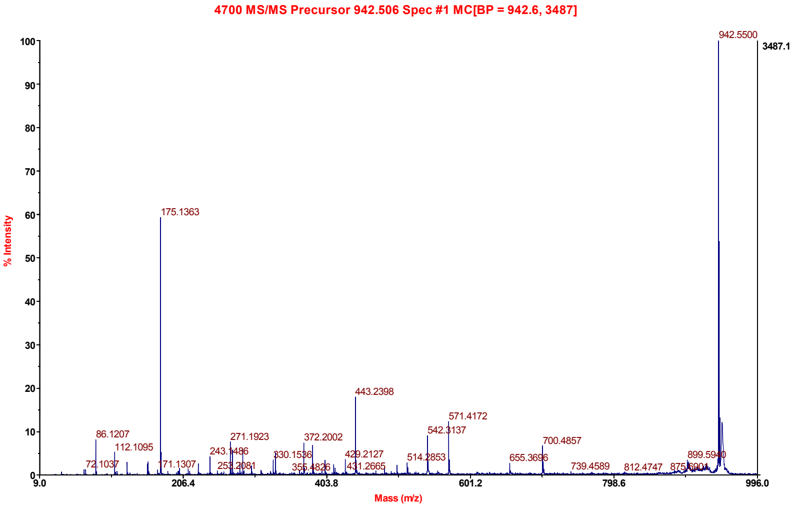

E:\...\C7_MSMS_942.5059_10.t2d
Acquired:

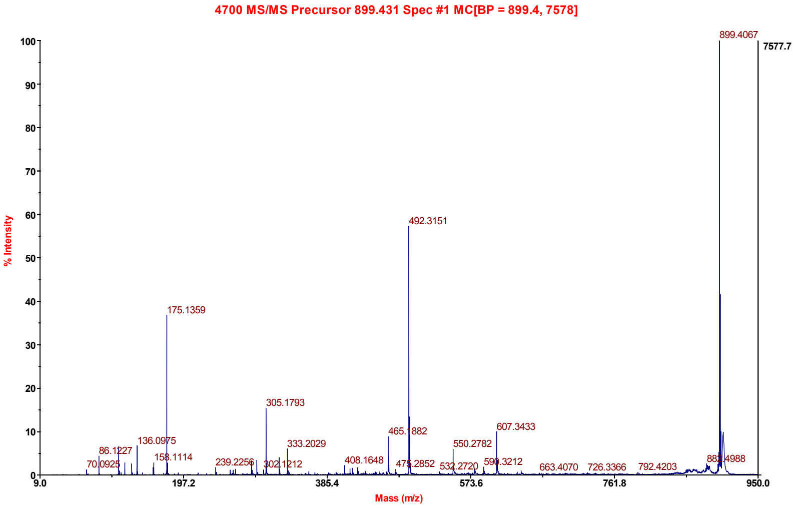

E:\...\C7_MSMS_899.4311_4.t2d
Acquired:
