# Supplementary material for: Physiological, Ultrastructural and Proteomic Responses in the Leaf of Maize Seedlings to Polyethylene Glycol-Stimulated Severe Water Deficiency
Source: Int J Mol Sci. 2015 Sep 8;16(9):21606–25. doi: 10.3390/ijms160921606 (PMC4613270; doi:10.3390/ijms160921606)
Supplement: Supplementary file 1 [file ijms-16-21606-s001.zip › ijms-96220-Supplementary Information/Supplementary File S2/MSMS-PDF/spot 16-C3.pdf]

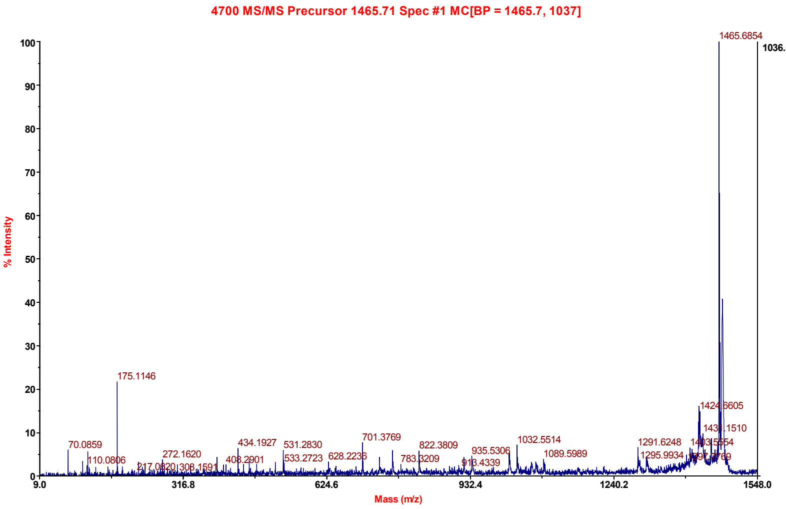

E:\...\C3_MSMS_1465.7111_2.t2d
Acquired:

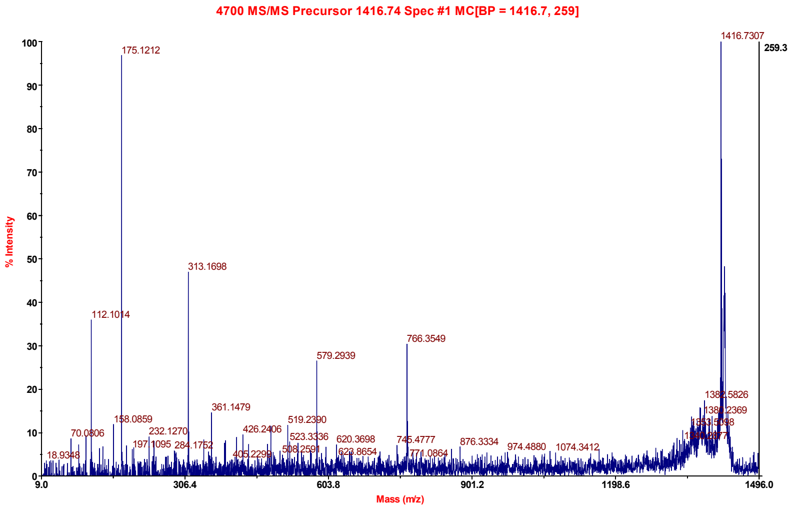

E:\...\C3_MSMS_1416.7389_9.t2d
Acquired:

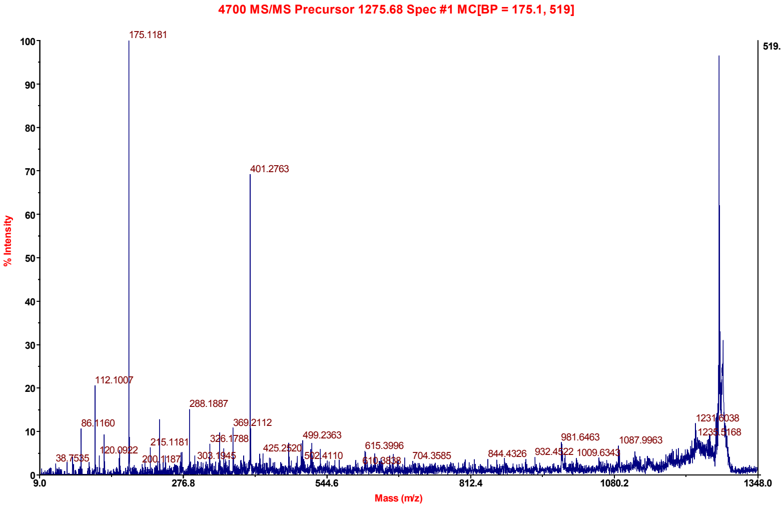

E:\...\C3_MSMS_1275.6847_5.t2d
Acquired:

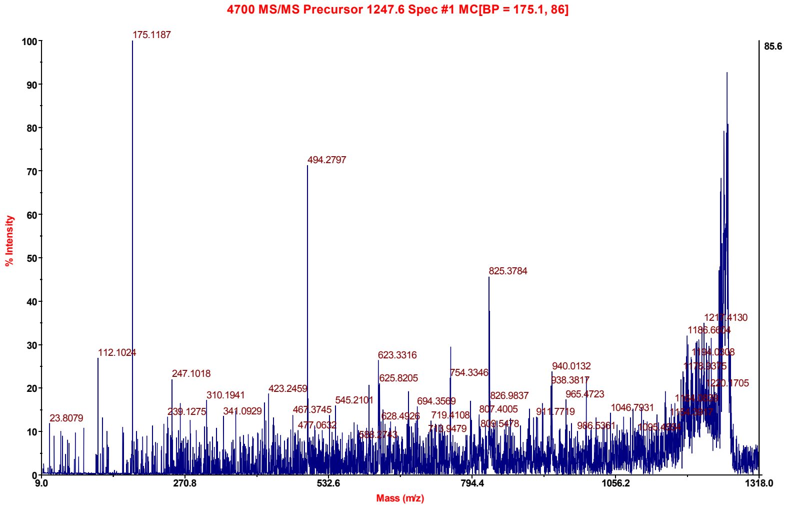

E:\...\C3_MSMS_1247.6023_10.t2d
Acquired:

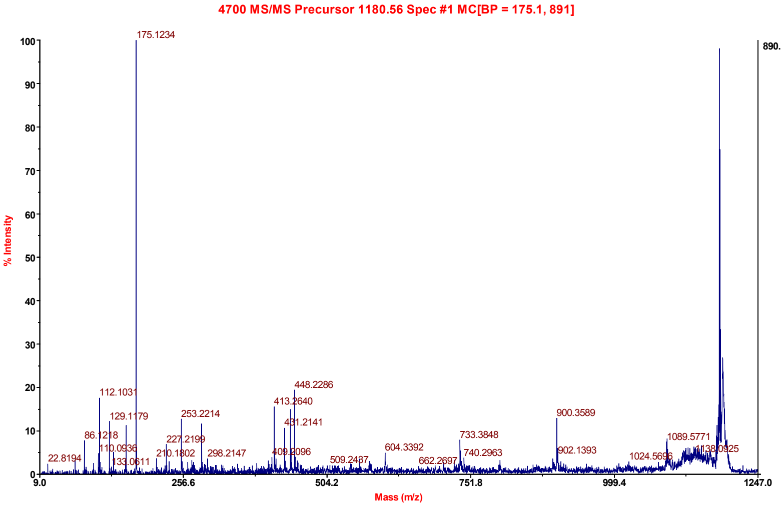

E:\...\C3_MSMS_1180.5647_3.t2d
Acquired:

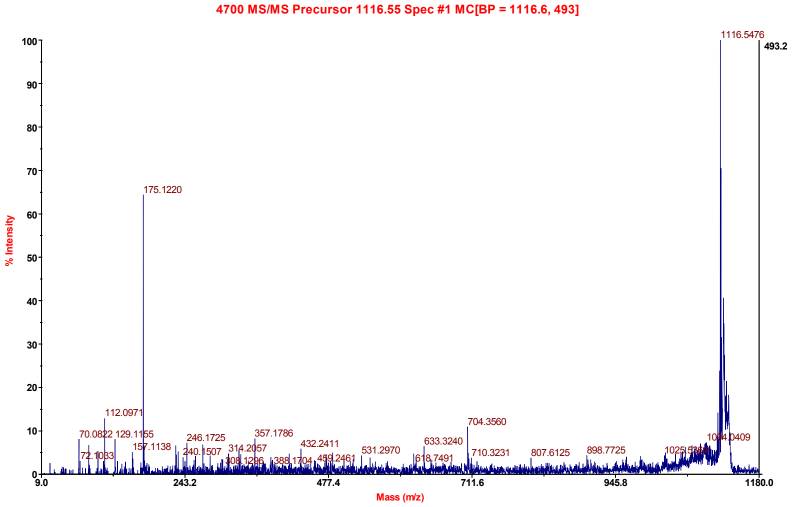

E:\...\C3_MSMS_1116.5504_6.t2d
Acquired:

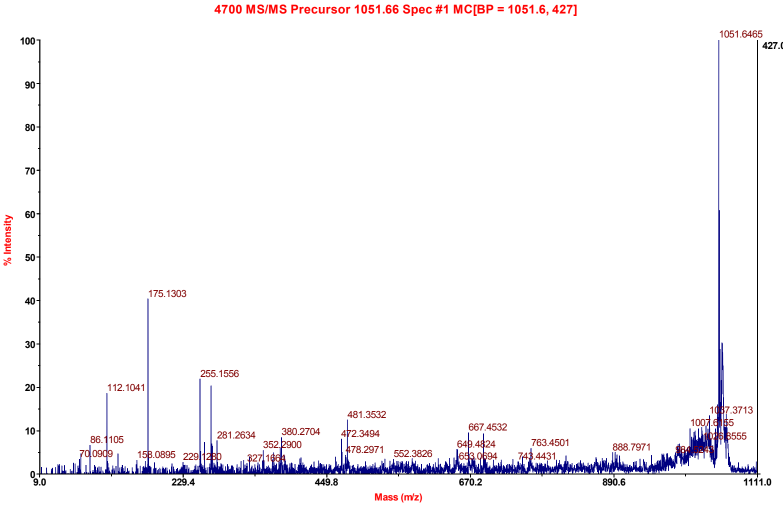

E:\...\C3_MSMS_1051.6584_7.t2d
Acquired:

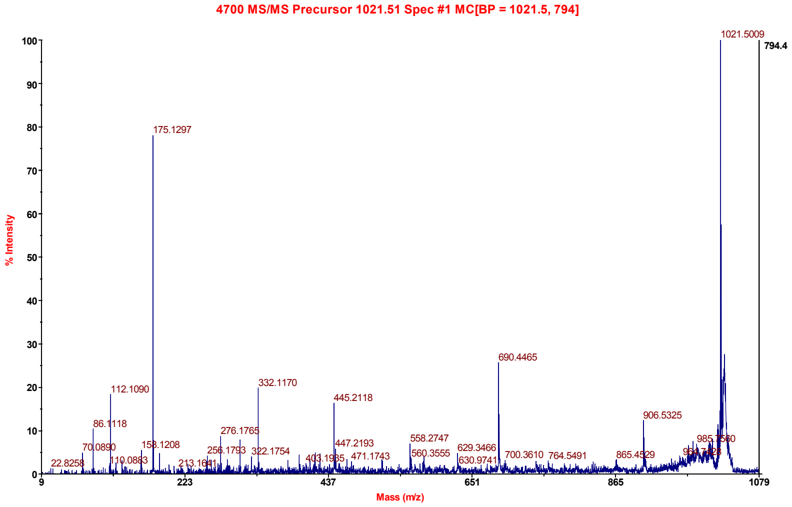

E:\...\C3_MSMS_1021.5083_4.t2d
Acquired:
