# Supplementary material for: Physiological, Ultrastructural and Proteomic Responses in the Leaf of Maize Seedlings to Polyethylene Glycol-Stimulated Severe Water Deficiency
Source: Int J Mol Sci. 2015 Sep 8;16(9):21606–25. doi: 10.3390/ijms160921606 (PMC4613270; doi:10.3390/ijms160921606)
Supplement: Supplementary file 1 [file ijms-16-21606-s001.zip › ijms-96220-Supplementary Information/Supplementary File S2/MSMS-PDF/spot 2-D3.pdf]

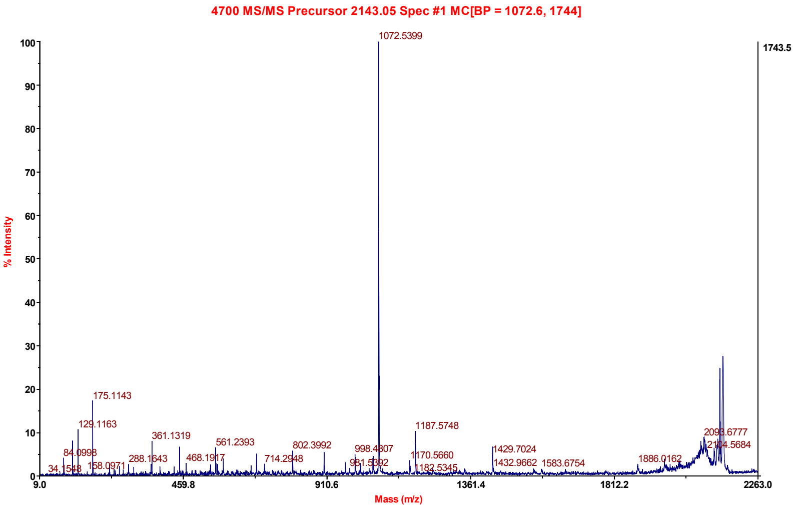

E:\...\D3_MSMS_2143.0537_3.t2d
Acquired:

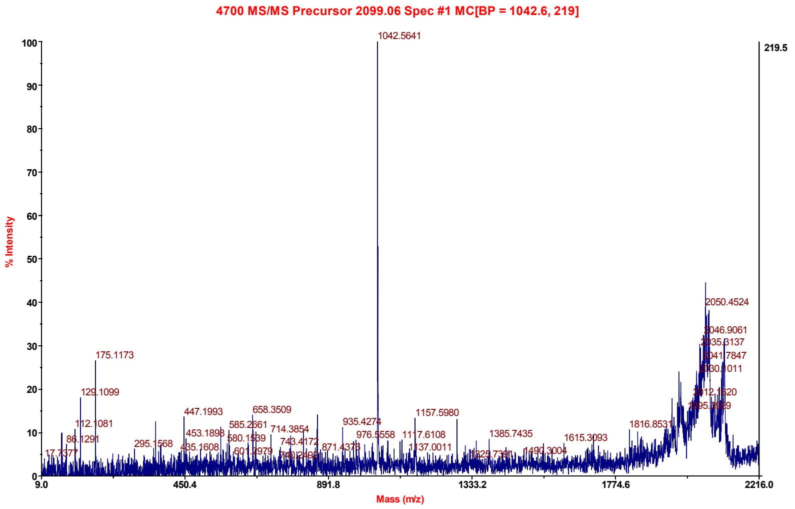

E:\...\D3_MSMS_2099.0608_10.t2d
Acquired:

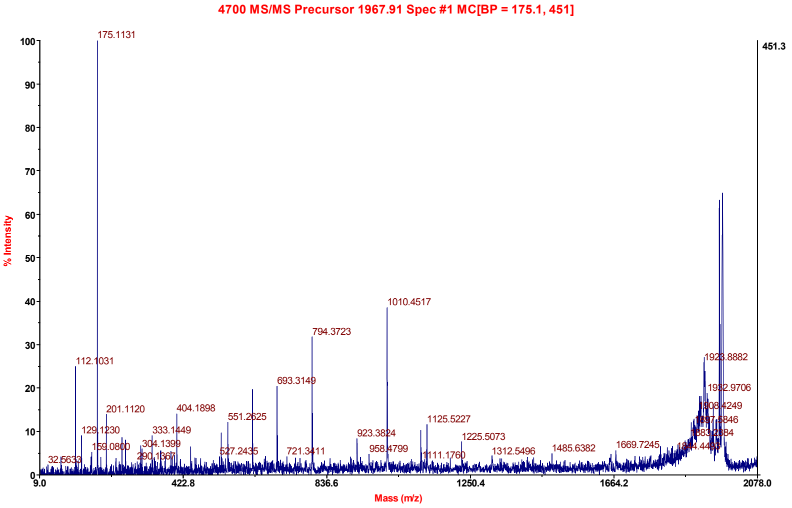

E:\...\D3_MSMS_1967.9098_7.t2d
Acquired:

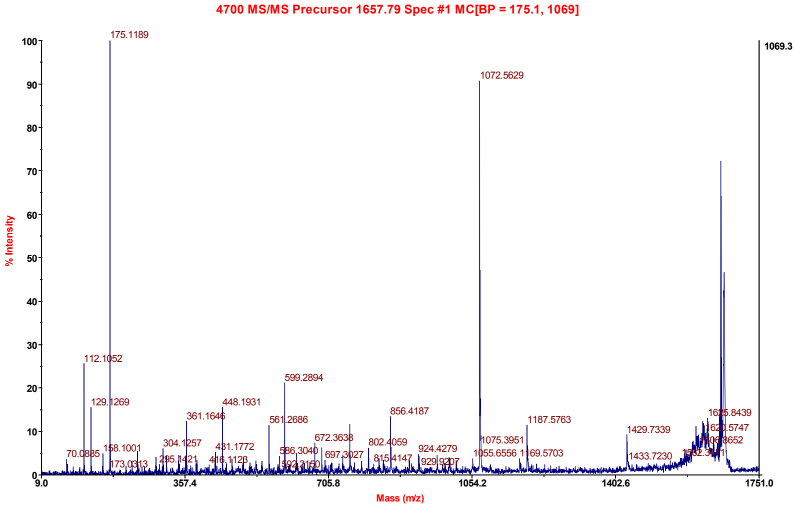

E:\...\D3_MSMS_1657.7876_5.t2d
Acquired:

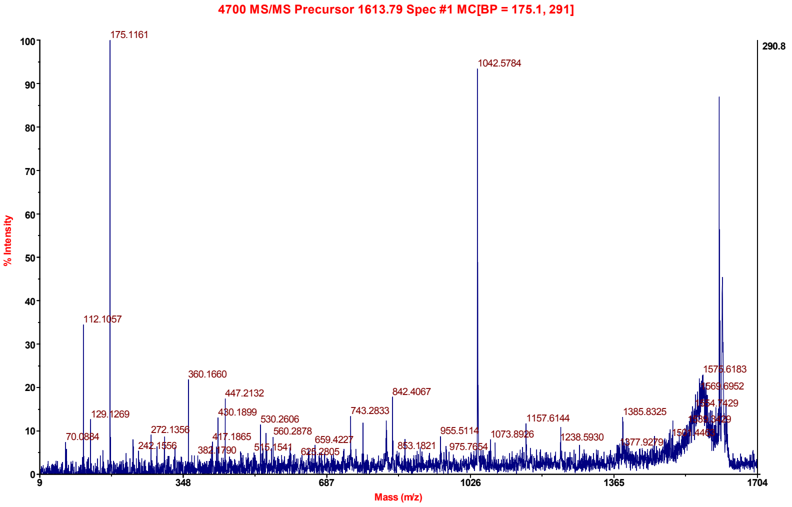

E:\...\D3_MSMS_1613.7863_9.t2d
Acquired:

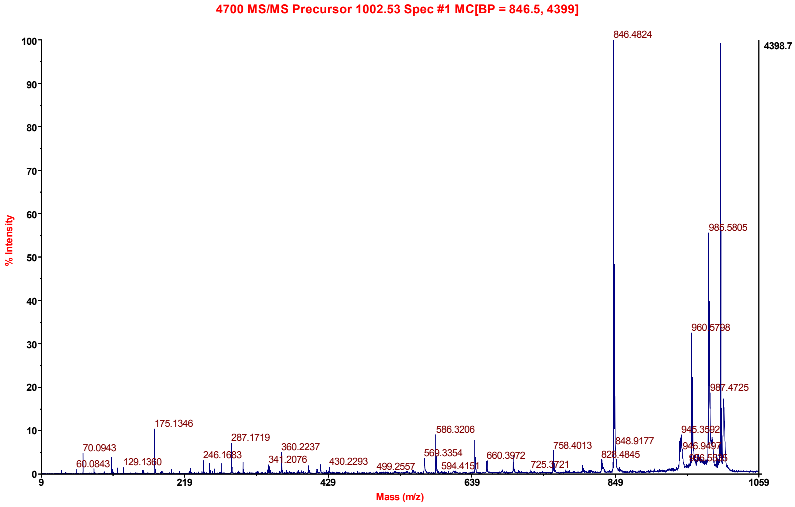

E:\...\D3_MSMS_1002.5262_4.t2d
Acquired:

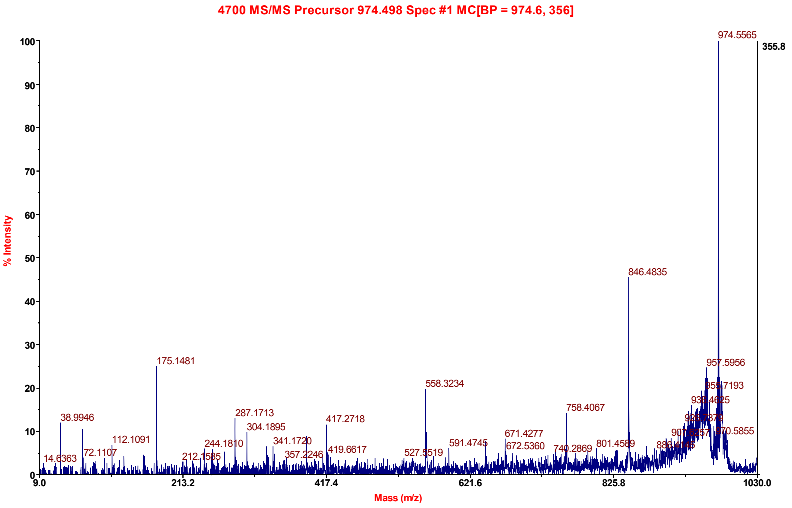

E:\...\D3_MSMS_974.4985_11.t2d
Acquired:

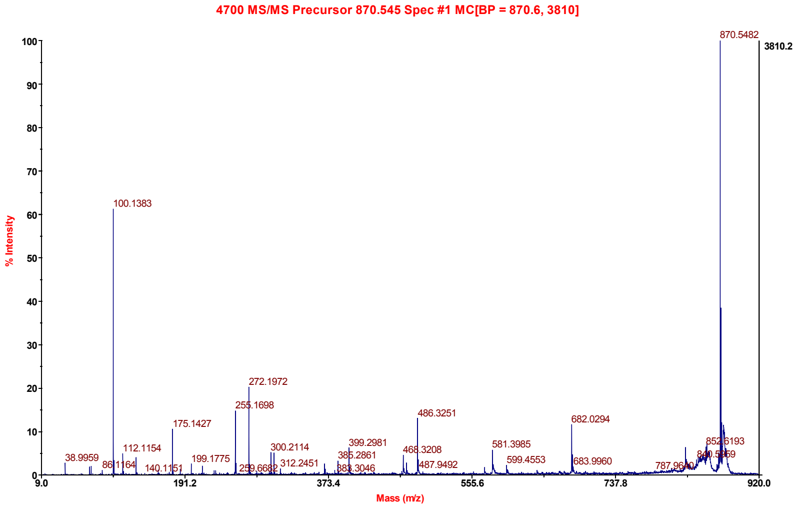

E:\...\D3_MSMS_870.5446_6.t2d
Acquired:
