# Supplementary material for: Physiological, Ultrastructural and Proteomic Responses in the Leaf of Maize Seedlings to Polyethylene Glycol-Stimulated Severe Water Deficiency
Source: Int J Mol Sci. 2015 Sep 8;16(9):21606–25. doi: 10.3390/ijms160921606 (PMC4613270; doi:10.3390/ijms160921606)
Supplement: Supplementary file 1 [file ijms-16-21606-s001.zip › ijms-96220-Supplementary Information/Supplementary File S2/MSMS-PDF/spot 5-C10.pdf]

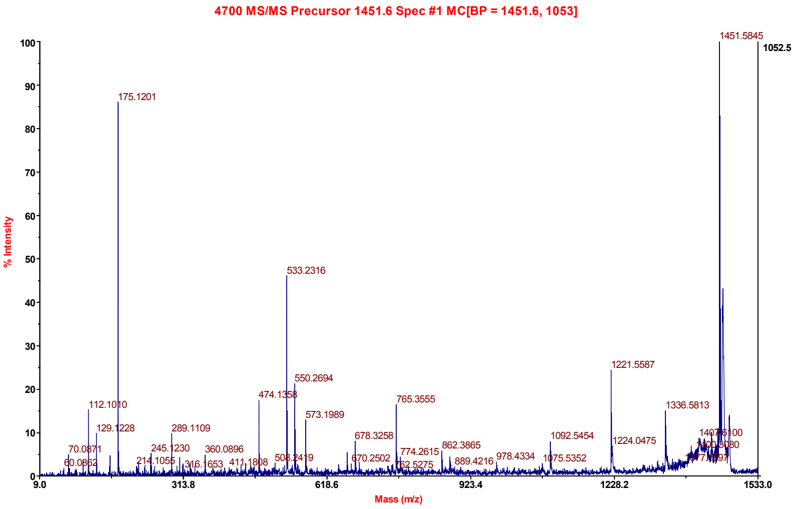

E:\...\C10_MSMS_1451.6038_6.t2d
Acquired:

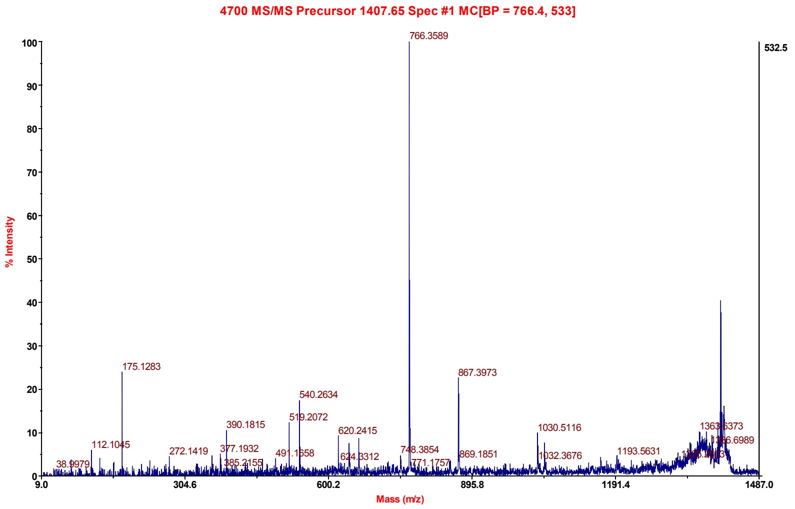

E:\...\C10_MSMS_1407.6472_10.t2d
Acquired:

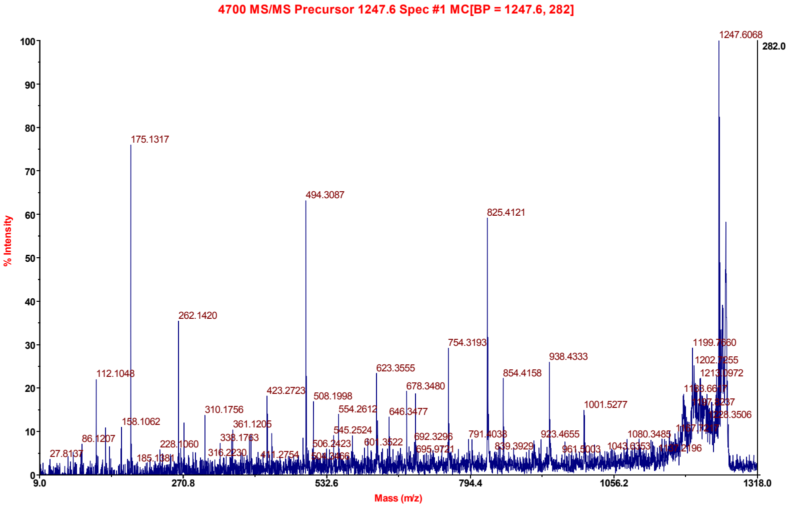

E:\...\C10_MSMS_1247.5986_9.t2d
Acquired:

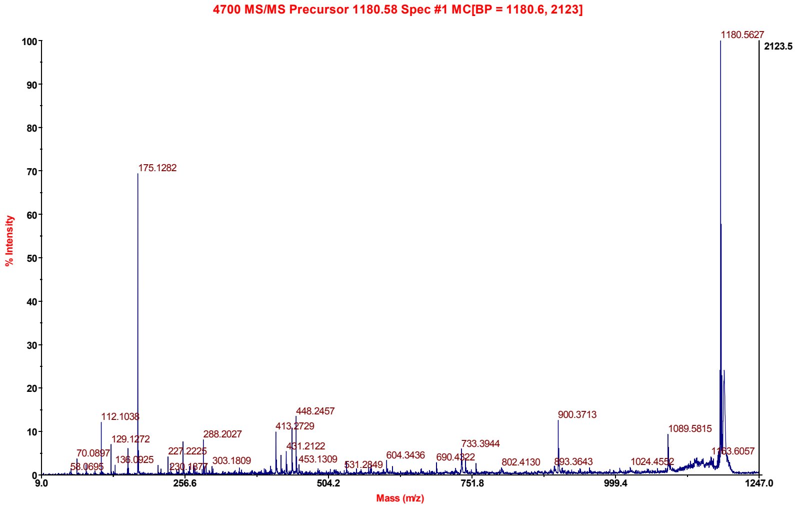

E:\...\C10_MSMS_1180.5775_5.t2d
Acquired:

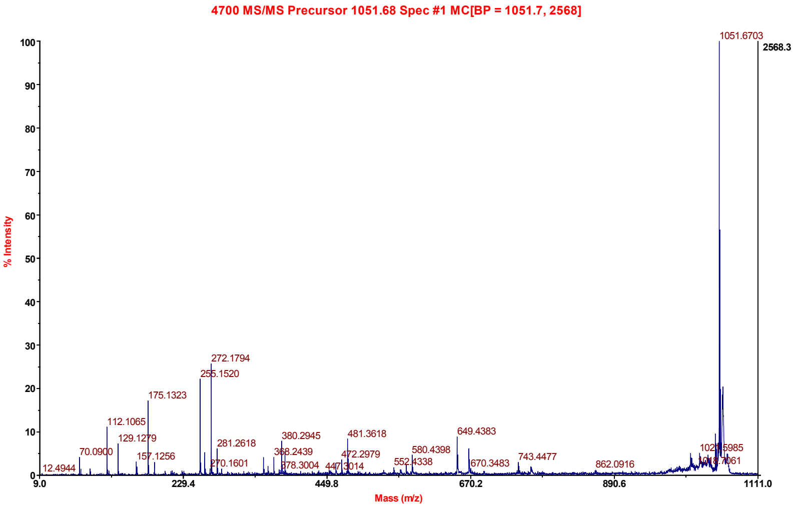

E:\...\C10_MSMS_1051.6805_4.t2d
Acquired:

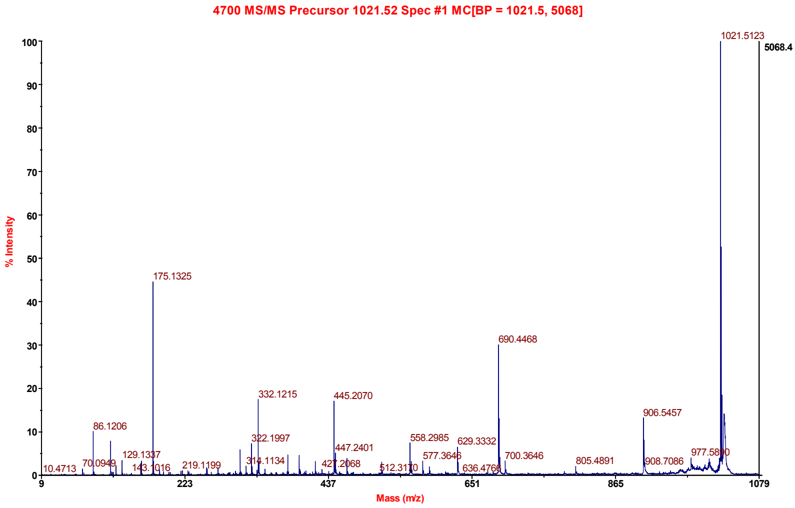

E:\...\C10_MSMS_1021.5225_2.t2d
Acquired:

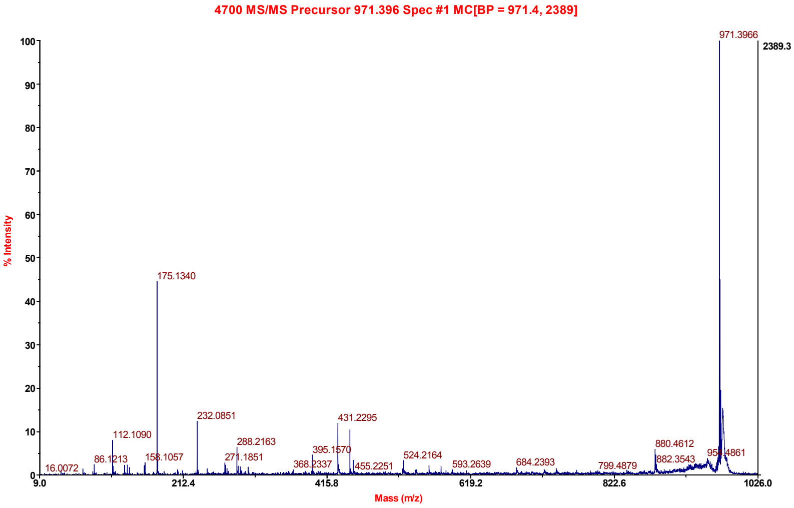

E:\...\C10_MSMS_971.3958_7.t2d
Acquired:

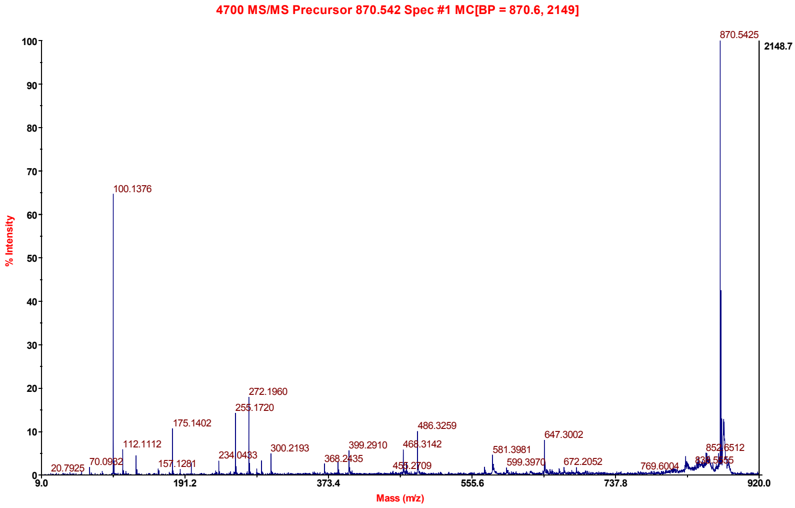

E:\...\C10_MSMS_870.5416_8.t2d
Acquired:
