# Supplementary material for: Physiological, Ultrastructural and Proteomic Responses in the Leaf of Maize Seedlings to Polyethylene Glycol-Stimulated Severe Water Deficiency
Source: Int J Mol Sci. 2015 Sep 8;16(9):21606–25. doi: 10.3390/ijms160921606 (PMC4613270; doi:10.3390/ijms160921606)
Supplement: Supplementary file 1 [file ijms-16-21606-s001.zip › ijms-96220-Supplementary Information/Supplementary File S2/MSMS-PDF/spot 6-D1.pdf]

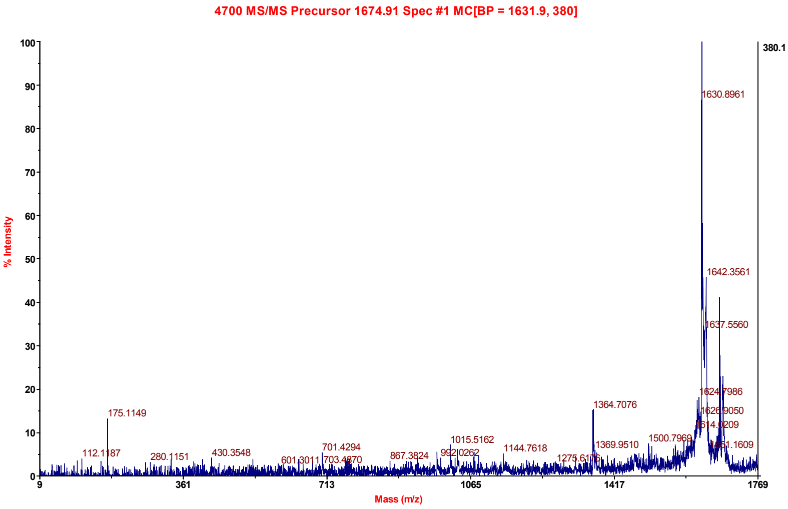

E:\...\D1_MSMS_1674.9070_10.t2d
Acquired:

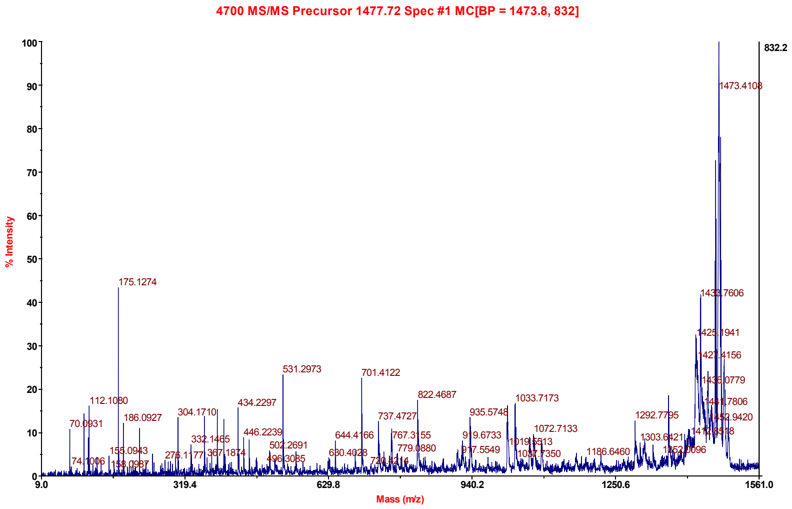

E:\...\D1_MSMS_1477.7185_9.t2d
Acquired:

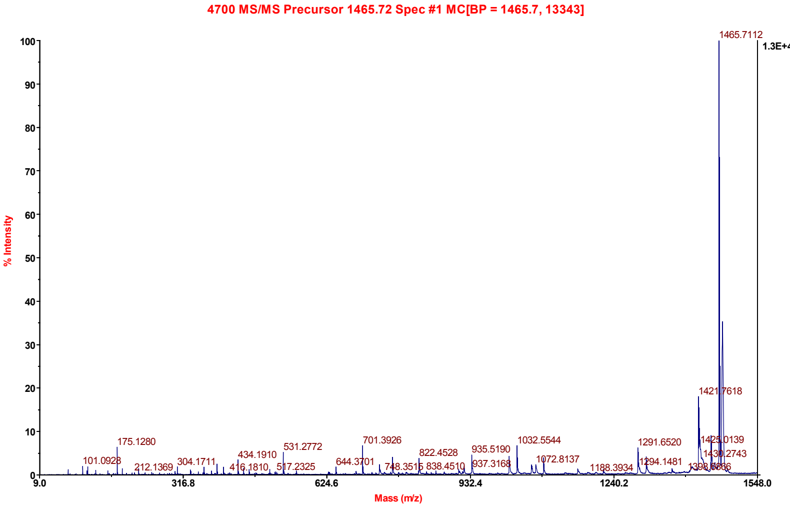

E:\...\D1_MSMS_1465.7208_2.t2d
Acquired:

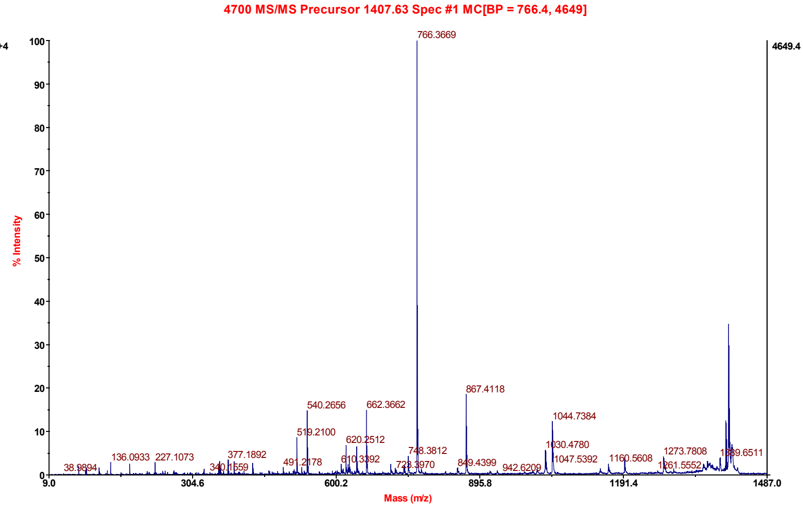

E:\...\D1_MSMS_1407.6305_4.t2d
Acquired:

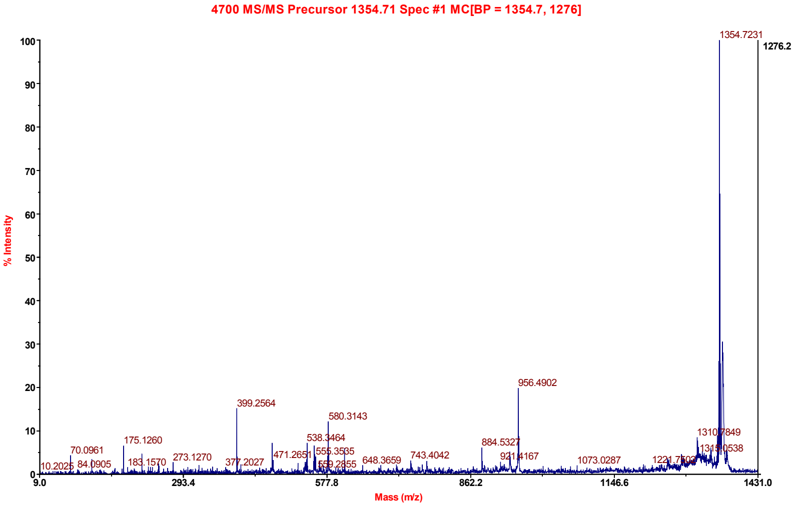

E:\...\D1_MSMS_1354.7091_7.t2d
Acquired:

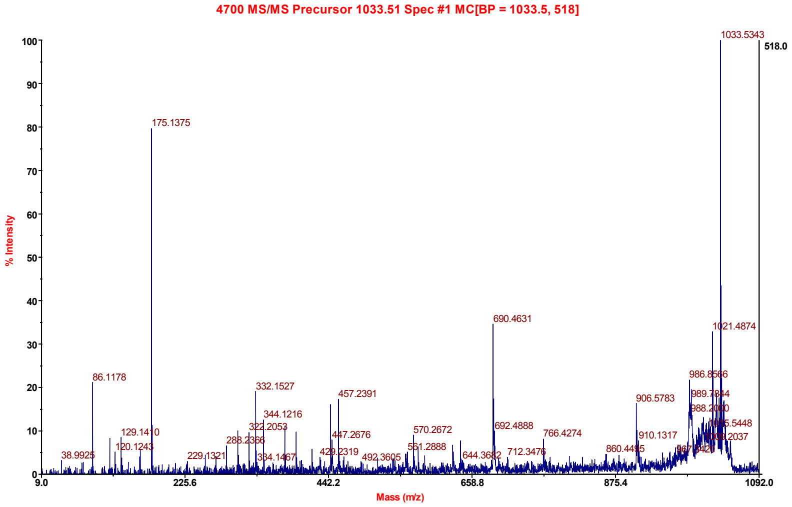

E:\...\D1_MSMS_1033.5090_11.t2d
Acquired:

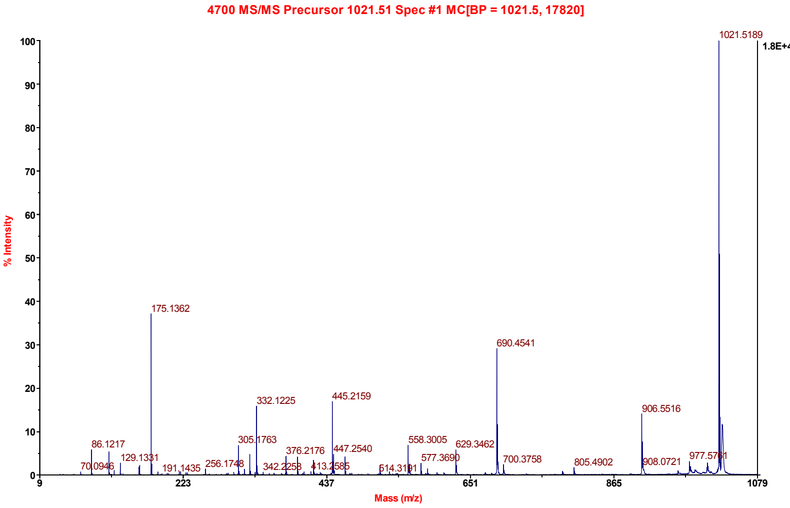

E:\...\D1_MSMS_1021.5063_3.t2d
Acquired:

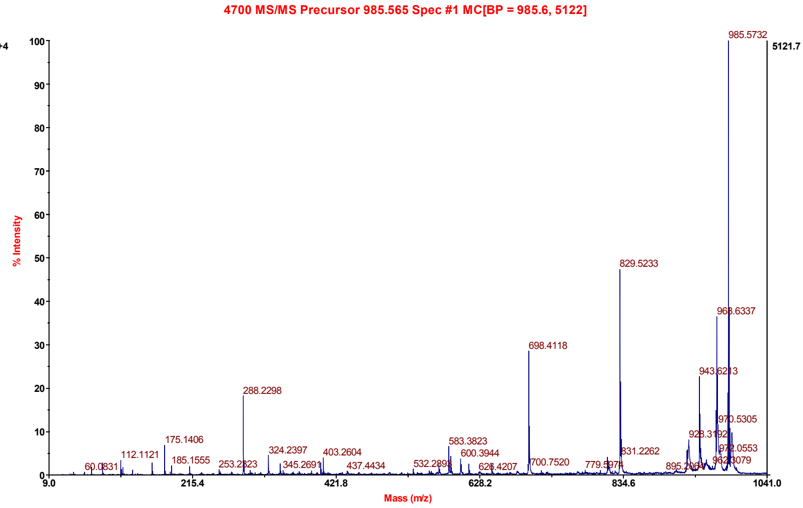

E:\...\D1_MSMS_985.5645_5.t2d
Acquired:
