# Supplementary material for: Physiological, Ultrastructural and Proteomic Responses in the Leaf of Maize Seedlings to Polyethylene Glycol-Stimulated Severe Water Deficiency
Source: Int J Mol Sci. 2015 Sep 8;16(9):21606–25. doi: 10.3390/ijms160921606 (PMC4613270; doi:10.3390/ijms160921606)
Supplement: Supplementary file 1 [file ijms-16-21606-s001.zip › ijms-96220-Supplementary Information/Supplementary File S2/MSMS-PDF/spot 7-C4.pdf]

4700 MS/MS Precursor 1498.63 Spec #1 MC[BP = 175.1, 520]

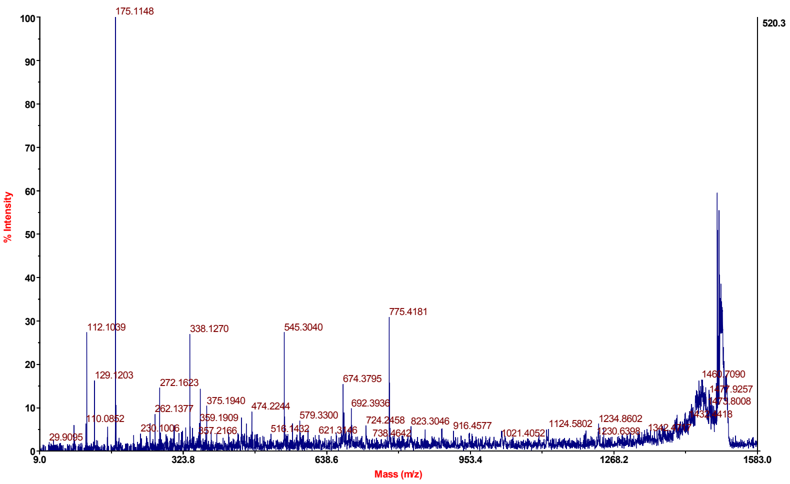

E:\...\C4_MSMS_1498.6270_11.t2d
Acquired:

4700 MS/MS Precursor 1446.64 Spec #1 MC[BP = 175.1, 2055]

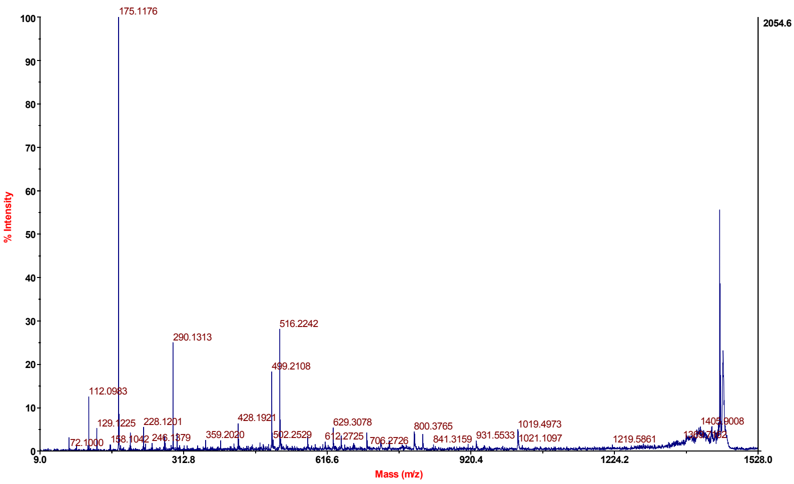

E:\...\C4_MSMS_1446.6442_4.t2d
Acquired:

4700 MS/MS Precursor 1397.66 Spec #1 MC[BP = 1397.7, 1160]

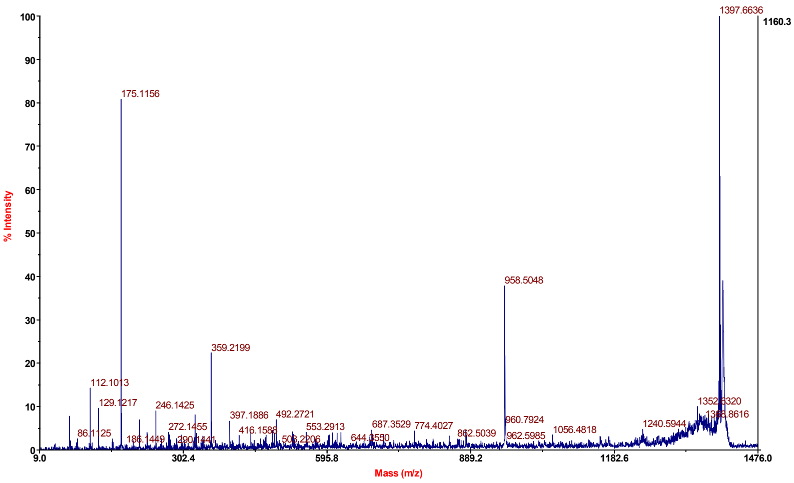

E:\...\C4_MSMS_1397.6580_5.t2d
Acquired:

4700 MS/MS Precursor 1374.62 Spec #1 MC[BP = 1374.6, 925]

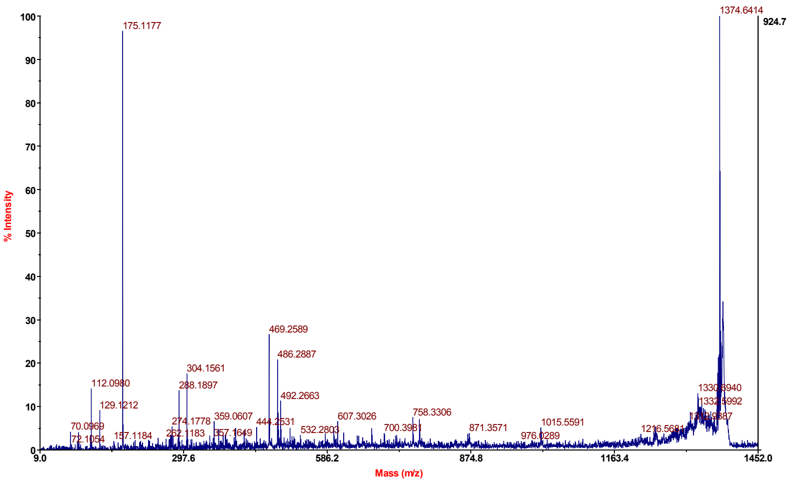

E:\...\C4_MSMS_1374.6179_6.t2d
Acquired:

4700 MS/MS Precursor 1208.54 Spec #1 MC[BP = 175.1, 886]

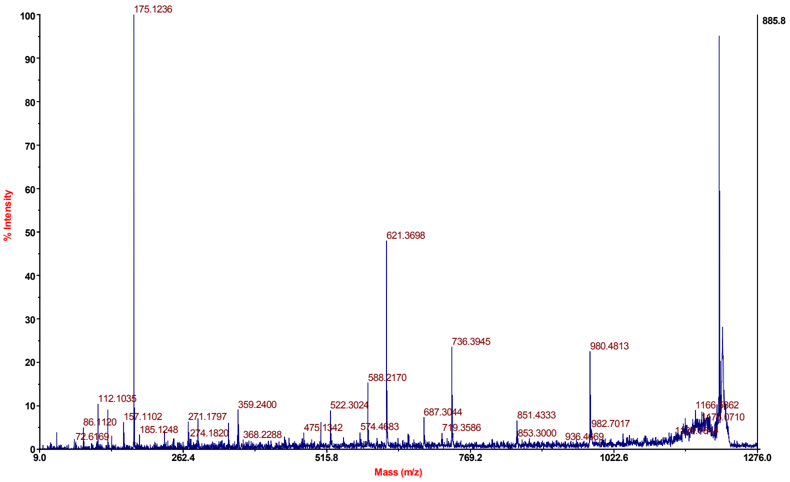

E:\...\C4_MSMS_1208.5393_8.t2d
Acquired:

4700 MS/MS Precursor 1078.59 Spec #1 MC[BP = 175.1, 621]

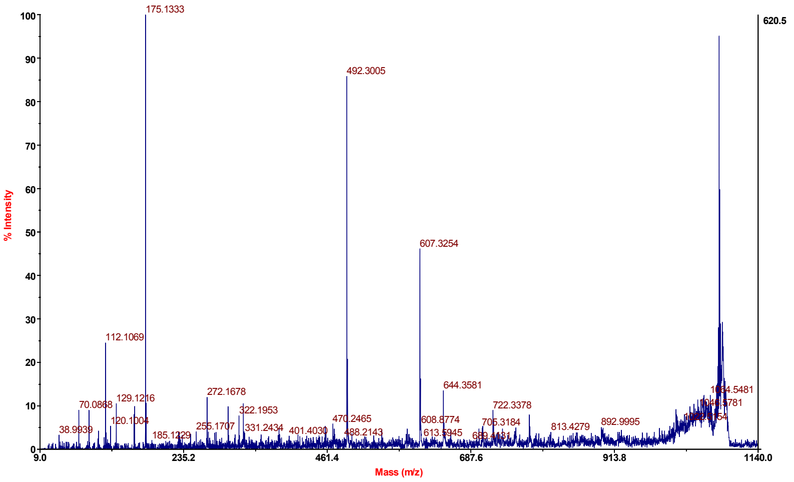

E:\...\C4_MSMS_1078.5856_7.t2d
Acquired:

4700 MS/MS Precursor 1065.58 Spec #1 MC[BP = 1066.5, 507]

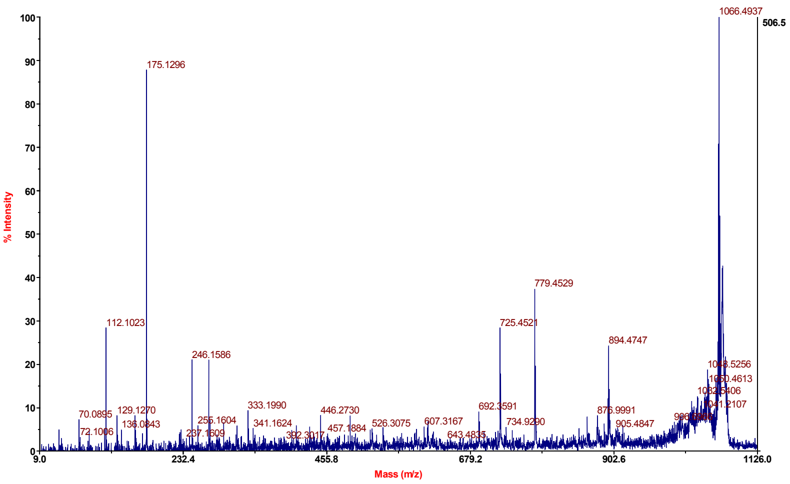

E:\...\C4_MSMS_1065.5833_9.t2d
Acquired:

4700 MS/MS Precursor 869.443 Spec #1 MC[BP = 869.4, 5969]

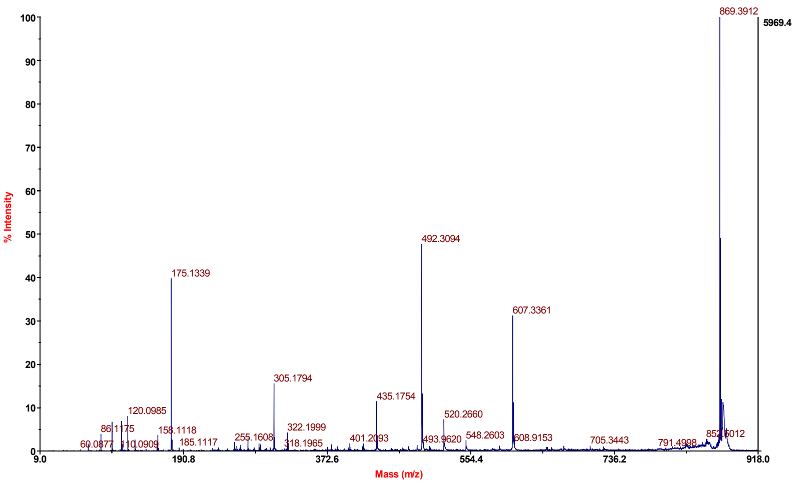

E:\...\C4_MSMS_869.4431_3.t2d
Acquired:
