# Supplementary material for: Physiological, Ultrastructural and Proteomic Responses in the Leaf of Maize Seedlings to Polyethylene Glycol-Stimulated Severe Water Deficiency
Source: Int J Mol Sci. 2015 Sep 8;16(9):21606–25. doi: 10.3390/ijms160921606 (PMC4613270; doi:10.3390/ijms160921606)
Supplement: Supplementary file 1 [file ijms-16-21606-s001.zip › ijms-96220-Supplementary Information/Supplementary File S2/MSMS-PDF/spot 9-D2.pdf]

4700 MS/MS Precursor 1254.74 Spec #1 MC[BP = 1254.7, 129]

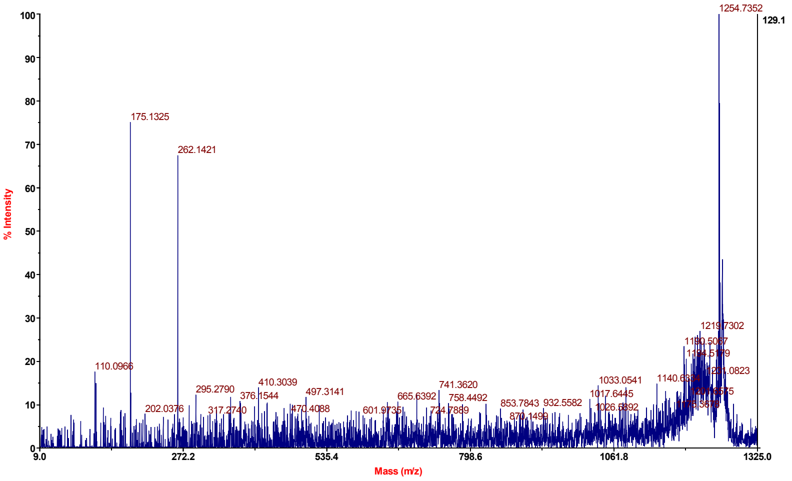

E:\...\D2_MSMS_1254.7397_5.t2d
Acquired:

4700 MS/MS Precursor 1083.61 Spec #1 MC[BP = 304.2, 76]

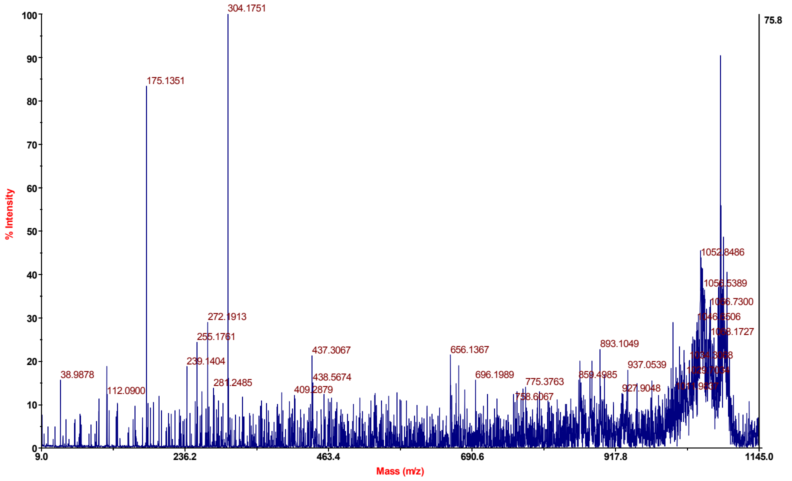

E:\...\D2_MSMS_1083.6082_9.t2d
Acquired:

4700 MS/MS Precursor 1067.67 Spec #1 MC[BP = 1067.7, 312]

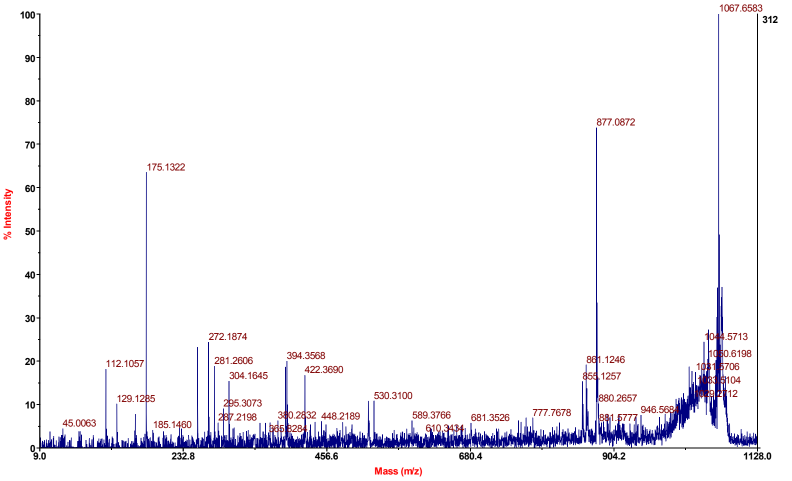

E:\...\D2_MSMS_1067.6696_4.t2d
Acquired:

4700 MS/MS Precursor 1051.67 Spec #1 MC[BP = 1051.7, 853]

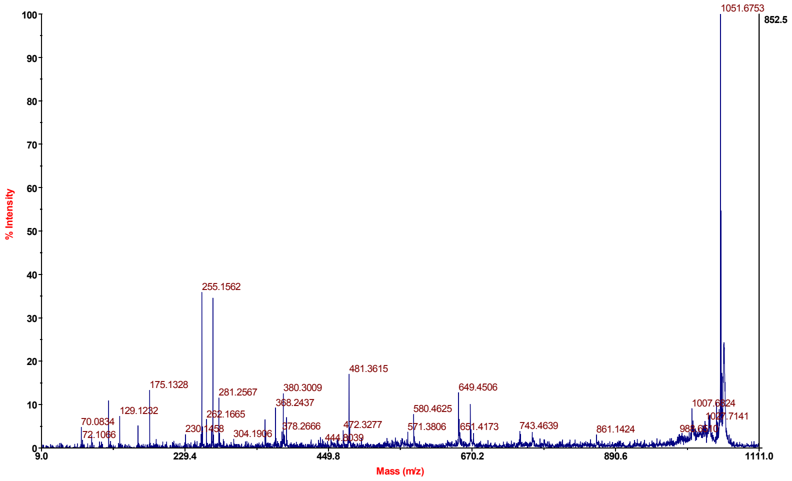

E:\...\D2_MSMS_1051.6683_2.t2d
Acquired:

4700 MS/MS Precursor 996.594 Spec #1 MC[BP = 995.6, 151]

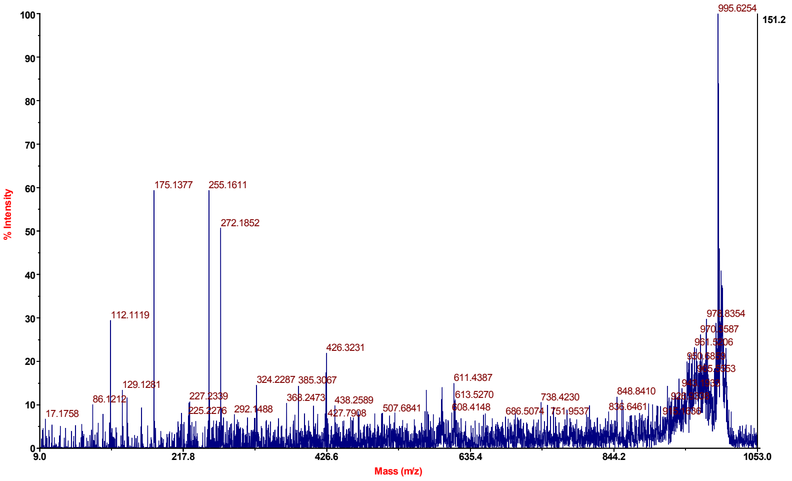

E:\...\D2_MSMS_996.5943_8.t2d
Acquired:

4700 MS/MS Precursor 870.552 Spec #1 MC[BP = 870.5, 600]

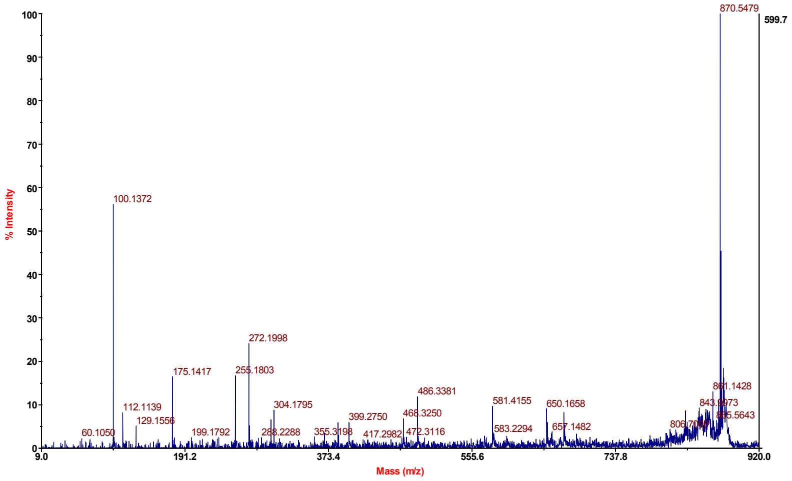

E:\...\D2_MSMS_870.5522_3.t2d
Acquired:

4700 MS/MS Precursor 858.511 Spec #1 MC[BP = 672.0, 300]

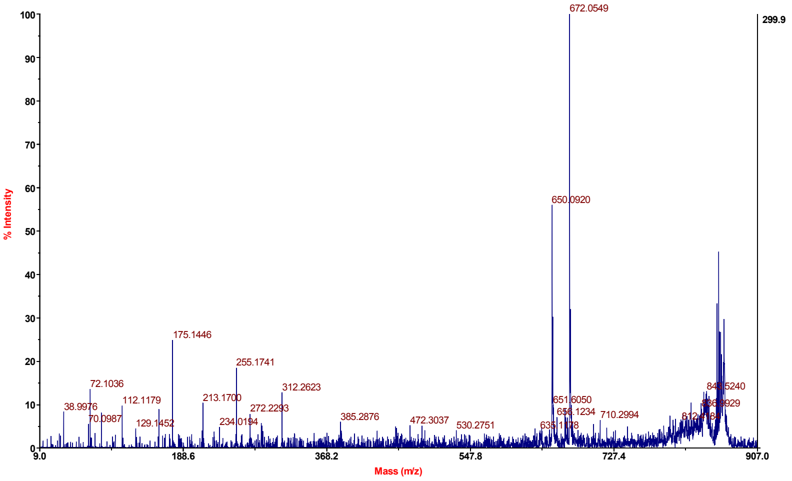

E:\...\D2_MSMS_858.5111_10.t2d
Acquired:

4700 MS/MS Precursor 800.495 Spec #1 MC[BP = 755.6, 279]

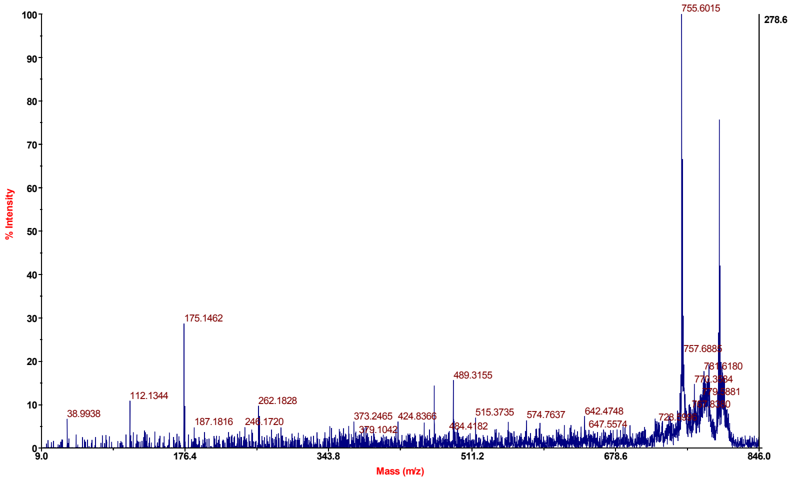

E:\...\D2_MSMS_800.4950_7.t2d
Acquired:
