# Supplementary figures and images for: Physiological, Ultrastructural and Proteomic Responses in the Leaf of Maize Seedlings to Polyethylene Glycol-Stimulated Severe Water Deficiency
Source: Int J Mol Sci. 2015 Sep 8;16(9):21606–25. doi: 10.3390/ijms160921606 (PMC4613270; doi:10.3390/ijms160921606)

# Applied Biosystems 4700 Proteomics Analyzer 7044

4700 Reflector Spec #1 MC[BP = 1287.7, 23059]

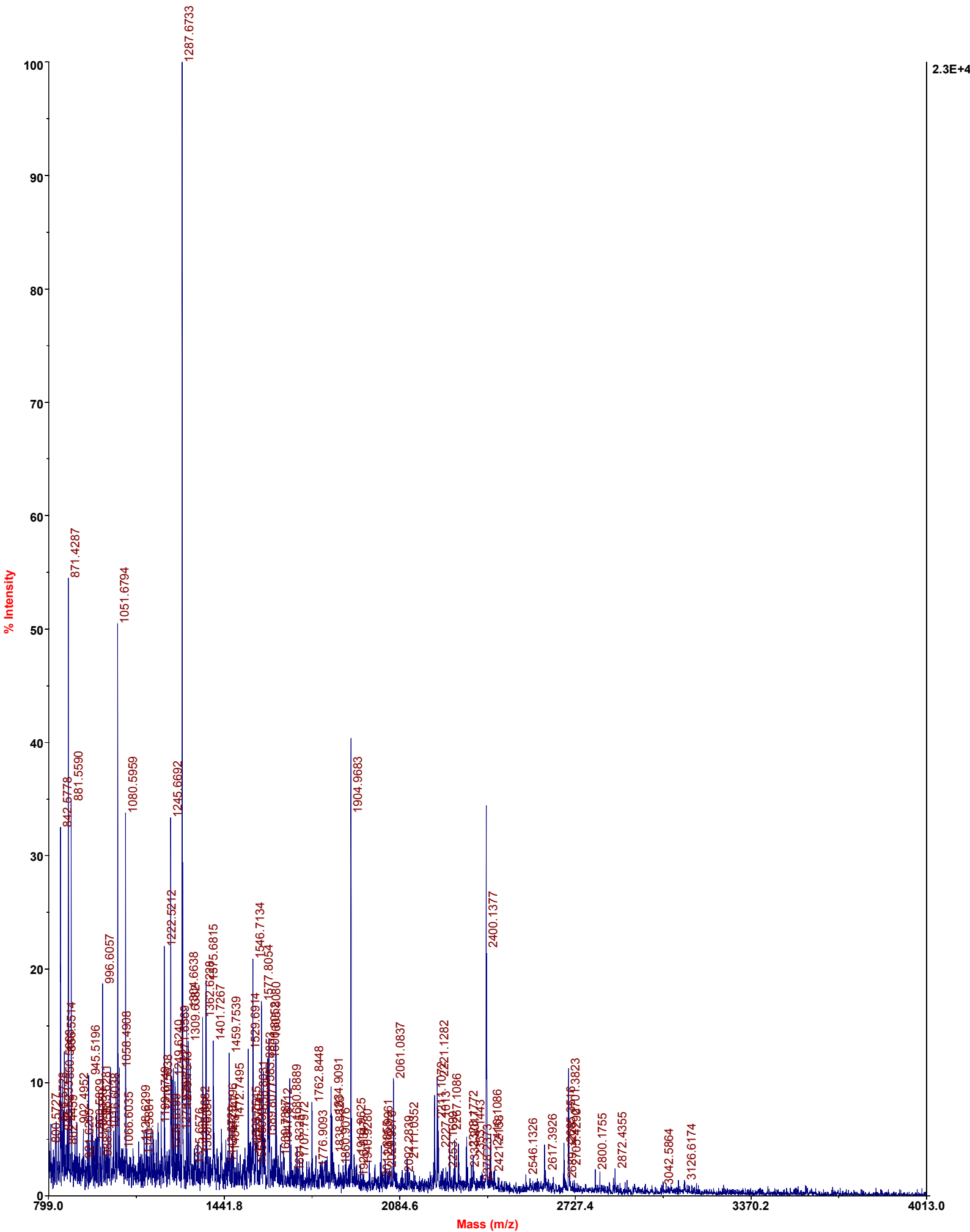

Supplement: Supplementary file 1 [file ijms-16-21606-s001.zip › ijms-96220-Supplementary Information/Supplementary File S1/MS-PDF/spot 10-C12.pdf]

# Applied Biosystems 4700 Proteomics Analyzer 7044

4700 Reflector Spec #1 MC[BP = 1156.6, 15325]

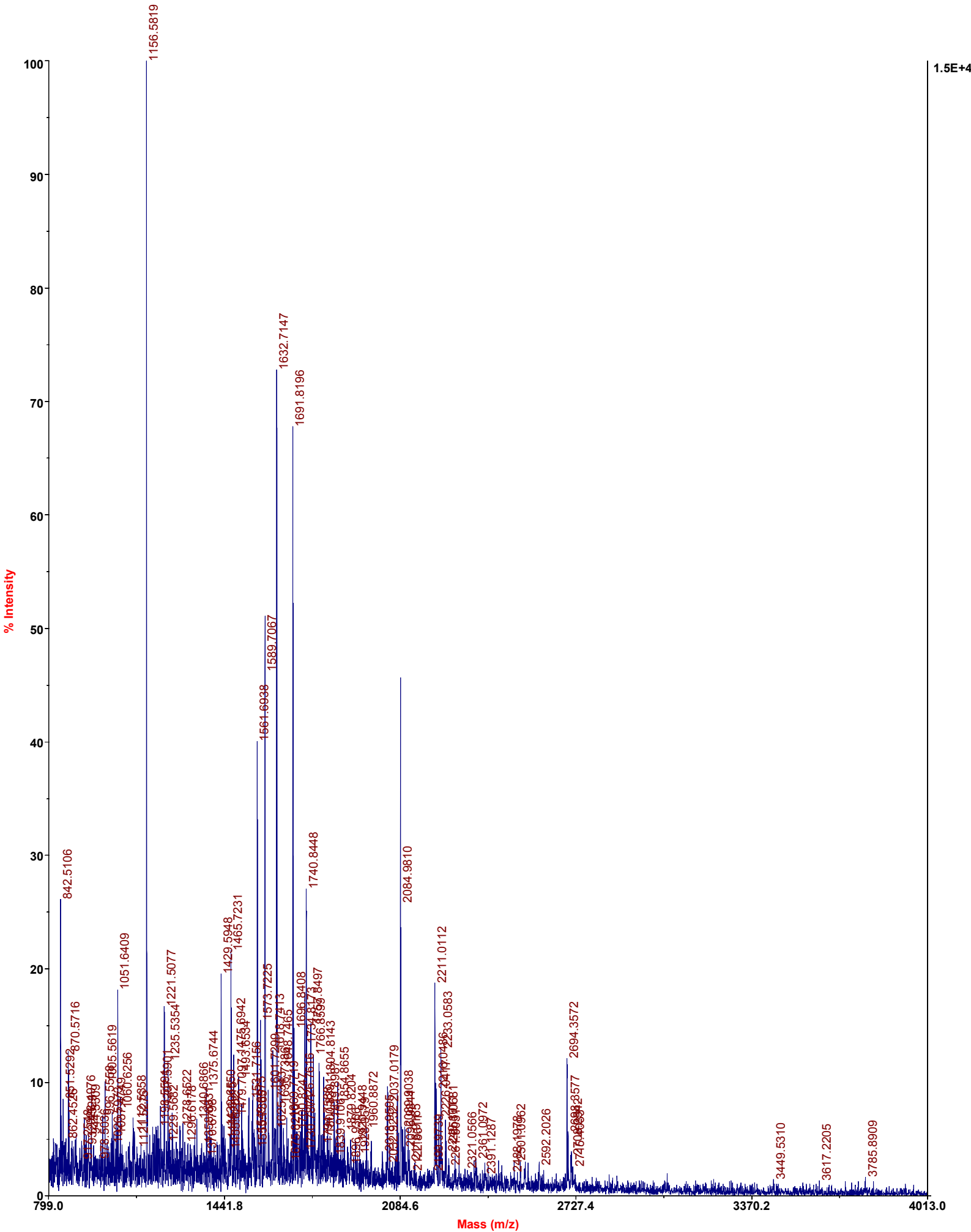

Printed: 13:02, March 31, 2014

Supplement: Supplementary file 1 [file ijms-16-21606-s001.zip › ijms-96220-Supplementary Information/Supplementary File S1/MS-PDF/spot 12-C8.pdf]

4700 Reflector Spec #1 MC[BP = 1051.7, 62855]

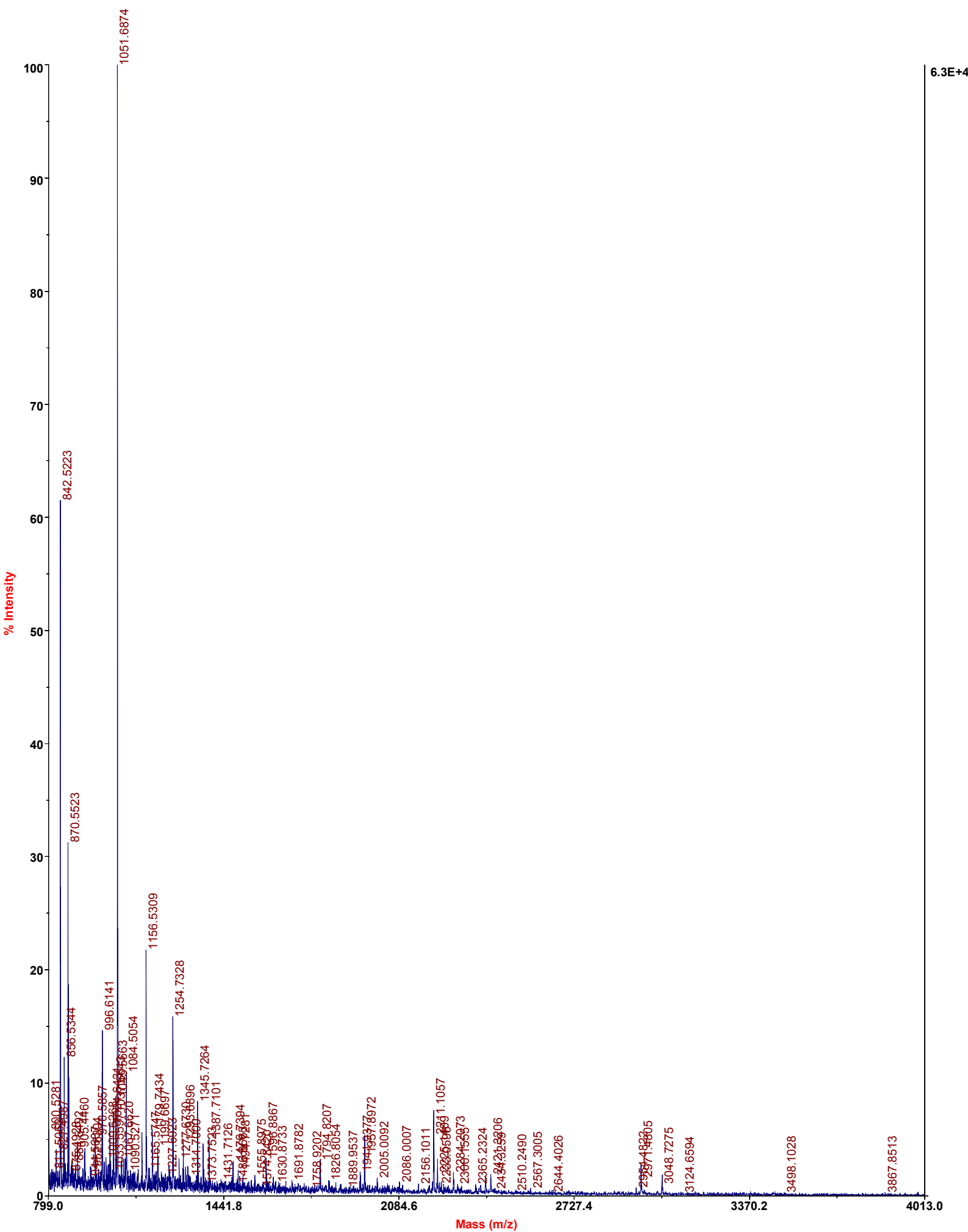

Supplement: Supplementary file 1 [file ijms-16-21606-s001.zip › ijms-96220-Supplementary Information/Supplementary File S1/MS-PDF/spot 13-C11.pdf]

4700 Reflector Spec #1 MC[BP = 1884.8, 40596]

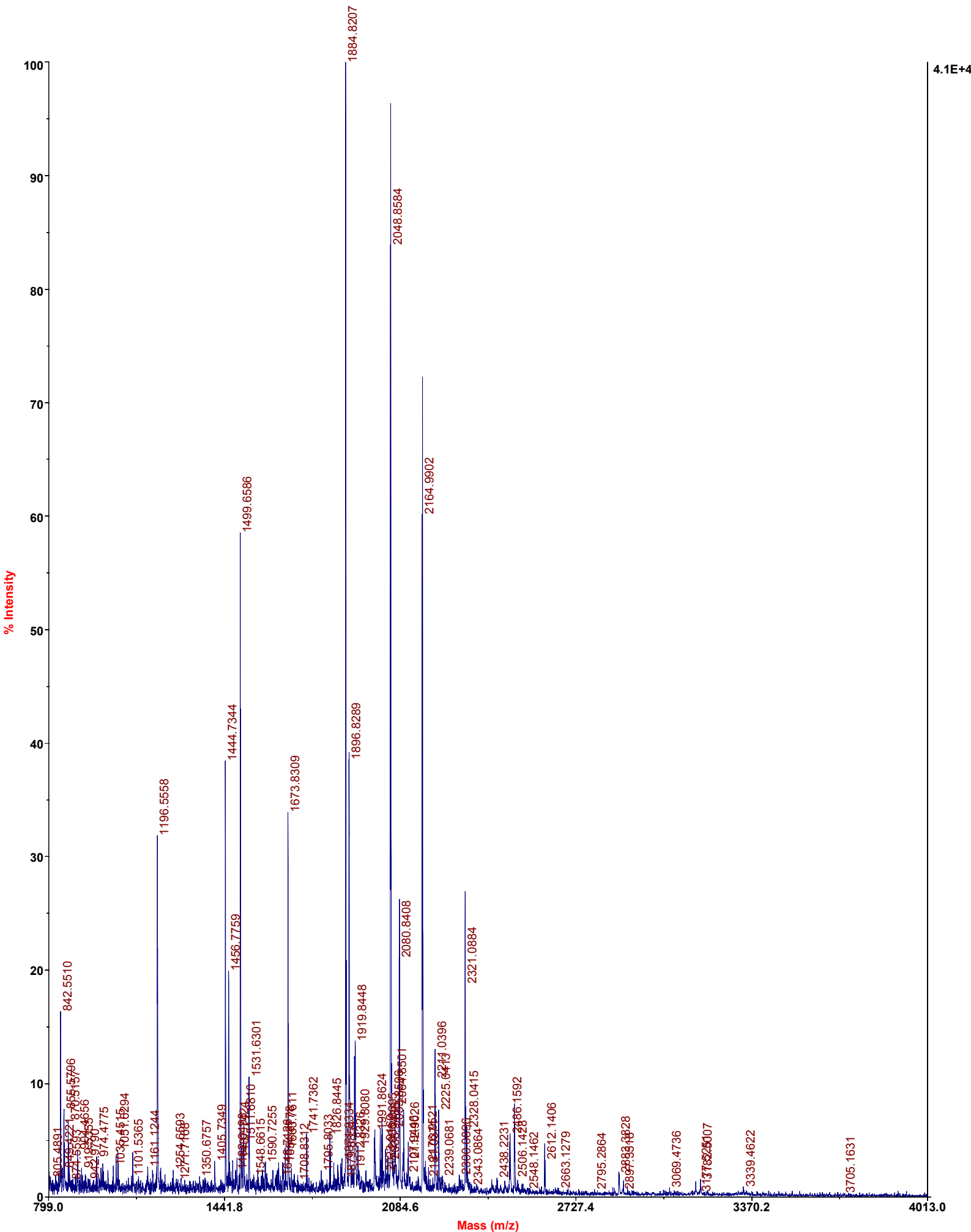

Supplement: Supplementary file 1 [file ijms-16-21606-s001.zip › ijms-96220-Supplementary Information/Supplementary File S1/MS-PDF/spot 14-C9.pdf]

4700 Reflector Spec #1 MC[BP = 1250.5, 49914]

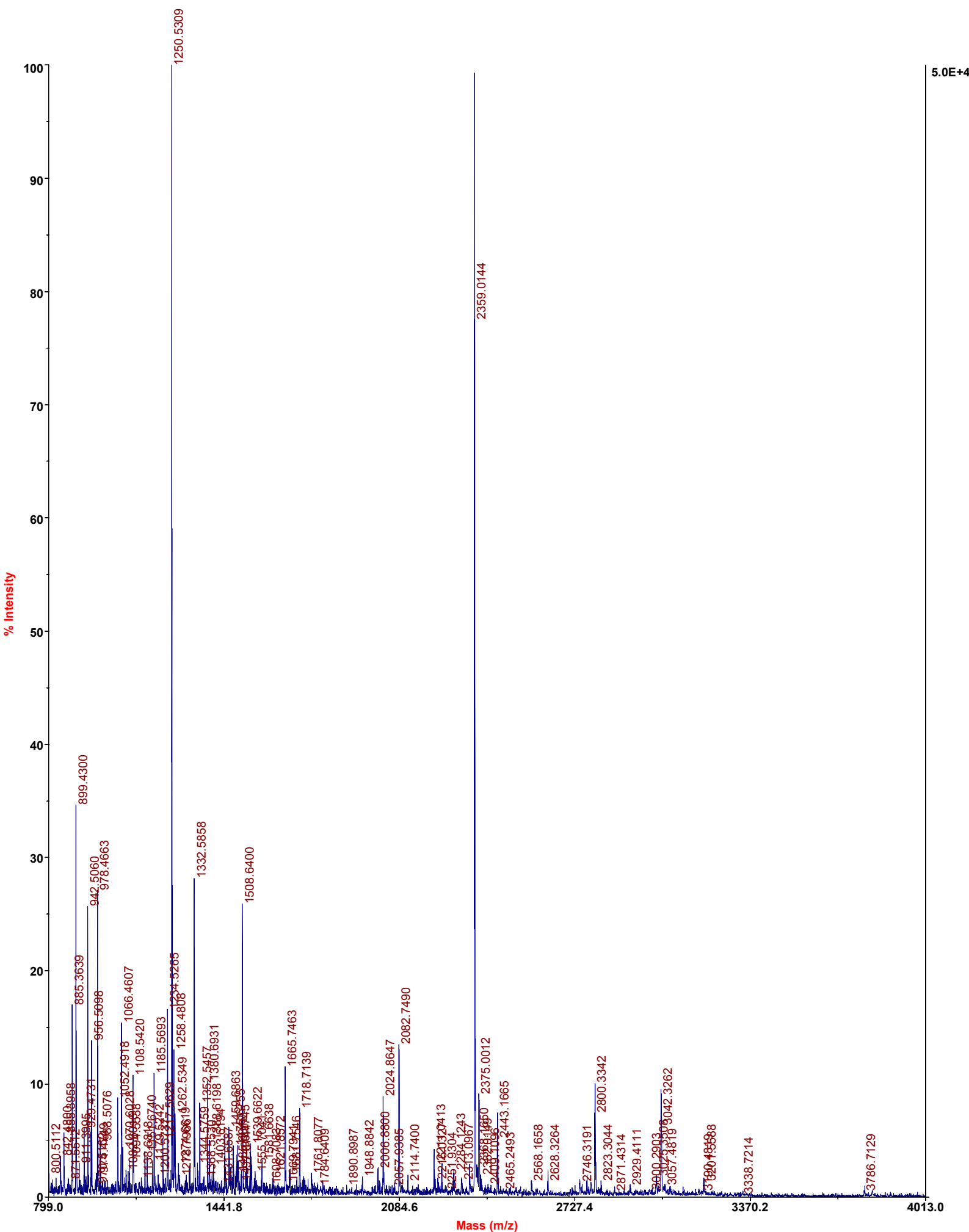

Supplement: Supplementary file 1 [file ijms-16-21606-s001.zip › ijms-96220-Supplementary Information/Supplementary File S1/MS-PDF/spot 15-C7.pdf]

4700 Reflector Spec #1 MC[BP = 1465.7, 19059]

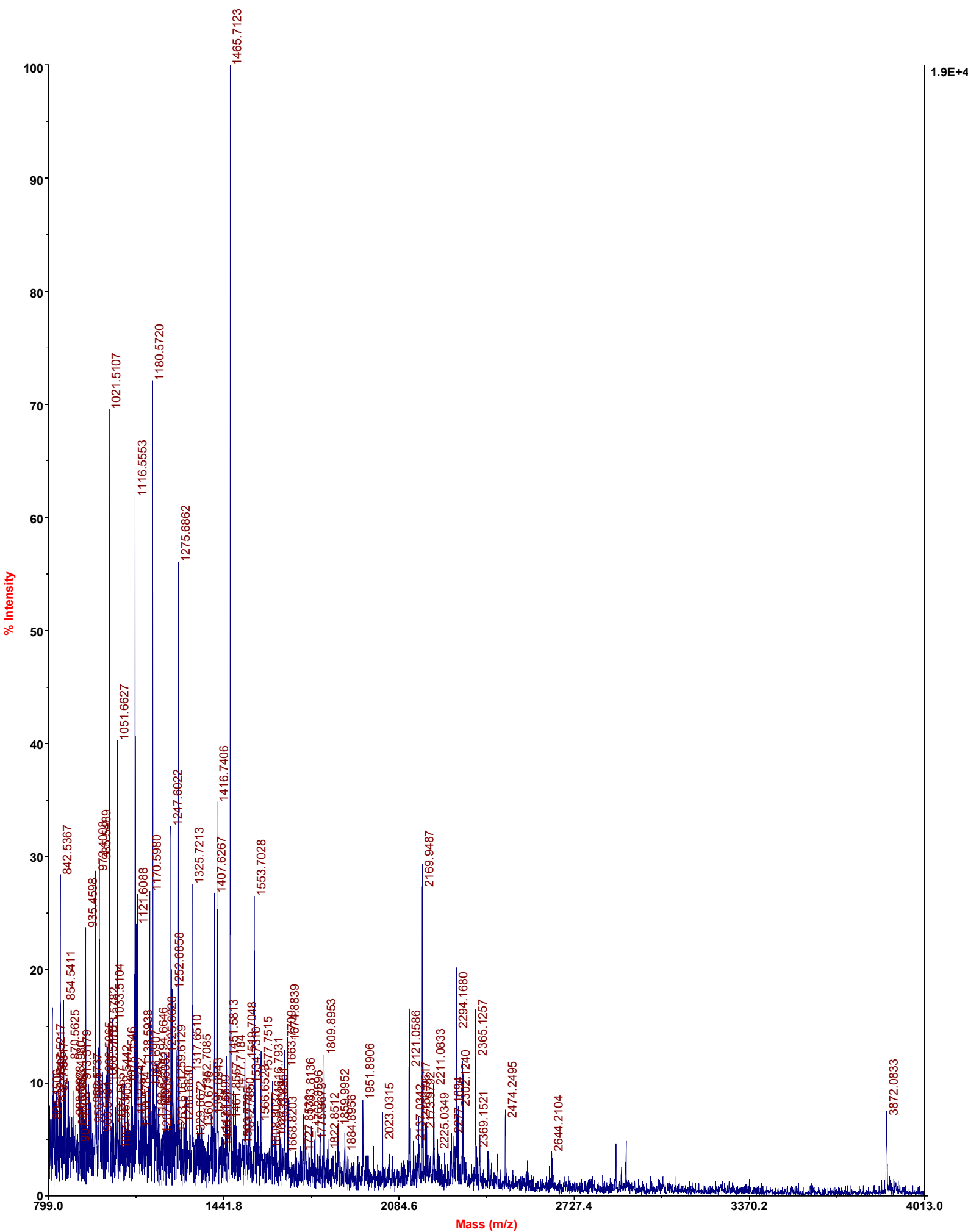

Supplement: Supplementary file 1 [file ijms-16-21606-s001.zip › ijms-96220-Supplementary Information/Supplementary File S1/MS-PDF/spot 16-C3.pdf]

4700 Reflector Spec #1 MC[BP = 1120.5, 4769]

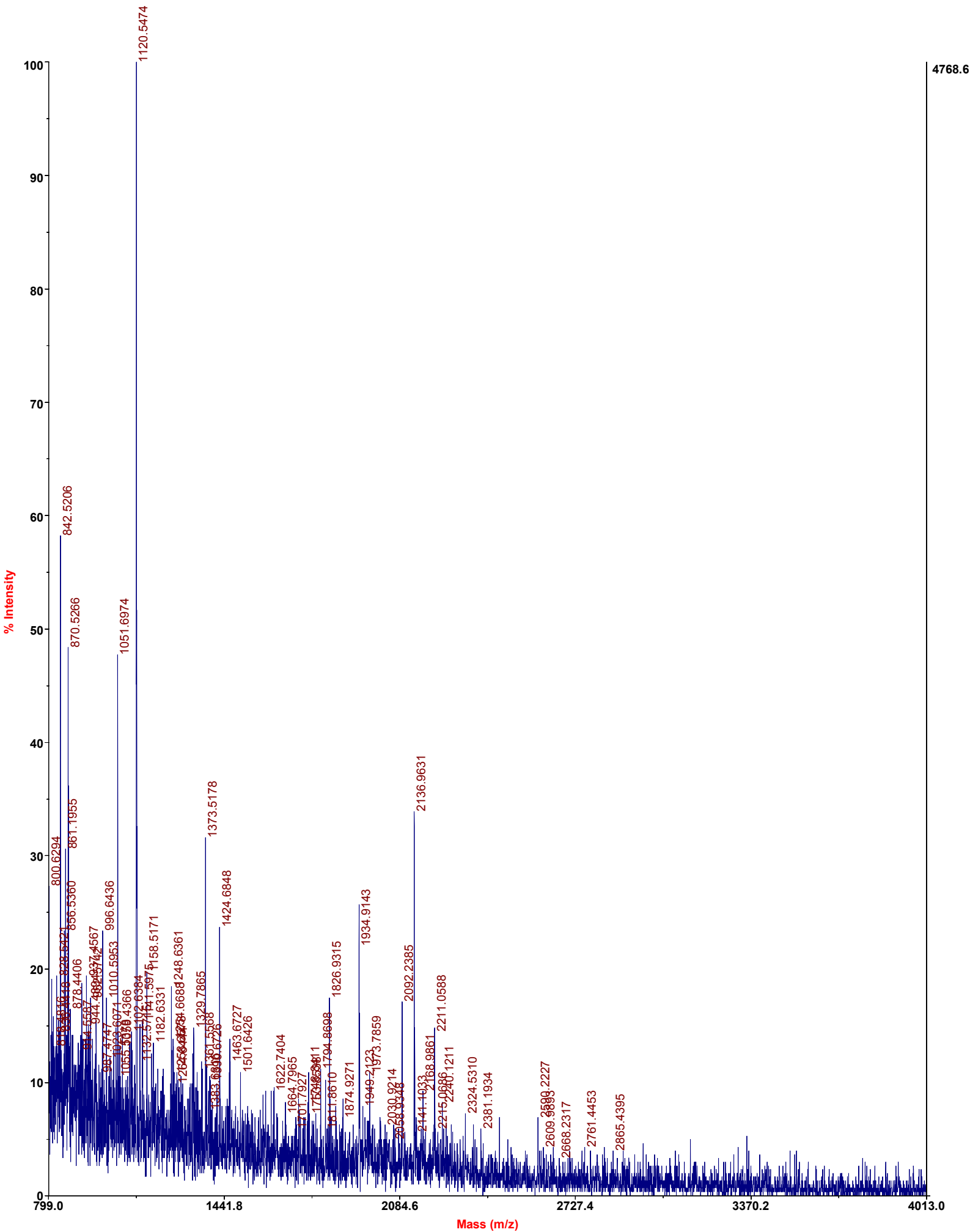

Supplement: Supplementary file 1 [file ijms-16-21606-s001.zip › ijms-96220-Supplementary Information/Supplementary File S1/MS-PDF/spot 17-D4.pdf]

4700 Reflector Spec #1 MC[BP = 1002.5, 12235]

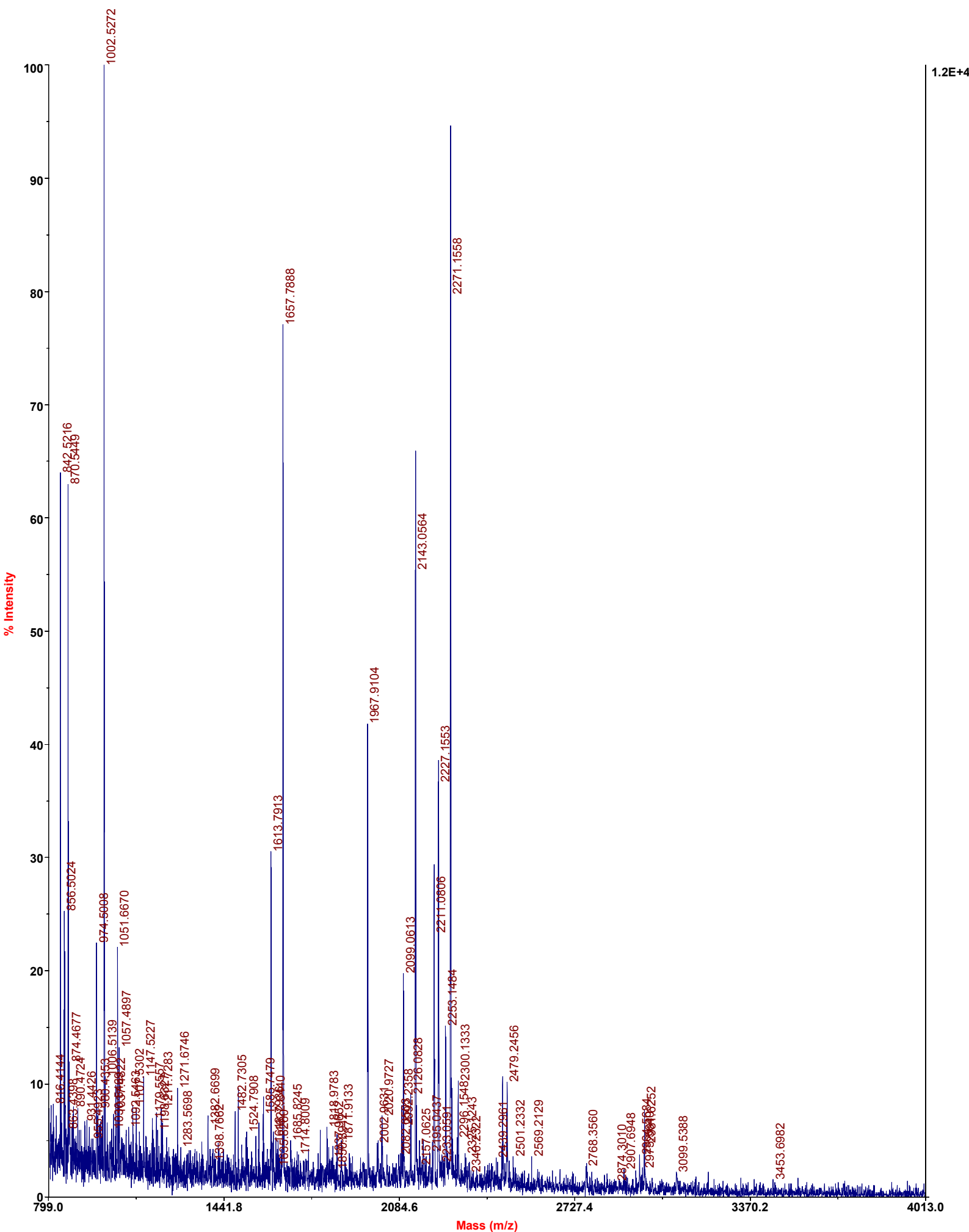

Supplement: Supplementary file 1 [file ijms-16-21606-s001.zip › ijms-96220-Supplementary Information/Supplementary File S1/MS-PDF/spot 2-D3.pdf]

4700 Reflector Spec #1 MC[BP = 2228.2, 89020]

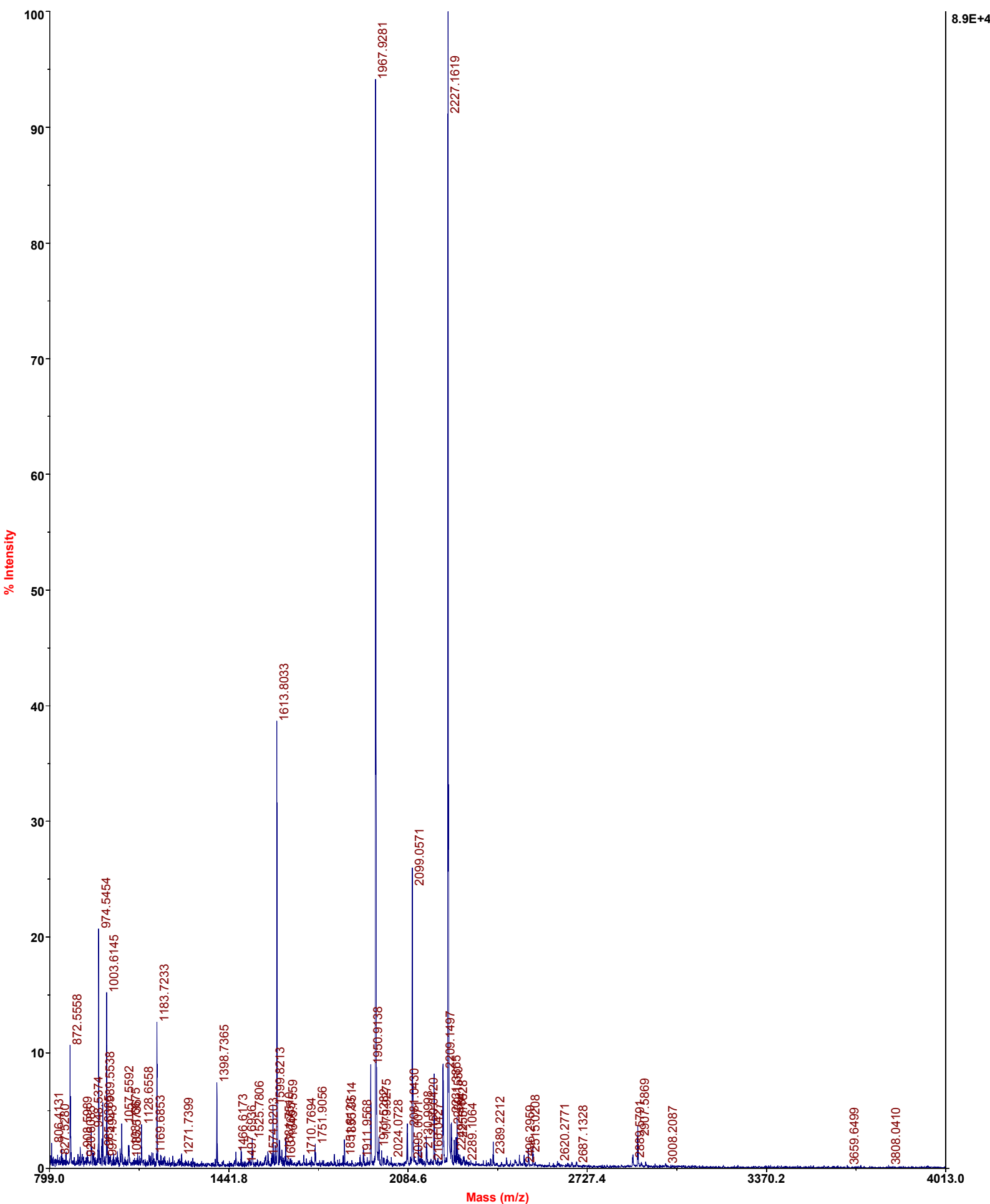

Supplement: Supplementary file 1 [file ijms-16-21606-s001.zip › ijms-96220-Supplementary Information/Supplementary File S1/MS-PDF/spot 3-D5.pdf]

4700 Reflector Spec #1 MC[BP = 1207.6, 36031]

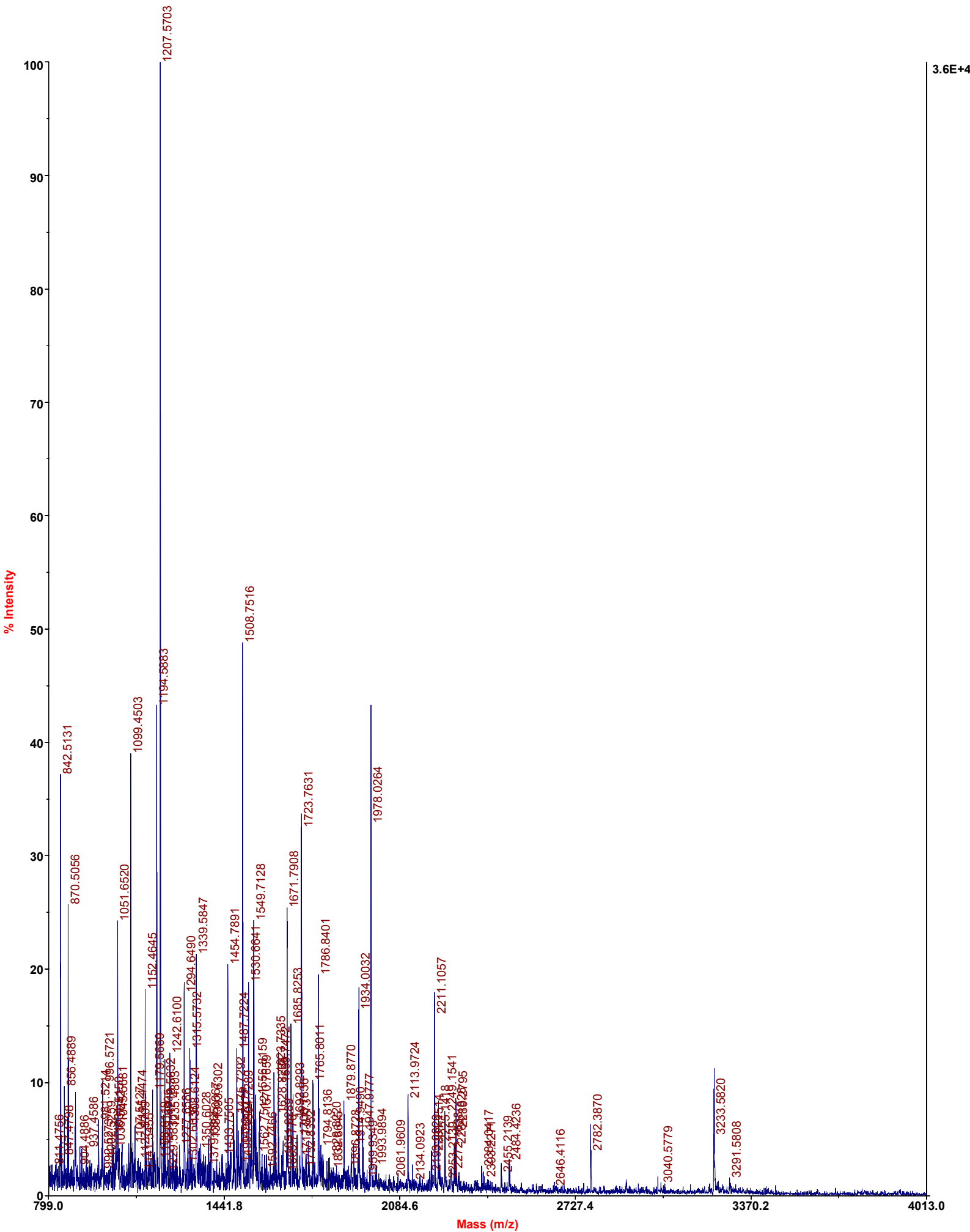

Supplement: Supplementary file 1 [file ijms-16-21606-s001.zip › ijms-96220-Supplementary Information/Supplementary File S1/MS-PDF/spot 4-C6.pdf]

4700 Reflector Spec #1 MC[BP = 1021.5, 24941]

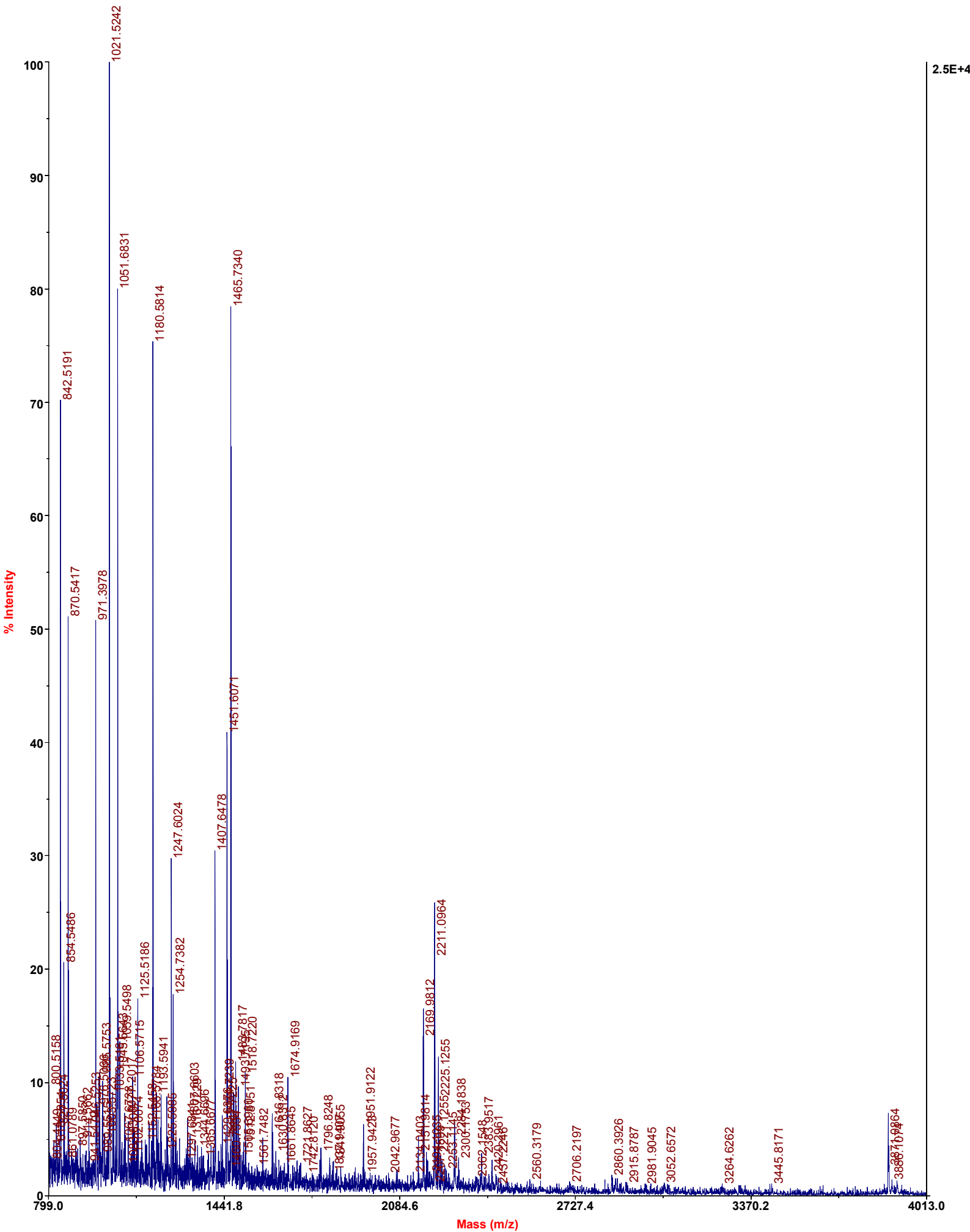

Supplement: Supplementary file 1 [file ijms-16-21606-s001.zip › ijms-96220-Supplementary Information/Supplementary File S1/MS-PDF/spot 5-C10.pdf]

4700 Reflector Spec #1 MC[BP = 1021.5, 66447]

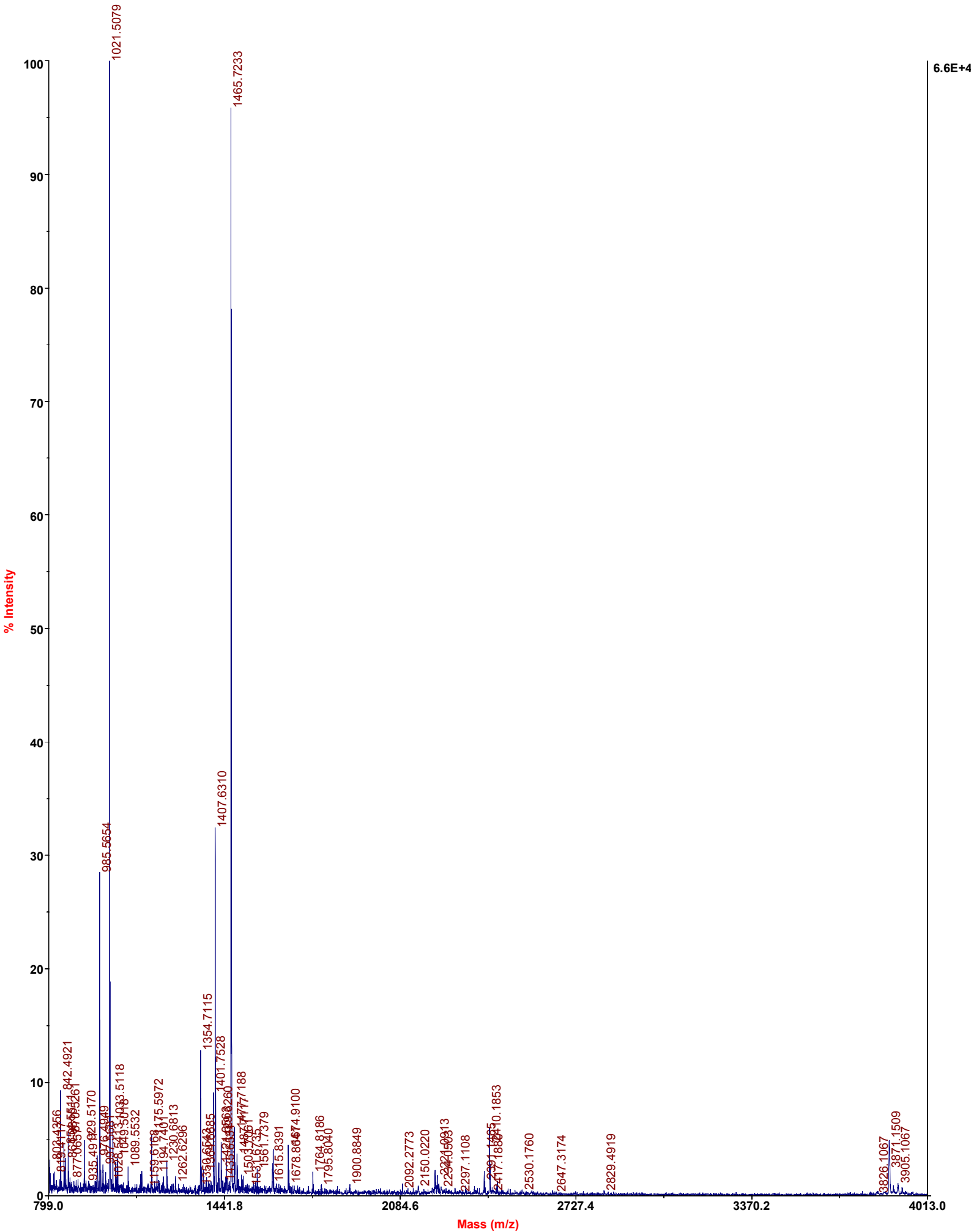

Supplement: Supplementary file 1 [file ijms-16-21606-s001.zip › ijms-96220-Supplementary Information/Supplementary File S1/MS-PDF/spot 6-D1.pdf]

4700 Reflector Spec #1 MC[BP = 2318.0, 15529]

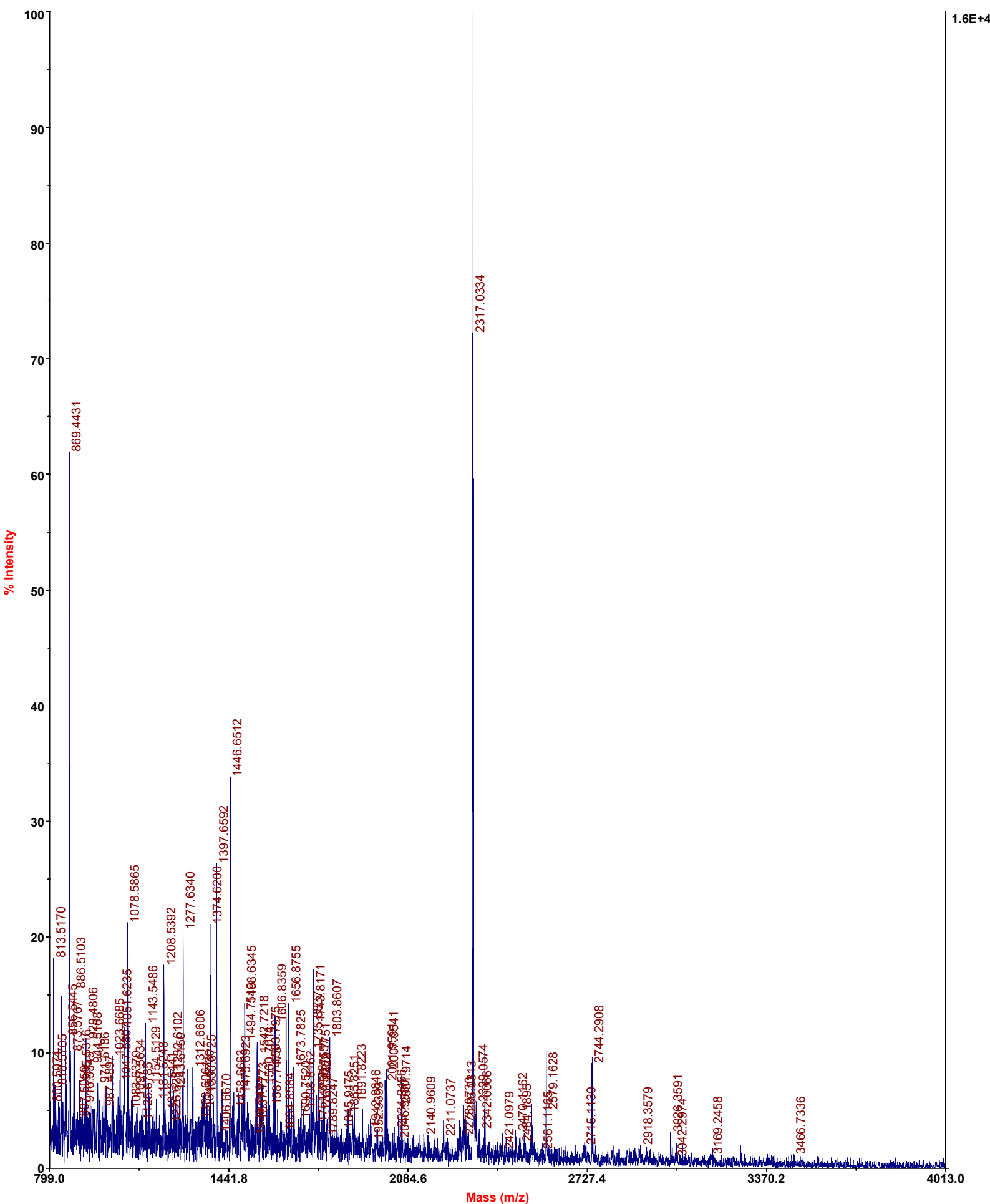

Supplement: Supplementary file 1 [file ijms-16-21606-s001.zip › ijms-96220-Supplementary Information/Supplementary File S1/MS-PDF/spot 7-C4.pdf]

4700 Reflector Spec #1 MC[BP = 1051.7, 30243]

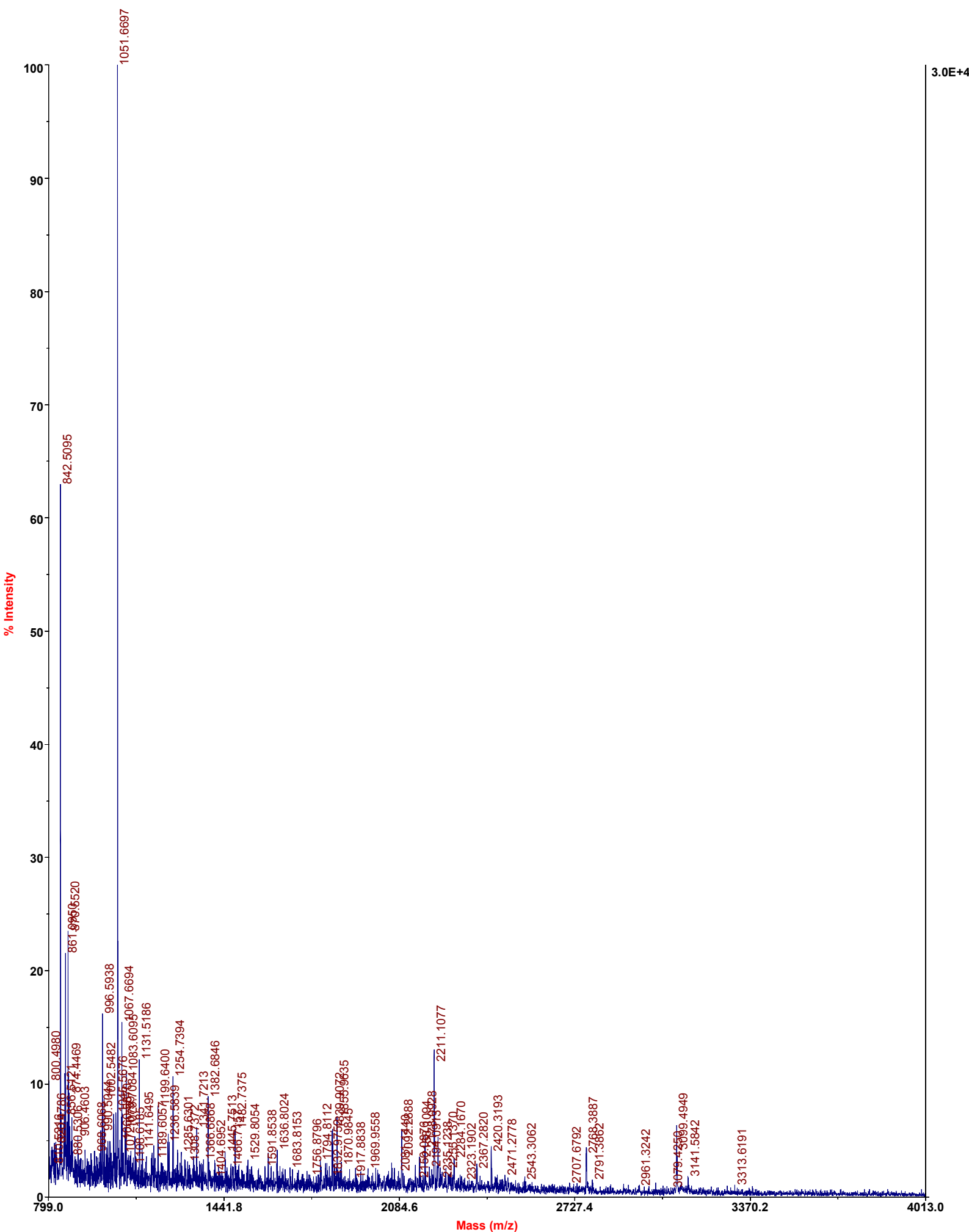

Supplement: Supplementary file 1 [file ijms-16-21606-s001.zip › ijms-96220-Supplementary Information/Supplementary File S1/MS-PDF/spot 9-D2.pdf]

4700 Reflector Spec #1 MC[BP = 1393.6, 29145]

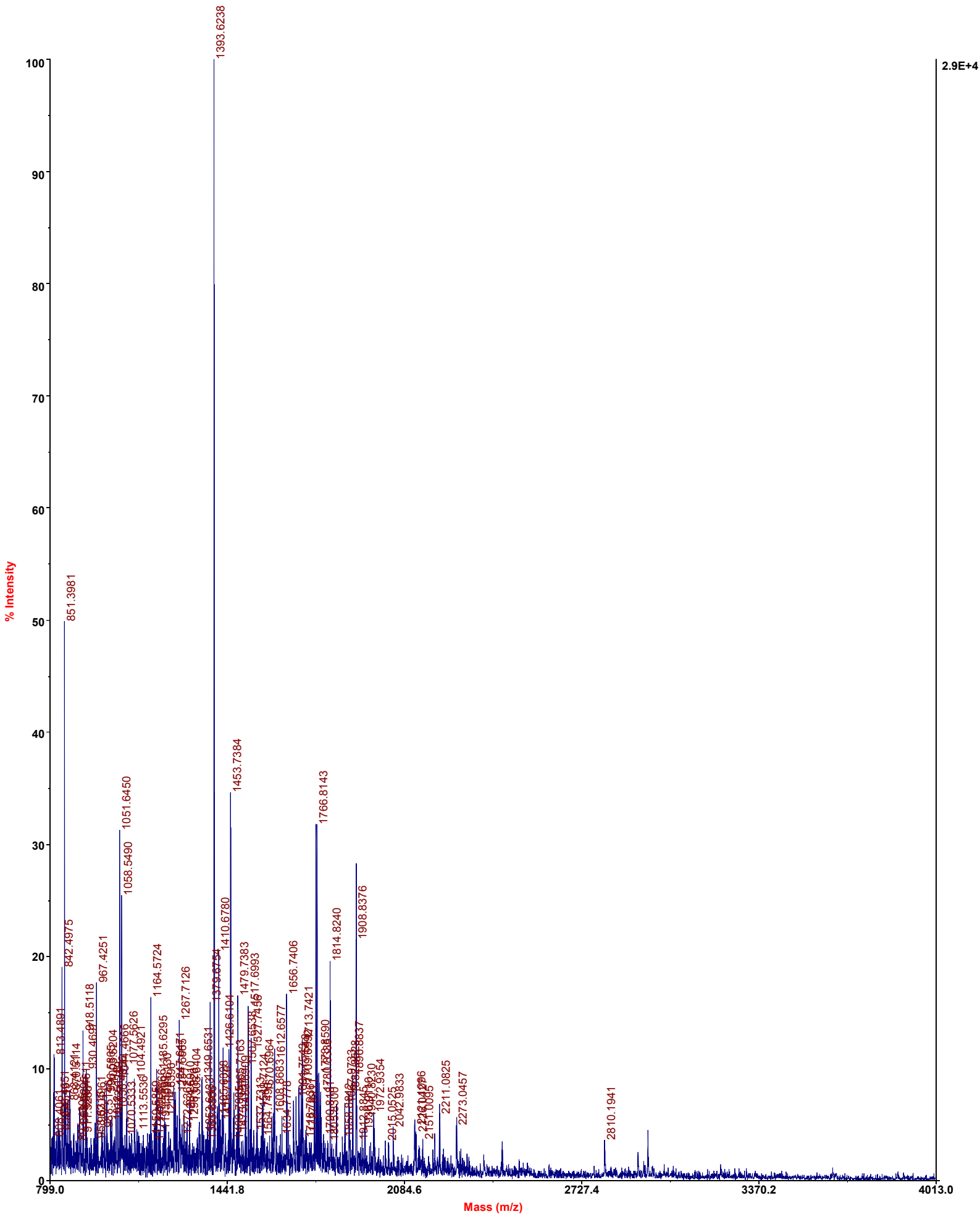

Supplement: Supplementary file 1 [file ijms-16-21606-s001.zip › ijms-96220-Supplementary Information/Supplementary File S1/MS-PDF/spot1-C2.pdf]

4700 MS/MS Precursor 870.573 Spec #1 MC[BP = 870.5, 277]

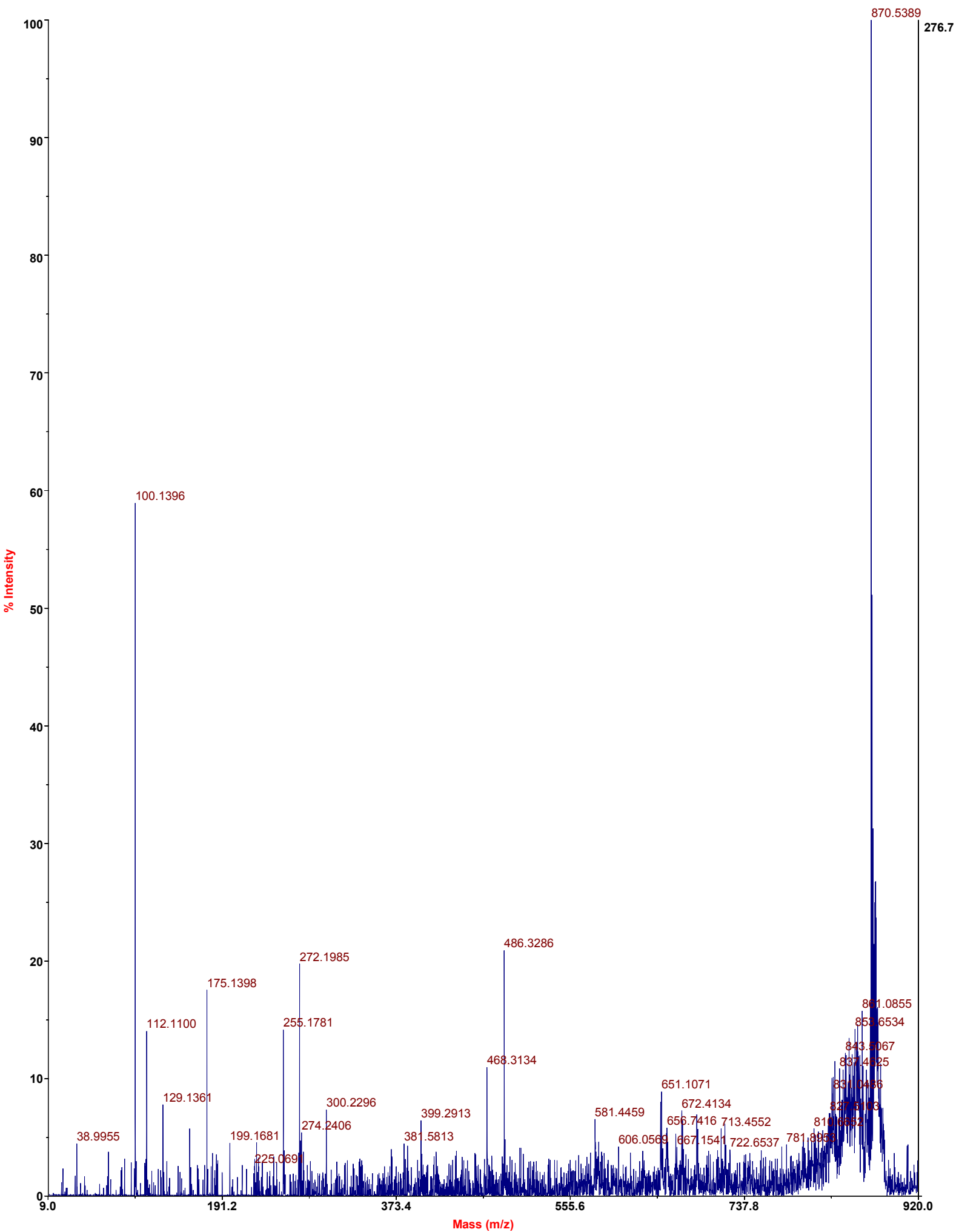

Supplement: Supplementary file 1 [file ijms-16-21606-s001.zip › ijms-96220-Supplementary Information/Supplementary File S2/MSMS-PDF/spot 12-C8.pdf]

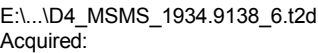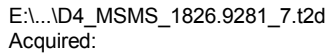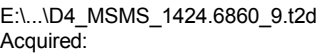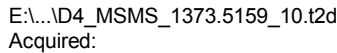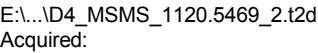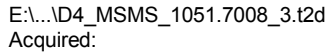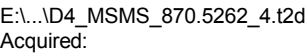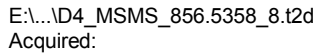

Supplement: Supplementary file 1 [file ijms-16-21606-s001.zip › ijms-96220-Supplementary Information/Supplementary File S2/MSMS-PDF/spot 17-D4.pdf]

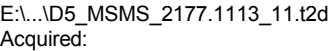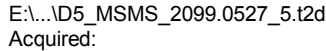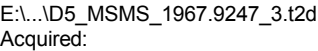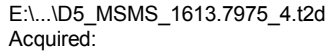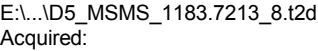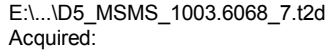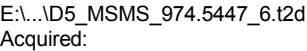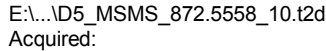

Supplement: Supplementary file 1 [file ijms-16-21606-s001.zip › ijms-96220-Supplementary Information/Supplementary File S2/MSMS-PDF/spot 3-D5.pdf]

4700 MS/MS Precursor 870.505 Spec #1 MC[BP = 870.6, 1428]

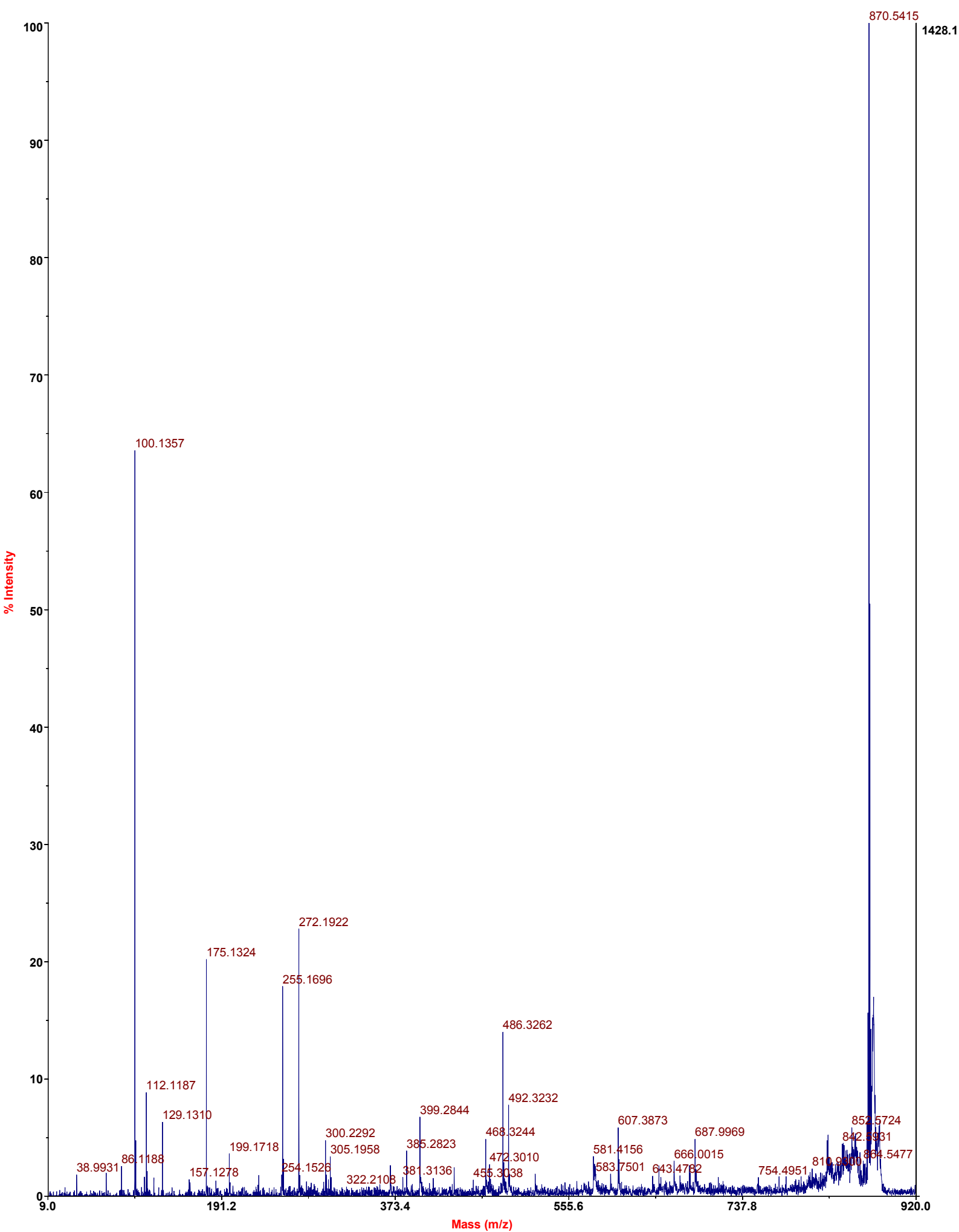

Supplement: Supplementary file 1 [file ijms-16-21606-s001.zip › ijms-96220-Supplementary Information/Supplementary File S2/MSMS-PDF/spot 4-C6.pdf]
